# Supplementary material for: Backbone rigidity of disordered protein linkers from NMR experiments and MD simulations
Source: Biophys J. 2026 Mar 10;125(7):1753–64. doi: 10.1016/j.bpj.2026.03.009 (PMC13351908; doi:10.1016/j.bpj.2026.03.009)
Supplement: Document S1. Figures S1–S49 and Table S1 [file mmc1.pdf]

Biophysical Journal, Volume *125*

Supplemental information

Backbone rigidity of disordered protein linkers from NMR experiments and MD simulations

Efstathia Mantzari, Cajsa K. Malm, Ricky Nencini, Amanda E. Sandelin, and O.H. Samuli Ollila

Supplementary information: Backbone rigidity of disordered protein linkers from NMR experiments and MD simulations

Efstathia Mantzari,[†,‡,¶] Cajsa K. Malm,[†] Ricky Nencini,[‡,§] Amanda E. Sandelin,[‖,‡] and O. H. Samuli Ollila[*,†,‡]

†*VTT Technical Research Centre of Finland, 02044 Espoo, Finland*

‡*Institute of Biotechnology, University of Helsinki, 00014 Helsinki, Finland*

¶*Department of Chemistry and Materials Science, Aalto University, 00076 Espoo, Finland*

§*Division of Pharmaceutical Biosciences, Faculty of Pharmacy, University of Helsinki, 00014 Helsinki, Finland*

‖*Division of Pharmacology and Pharmacotherapy, Faculty of Pharmacy, University of Helsinki, 00014 Helsinki, Finland*

E-mail: samuli.ollila@vtt.fi

Supplementary results

NMR experiments

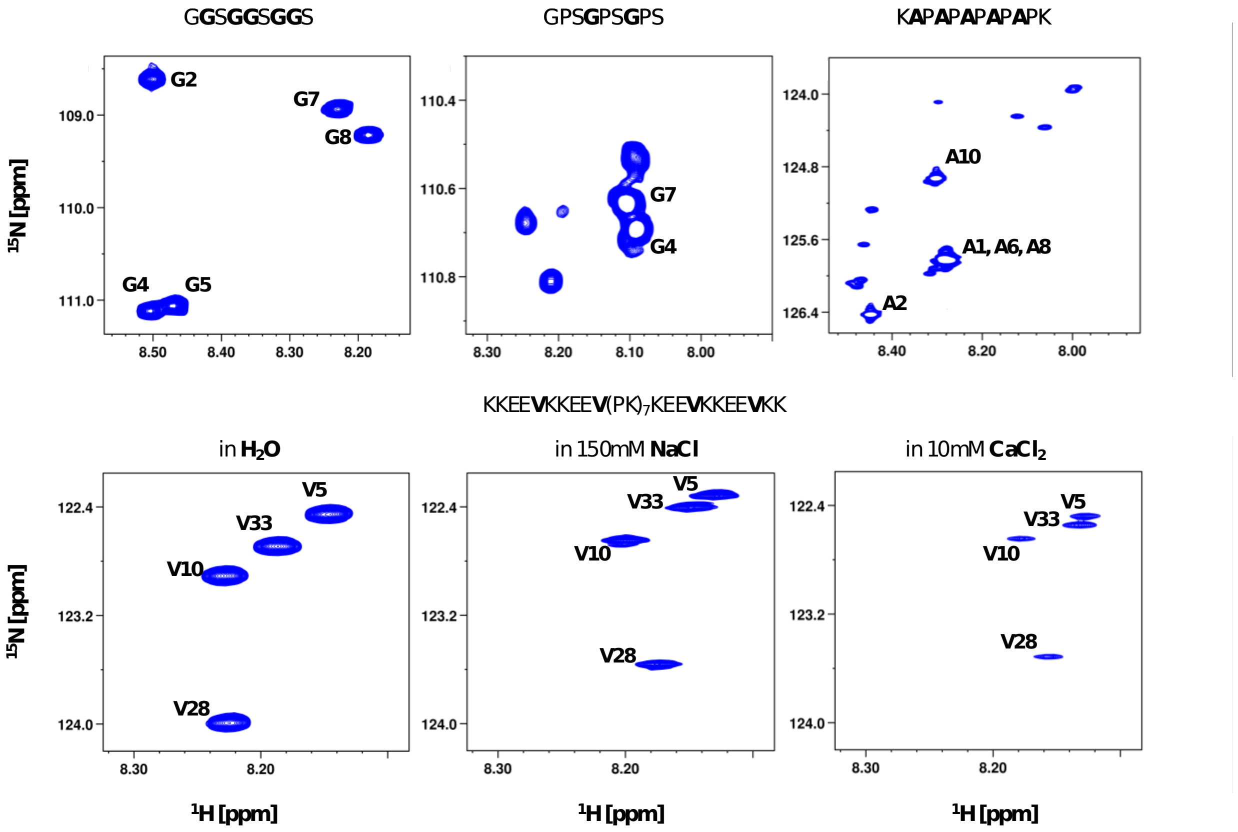

Figure S1: ^{1}H-^{15}N HSQC spectra for the short peptides (first row) and the charged peptide under different conditions (second row). Amino acid ^{1}5N labelled residues are highlighted in bold. Created with TopSpin 4.3.0.

Results from QEBSS simulations

$(GGS)_3$

Force field	Replica	R1 RMSD	R1 (%)	R2 RMSD	R2 (%)	hetNOE RMSD	hetNOE (%)	Sum (%)
AMBER99SB-DISP	replica04	0.053	100	0.24	110	0.48	110	320
AMBER99SB-DISP	replica01	0.063	120	0.24	110	0.43	100	330
AMBER99SB-DISP	replica03	0.071	130	0.25	110	0.45	100	350
AMBER99SB-DISP	replica05	0.068	130	0.28	130	0.47	110	360
AMBER99SB-DISP	replica02	0.071	130	0.27	120	0.48	110	370
DESAMBER	replica02	0.092	170	0.22	100	0.55	130	400
DESAMBER	replica03	0.088	160	0.27	120	0.53	120	410
DESAMBER	replica04	0.096	180	0.25	110	0.53	120	420
DESAMBER	replica05	0.092	170	0.28	130	0.53	120	420
DESAMBER	replica01	0.092	170	0.28	130	0.54	130	430
AMBER99SBWS	replica04	0.096	180	0.3	140	0.52	120	440
AMBER99SBWS	replica01	0.1	190	0.31	140	0.52	120	450
AMBER99SBWS	replica03	0.098	180	0.3	140	0.54	130	450
AMBER99SBWS	replica05	0.097	180	0.3	140	0.57	130	450
AMBER99SBWS	replica02	0.1	190	0.29	130	0.59	140	460
AMBER03WS	replica02	0.12	230	0.27	120	0.54	130	480
AMBER03WS	replica01	0.12	230	0.32	140	0.53	120	490
CHARMM36M	replica01	0.13	240	0.29	130	0.57	130	510
AMBER03WS	replica05	0.13	240	0.33	150	0.59	140	520
CHARMM36M	replica05	0.13	250	0.32	150	0.57	130	520
AMBER03WS	replica03	0.13	250	0.33	150	0.55	130	530
AMBER03WS	replica04	0.13	250	0.3	140	0.62	150	530
CHARMM36M	replica02	0.14	250	0.34	150	0.6	140	540
CHARMM36M	replica04	0.14	260	0.33	150	0.63	150	560
CHARMM36M	replica03	0.14	270	0.32	150	0.65	150	570

Figure S2: $(GGS)_3$: RMSD and percentage difference to experimental values for relaxation rates R1, R2 and hetNOE. Ranking numbers are determined by their fraction of the lowest RMSD value for each spin relaxation rate. Simulations deviating less than 50% from the lowest RMSD for all spin relaxation rates are selected as best and highlighted in bold

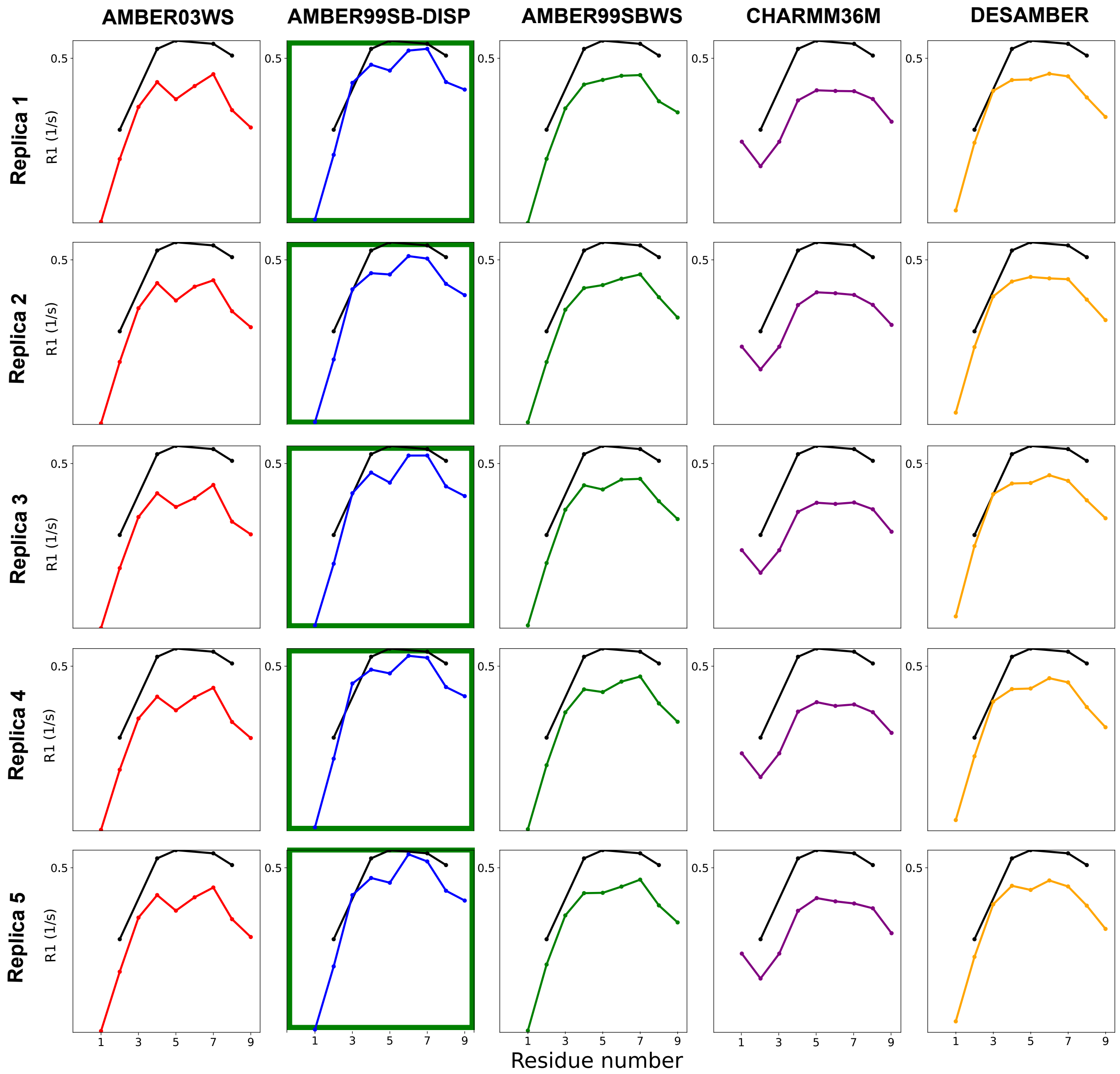

Figure S3: R1 relaxation rates from 25 simulations of the five replicas and force fields for the $(GGS)_3$ peptide (colored lines), plotted against experimental R1 rates (black lines).

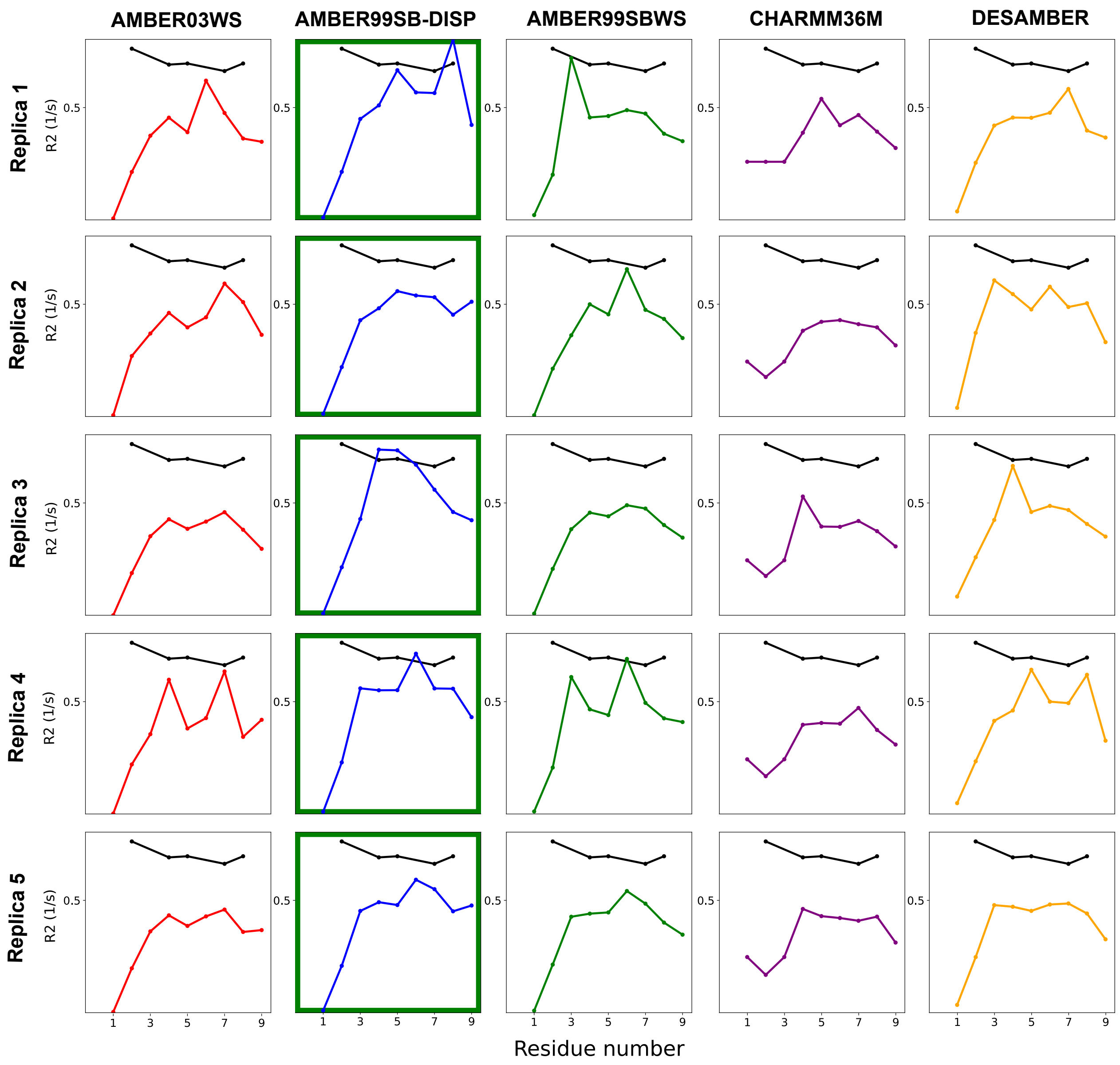

Figure S4: R2 relaxation rates from 25 simulations of the five replicas and force fields for the $(GGS)_3$ peptide (colored lines), plotted against experimental R2 rates (black lines).

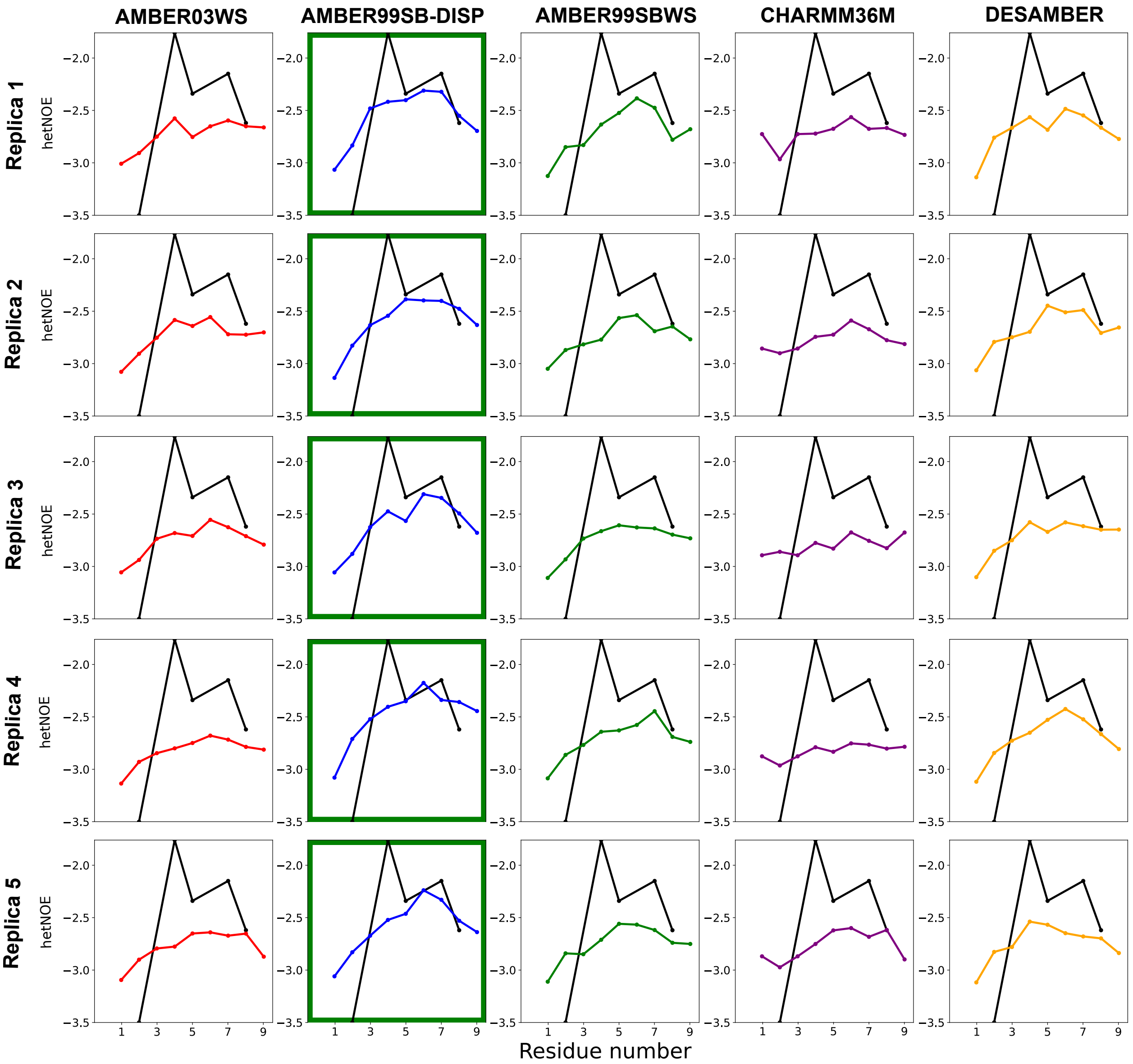

Figure S5: HetNoe relaxation rates from 25 simulations of the five replicas and force fields for the $(GGS)_3$ peptide (colored lines), plotted against experimental hetNoe rates (black lines).

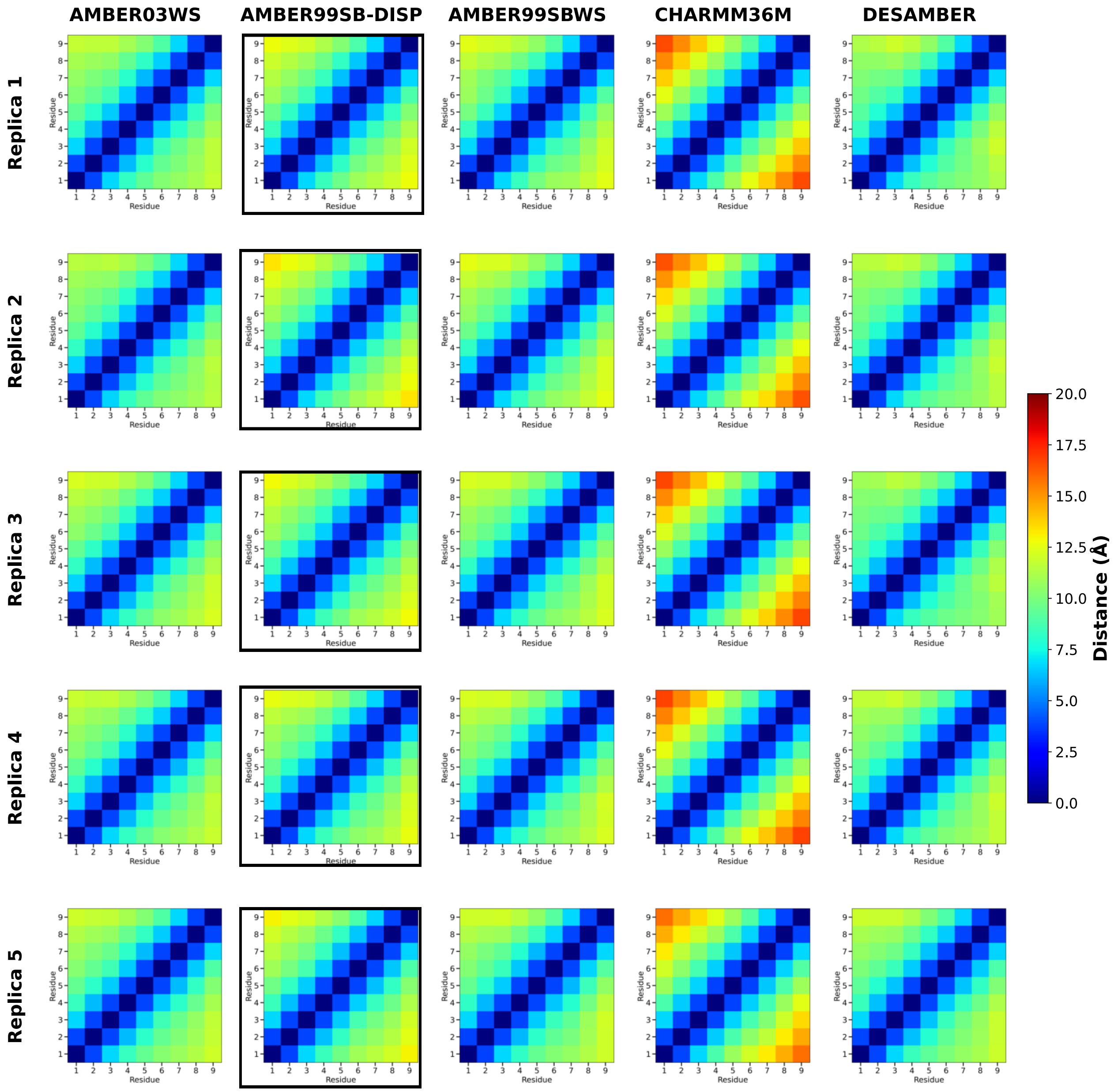

Figure S6: Distance maps for the $(GGS)_3$ peptide.

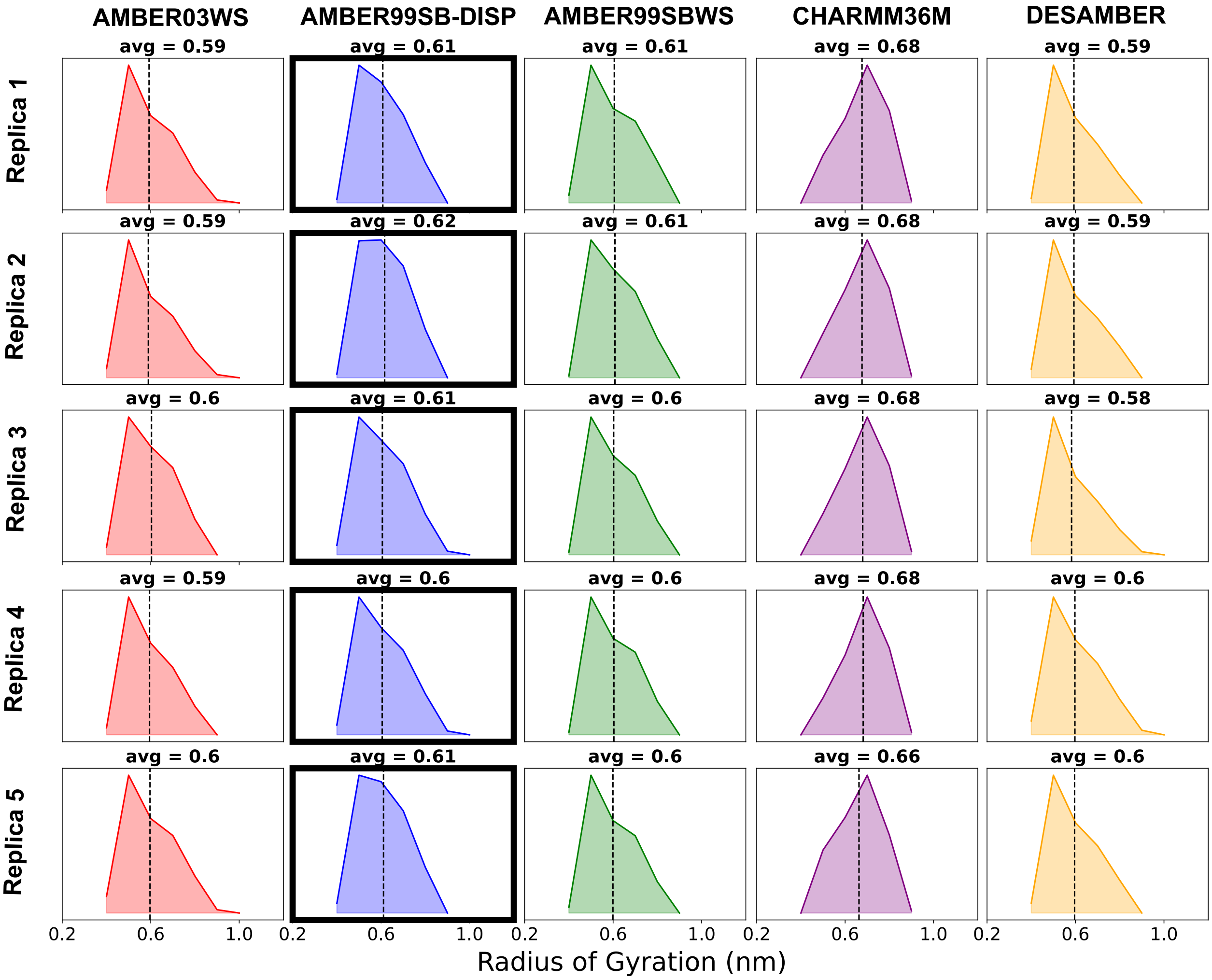

Figure S7: Radius of gyration distribution generated from five different initial structures and force fields for the $(GGS)_3$ peptide.

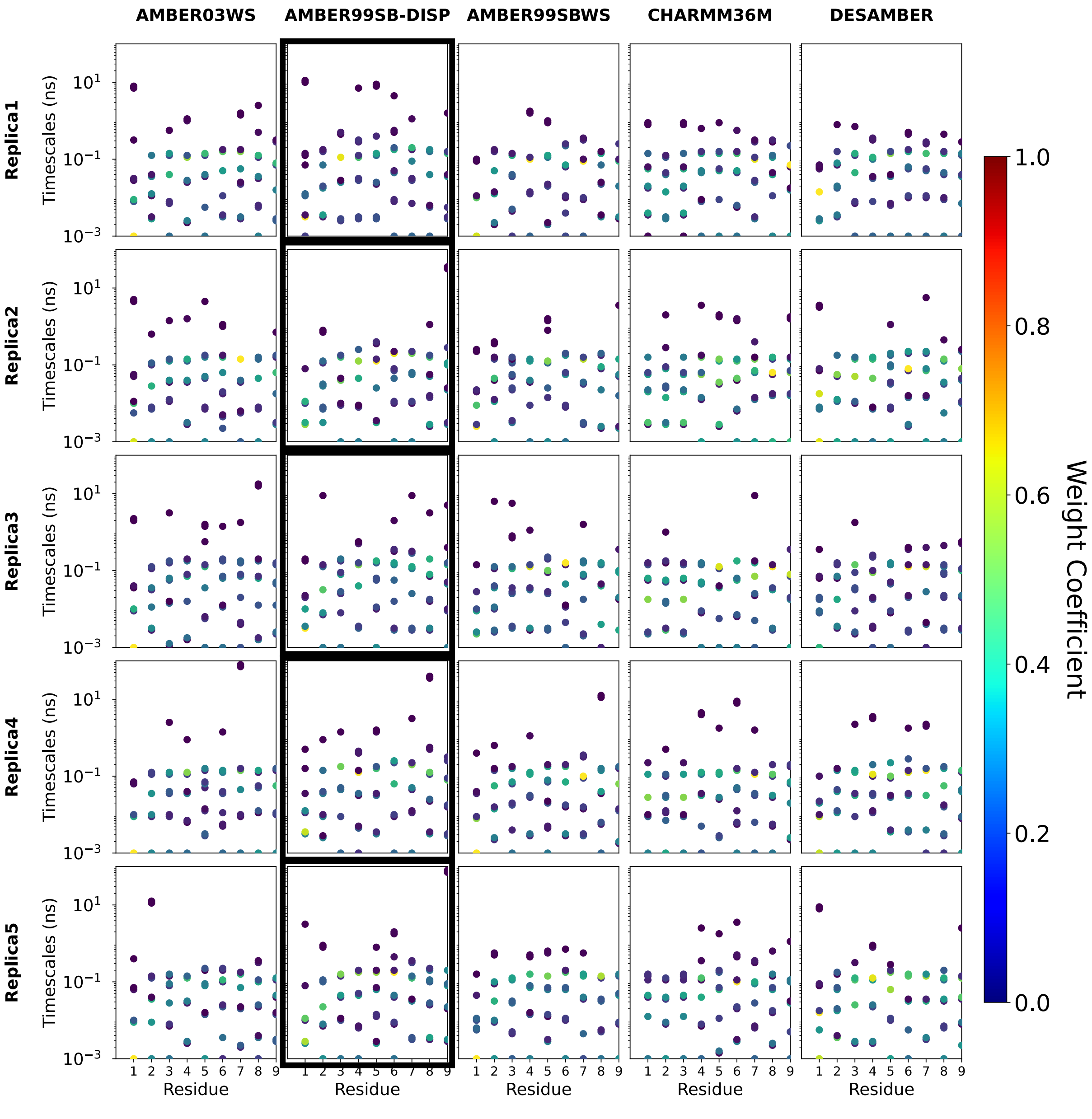

Figure S8: Timescales of backbone N-H bonds in $(GGS)_3$.

$(GPS)_3$

Force field	Replica	R1 RMSD	R1 (%)	R2 RMSD	R2 (%)	hetNOE RMSD	hetNOE (%)	Sum (%)
DESAMBER	replica03	0.012	100	0.047	180	0.21	190	470
AMBER99SB-DISP	replica04	0.037	320	0.027	100	0.21	190	610
DESAMBER	replica01	0.02	170	0.056	210	0.28	260	640
AMBER99SB-DISP	replica02	0.043	370	0.054	200	0.14	130	700
AMBER99SB-DISP	replica03	0.026	220	0.11	430	0.13	120	770
DESAMBER	replica05	0.027	230	0.076	280	0.35	320	830
DESAMBER	replica02	0.023	200	0.096	360	0.34	310	870
AMBER99SB-DISP	replica01	0.043	370	0.12	450	0.11	100	920
DESAMBER	replica04	0.022	190	0.089	330	0.45	410	940
AMBER99SBWS	replica02	0.031	260	0.085	320	0.6	550	1100
AMBER99SBWS	replica05	0.046	400	0.07	260	0.46	420	1100
AMBER99SB-DISP	replica05	0.075	650	0.11	410	0.11	100	1200
AMBER99SBWS	replica01	0.043	370	0.12	440	0.5	460	1300
AMBER03WS	replica02	0.068	580	0.11	430	0.44	400	1400
AMBER99SBWS	replica04	0.058	500	0.11	410	0.56	510	1400
AMBER03WS	replica05	0.064	550	0.12	460	0.44	410	1400
AMBER03WS	replica01	0.062	540	0.14	510	0.44	410	1500
AMBER99SBWS	replica03	0.059	510	0.14	520	0.49	450	1500
AMBER03WS	replica04	0.068	580	0.14	520	0.58	530	1600
AMBER03WS	replica03	0.082	710	0.12	460	0.59	550	1700
CHARMM36M	replica02	0.12	1000	0.11	410	0.87	800	2200
CHARMM36M	replica03	0.1	870	0.18	660	0.78	720	2200
CHARMM36M	replica01	0.11	940	0.2	750	0.9	820	2500
CHARMM36M	replica04	0.12	1100	0.15	550	0.9	830	2500
CHARMM36M	replica05	0.11	970	0.2	750	0.92	840	2600

Figure S9: $(GPS)_3$: RMSD and percentage difference to experiment values for relaxation rates R1, R2 and hetNOE. Ranking numbers are determined by their fraction of the lowest RMSD value for each spin relaxation rate. Due to insufficient number of experimental data points, comparison numbers are very large, surpassing the 150. Thus, in this case the simulation exhibiting comparison numbers close to 150 for R1 and R2 rates and one of the smallest comparison numbers for hetNOE, at the same time, was selected.

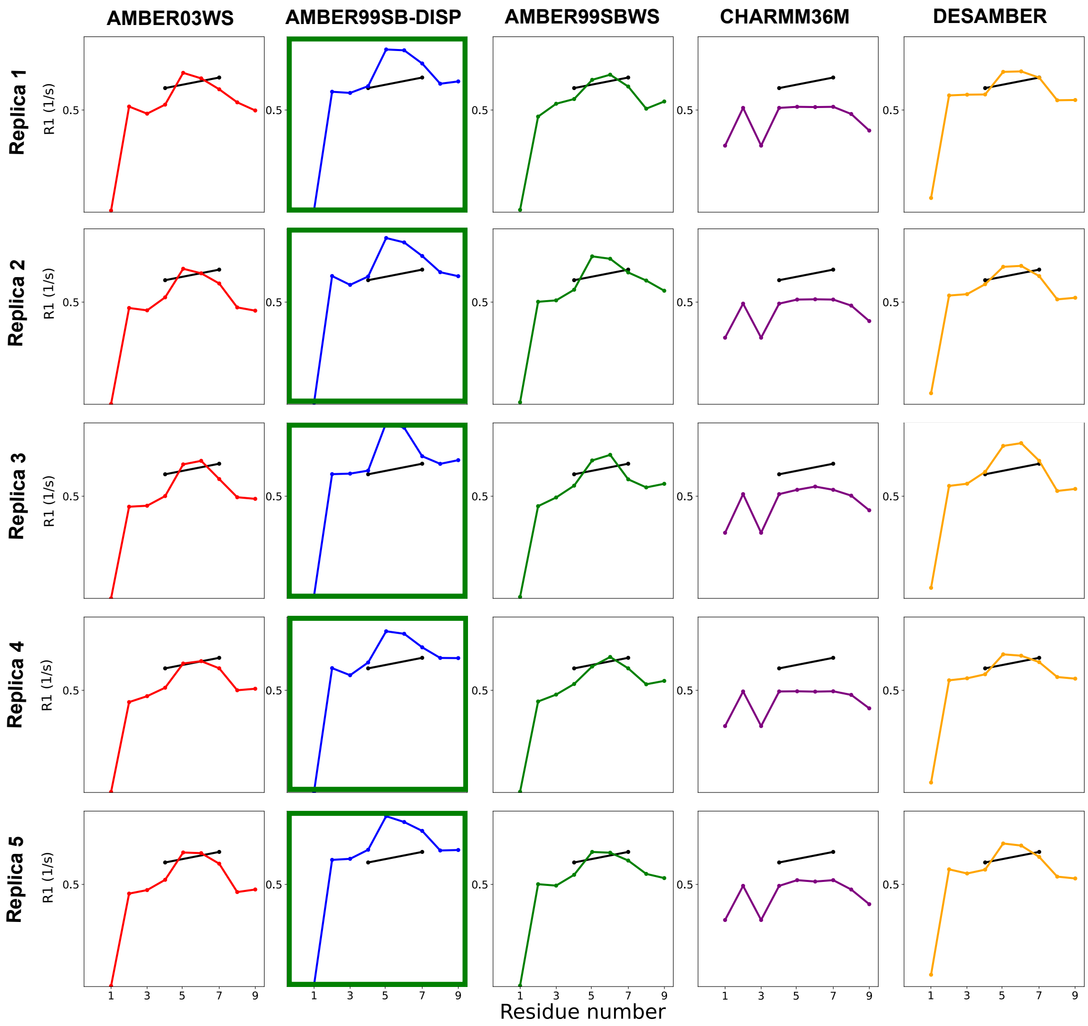

Figure S10: R1 relaxation rate plots for 25 simulations of different models and force fields of $(GPS)_3$ peptide.

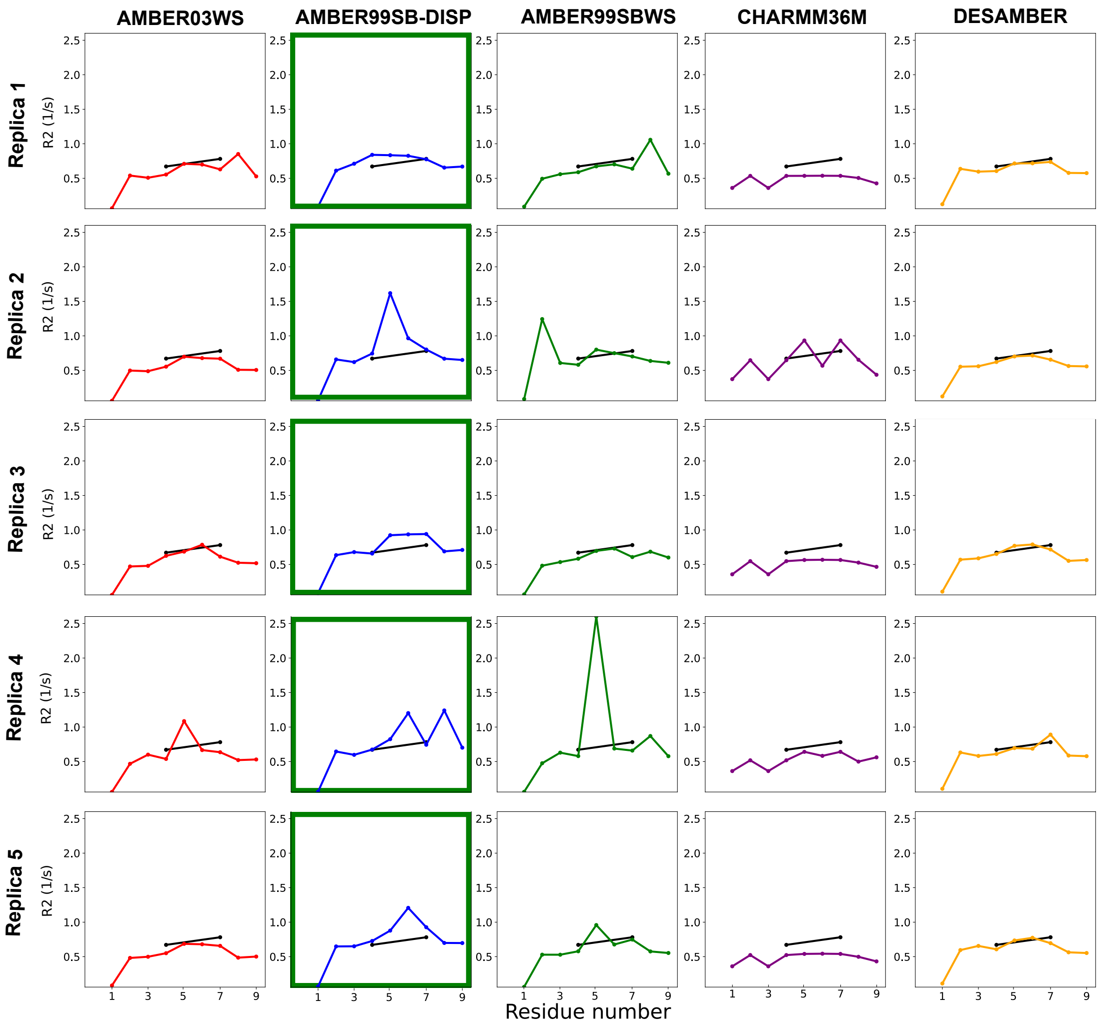

Figure S11: R2 relaxation rate plots for 25 simulations of different models and force fields of $(GPS)_3$ peptide.

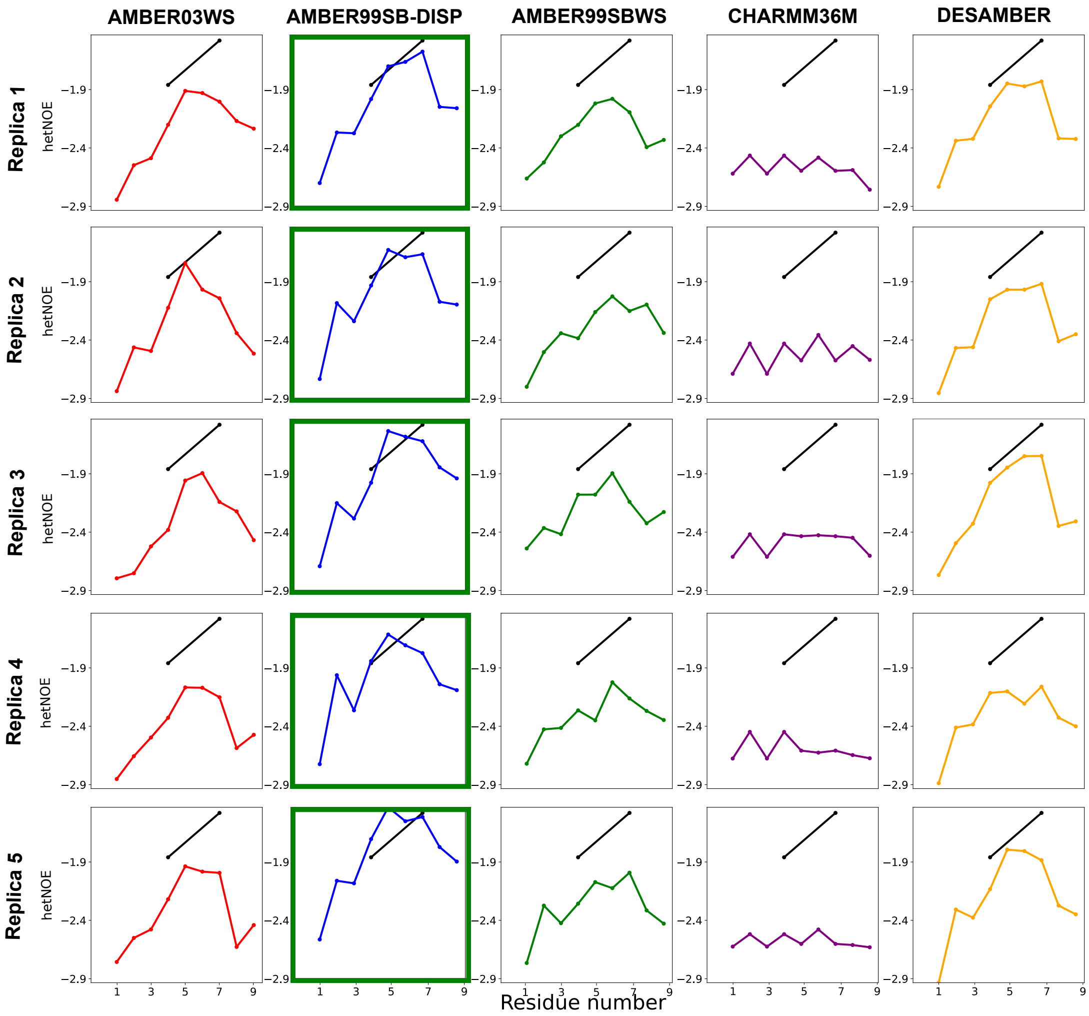

Figure S12: HetNoe relaxation time plots for 25 simulations of different models and force fields of $(GPS)_3$ peptide.

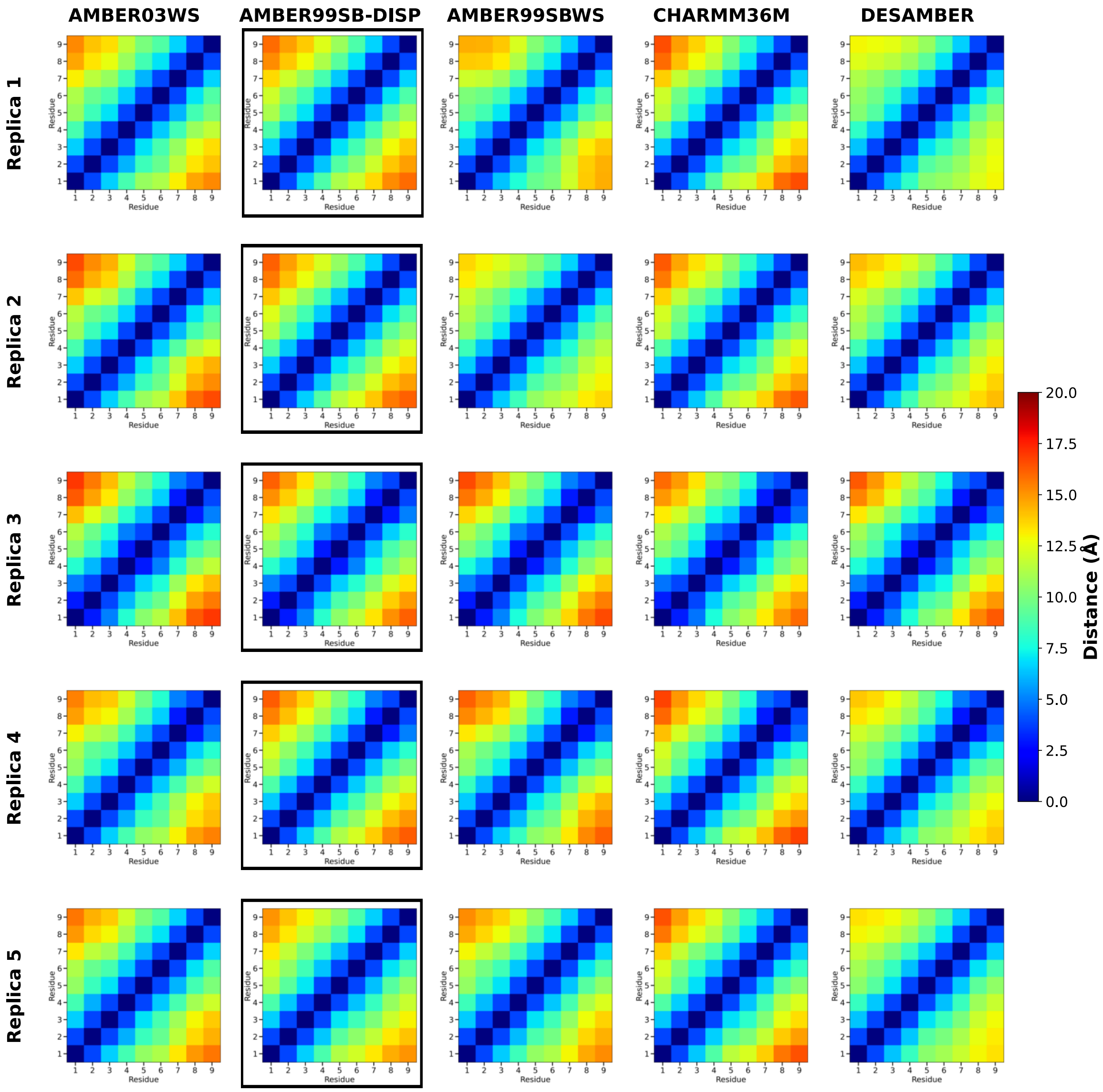

Figure S13: Distance maps for the $(GPS)_3$ peptide.

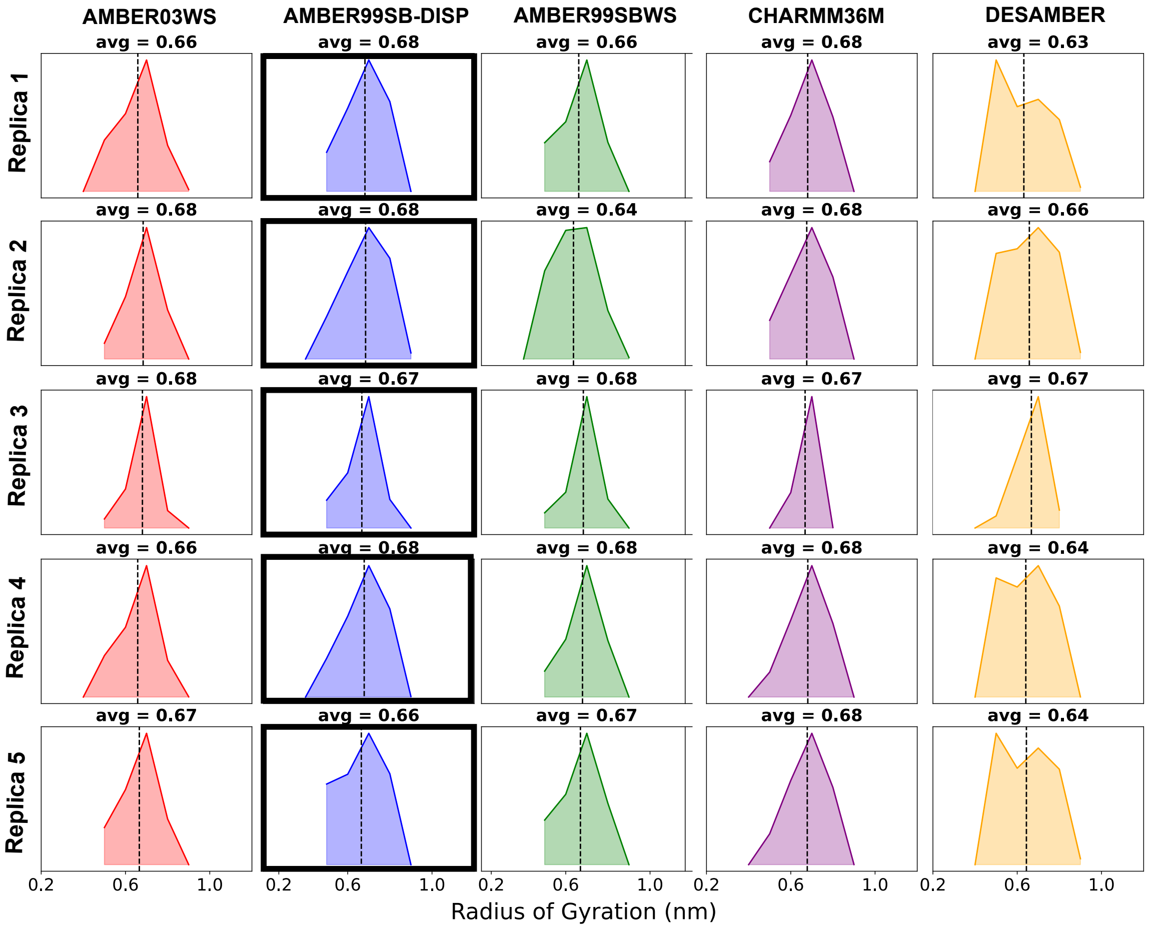

Figure S14: Radius of gyration distribution generated from five different initial structures and force fields for the $(GPS)_3$ peptide.

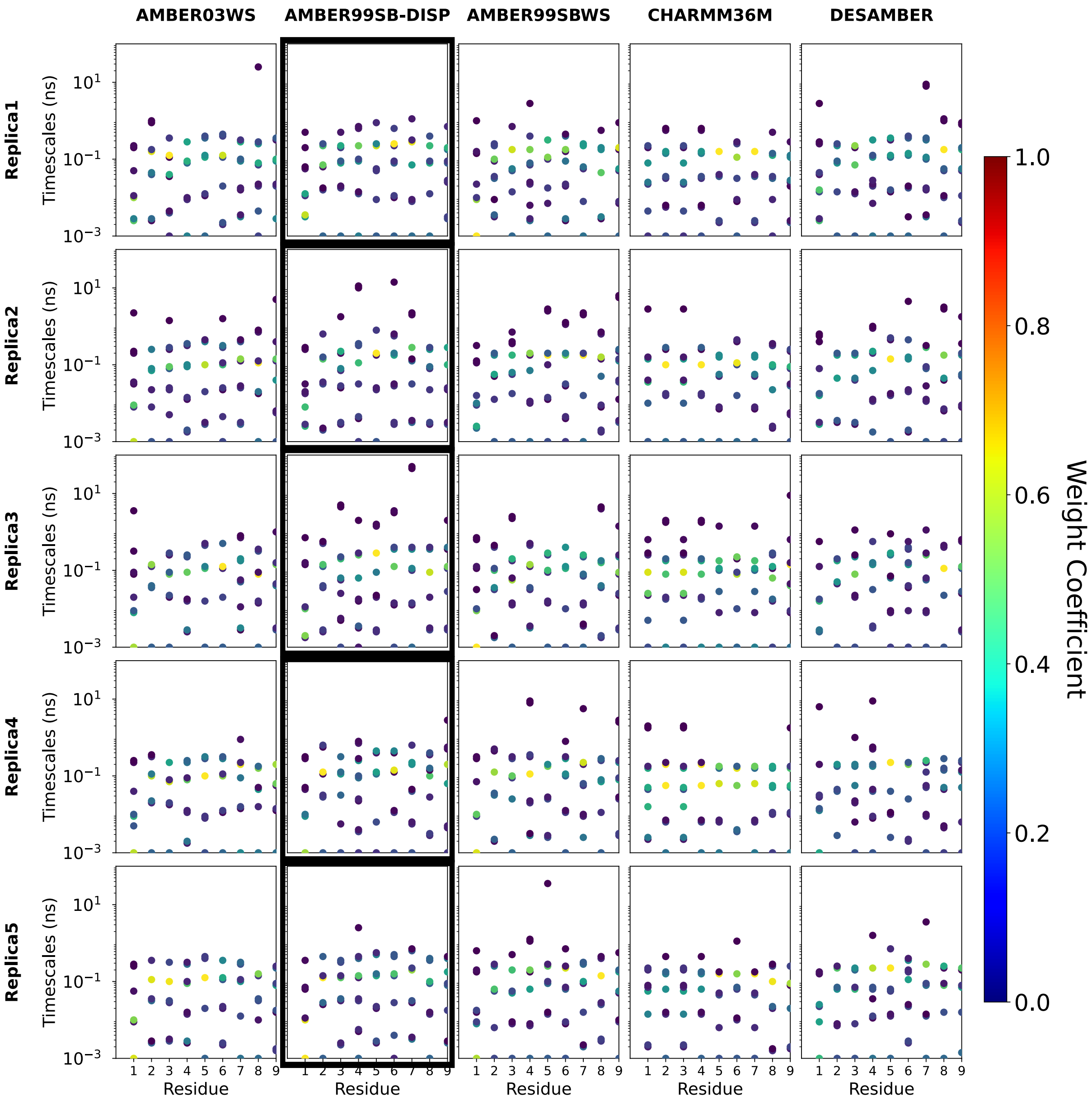

Figure S15: Timescales of backbone N-H bonds in $(GPS)_3$.

$K(AP)_5K$

Force field	Replica	R1 RMSD	R1 (%)	R2 RMSD	R2 (%)	hetNOE RMSD	hetNOE (%)	Sum (%)
DESAMBER	replica02	0.076	120	0.046	100	0.41	120	340
DESAMBER	replica04	0.1	160	0.08	170	0.49	140	470
DESAMBER	replica01	0.098	150	0.099	210	0.4	120	480
AMBER03WS	replica05	0.14	230	0.065	140	0.8	230	600
AMBER99SB-DISP	replica05	0.1	160	0.2	440	0.39	110	700
AMBER99SBWS	replica01	0.12	190	0.16	360	0.64	180	730
AMBER99SB-DISP	replica03	0.1	160	0.21	450	0.45	130	740
AMBER03WS	replica01	0.12	180	0.17	380	0.71	210	760
AMBER99SB-DISP	replica04	0.13	210	0.2	440	0.48	140	790
AMBER03WS	replica02	0.14	220	0.16	350	0.84	240	820
AMBER99SBWS	replica04	0.17	260	0.15	330	0.83	240	830
AMBER99SBWS	replica05	0.16	250	0.16	340	0.81	230	830
AMBER03WS	replica03	0.16	250	0.19	410	0.74	210	880
AMBER99SBWS	replica02	0.16	250	0.19	400	0.79	230	890
AMBER99SBWS	replica03	0.16	250	0.19	420	0.87	250	920
DESAMBER	replica05	0.17	260	0.37	790	0.43	120	1200
AMBER03WS	replica04	0.18	280	0.34	730	0.92	270	1300
DESAMBER	replica03	0.064	100	0.64	1400	0.48	140	1600
CHARMM36M	replica03	0.38	600	0.37	800	1.1	320	1700
CHARMM36M	replica05	0.34	540	0.46	1000	1.1	320	1900
CHARMM36M	replica02	0.36	560	0.5	1100	1.1	310	2000
CHARMM36M	replica04	0.37	570	0.53	1100	1.1	320	2000
CHARMM36M	replica01	0.37	580	0.51	1100	1.4	400	2100
AMBER99SB-DISP	replica01	0.17	260	0.94	2000	0.35	100	2400
AMBER99SB-DISP	replica02	0.12	190	0.99	2200	0.46	130	2500

Figure S16: $K(AP)_5K$: RMSD and percentage difference to experiment values for relaxation rates R1, R2 and hetNOE. Ranking numbers are determined by their fraction of the lowest RMSD value for each spin relaxation rate. Simulations deviating less than 50 from the lowest RMSD for all spin relaxation rates are selected as best and highlighted in bold.

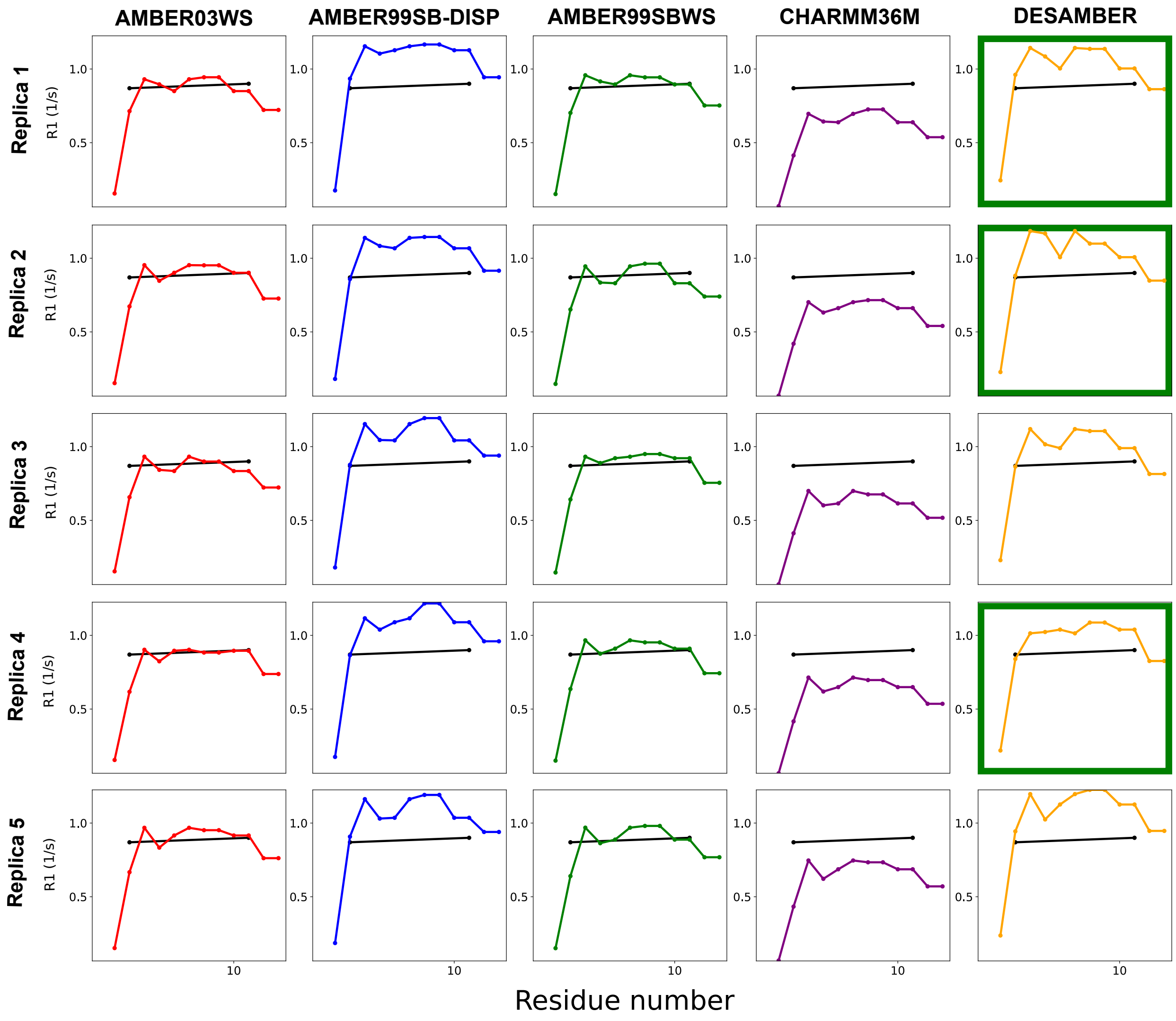

Figure S17: R1 relaxation rate plots for 25 simulations of different models and force fields of $K(AP)_5K$ peptide.

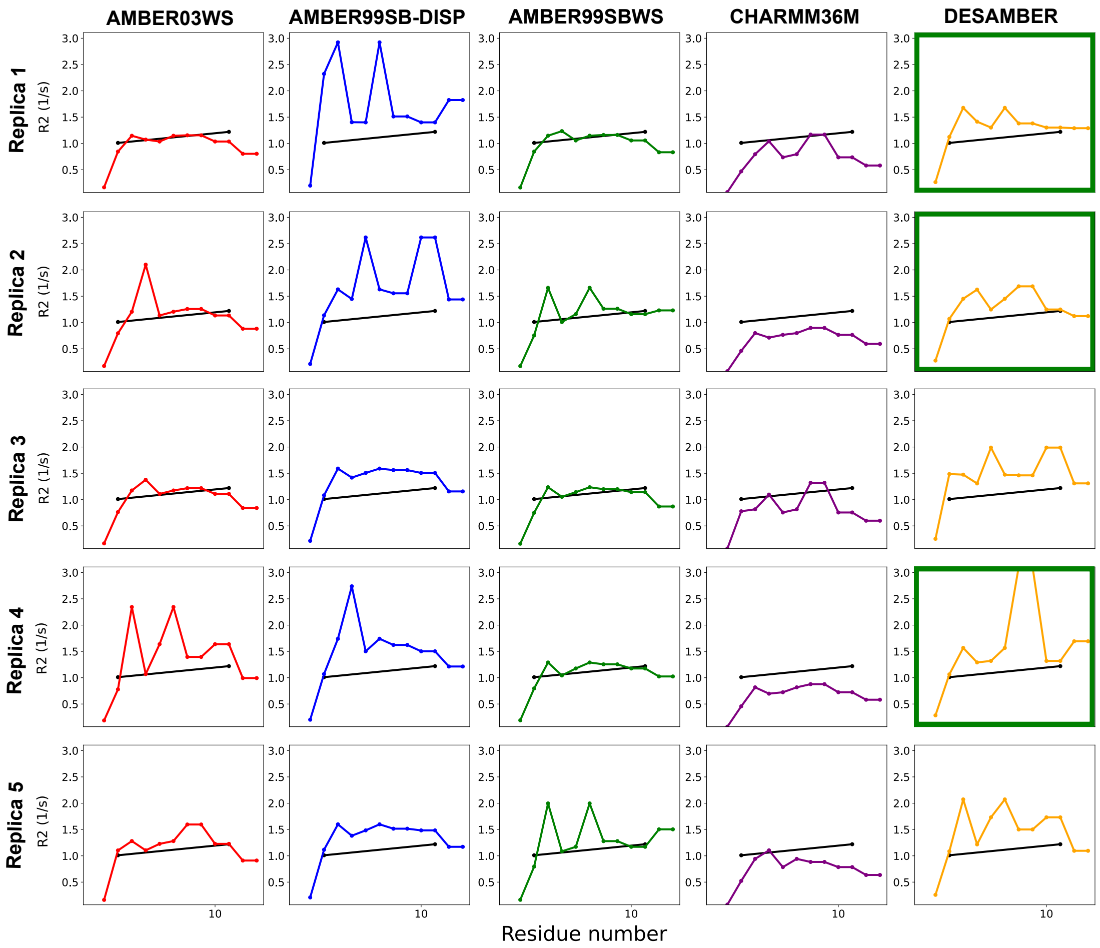

Figure S18: R2 relaxation rate plots for 25 simulations of different models and force fields of K(AP)$_5$K peptide.

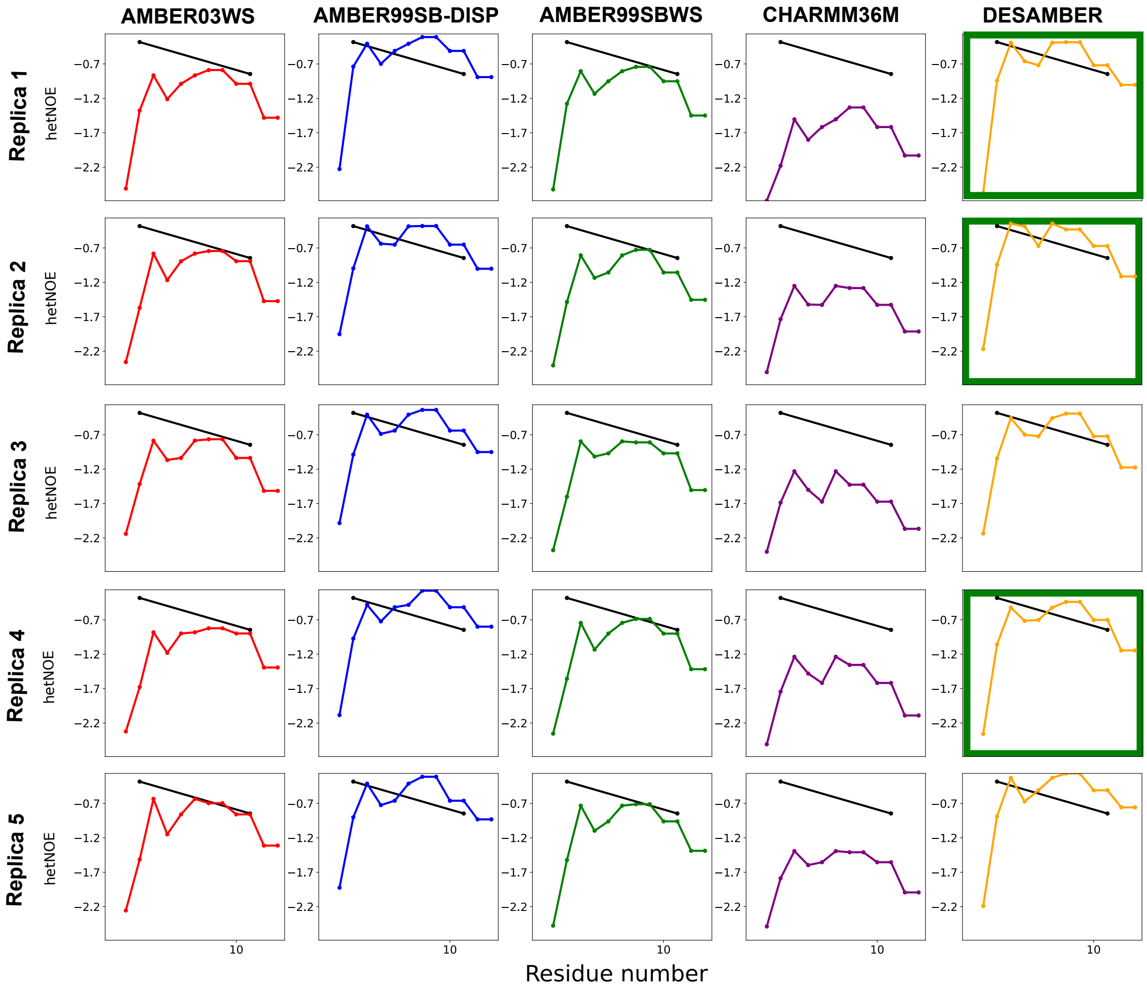

Figure S19: HetNoe relaxation time plots for 25 simulations of different models and force fields of $K(AP)_5K$ peptide.

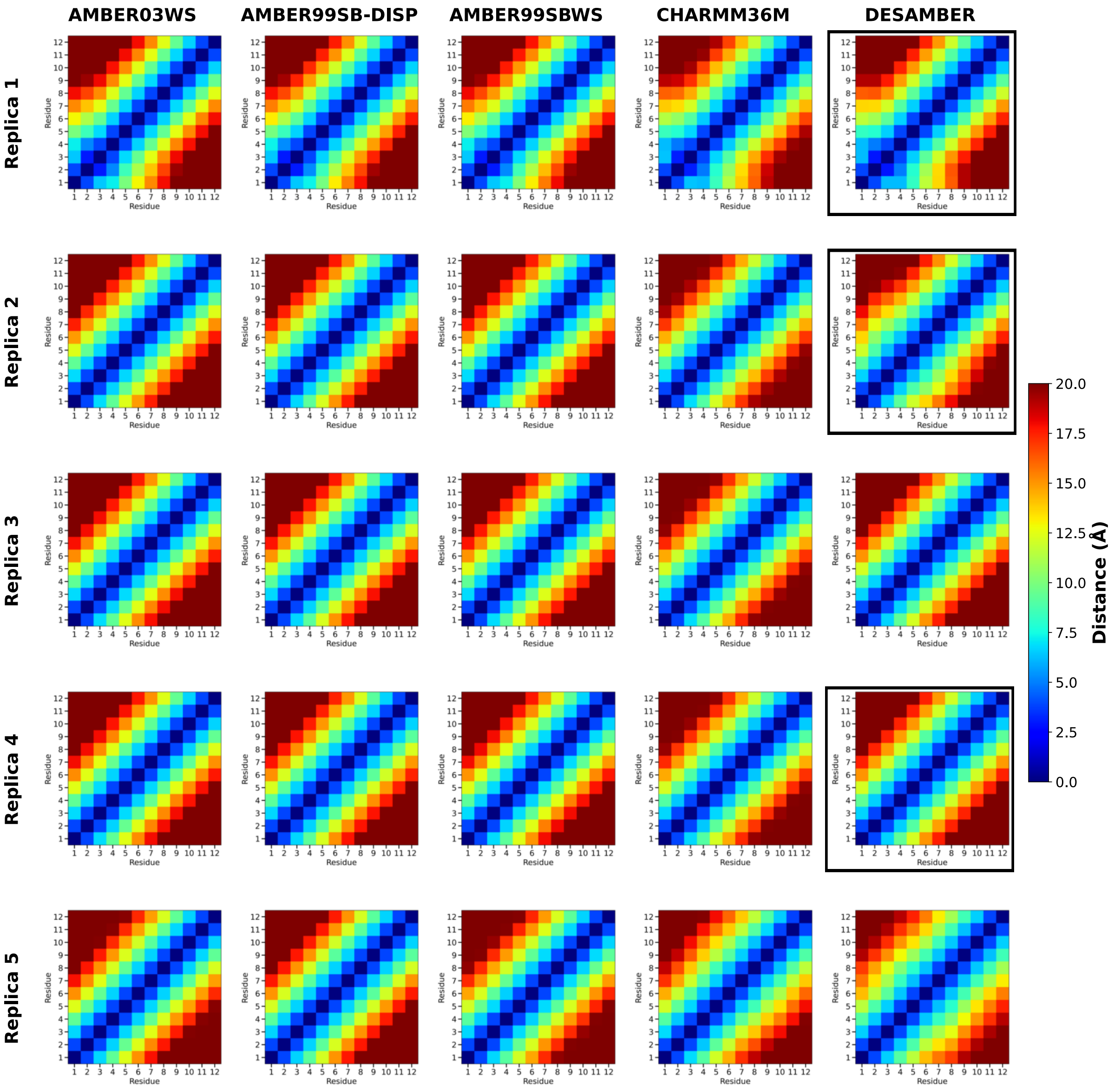

Figure S21: Distance maps for the $K(AP)_5K$ peptide.

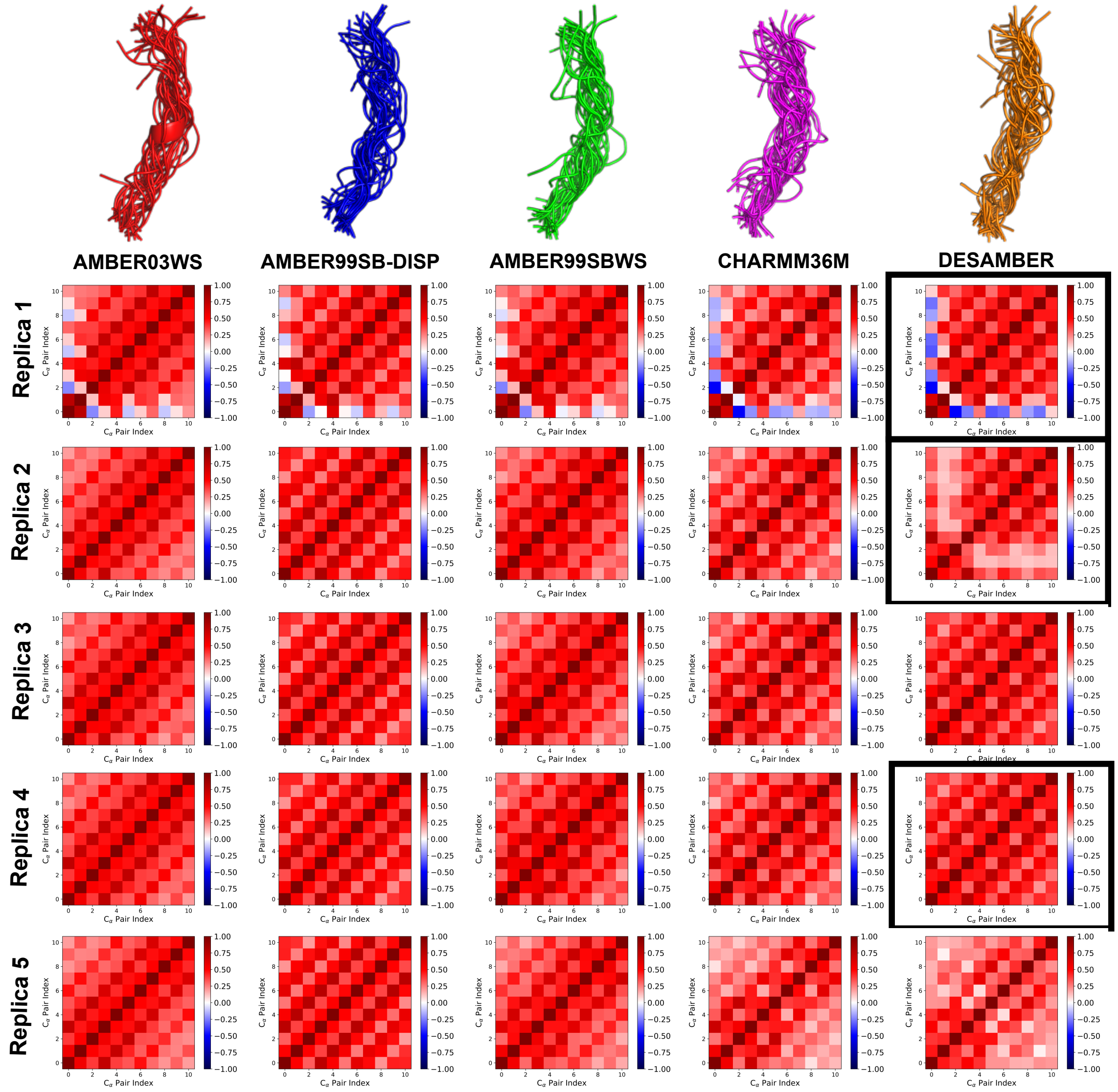

Figure S20: Consecutive Ca vector correlation maps generated from five different initial structures and force fields, along with representative snapshots for the K(AP)$_5$K peptide. Positive and negative values indicate positive and negative correlation respectively. The snapshots represent 5 overlayed frames from each force field for the same initial structure model of the peptide. The selected simulations from the quality evaluation are highlighted with the square.

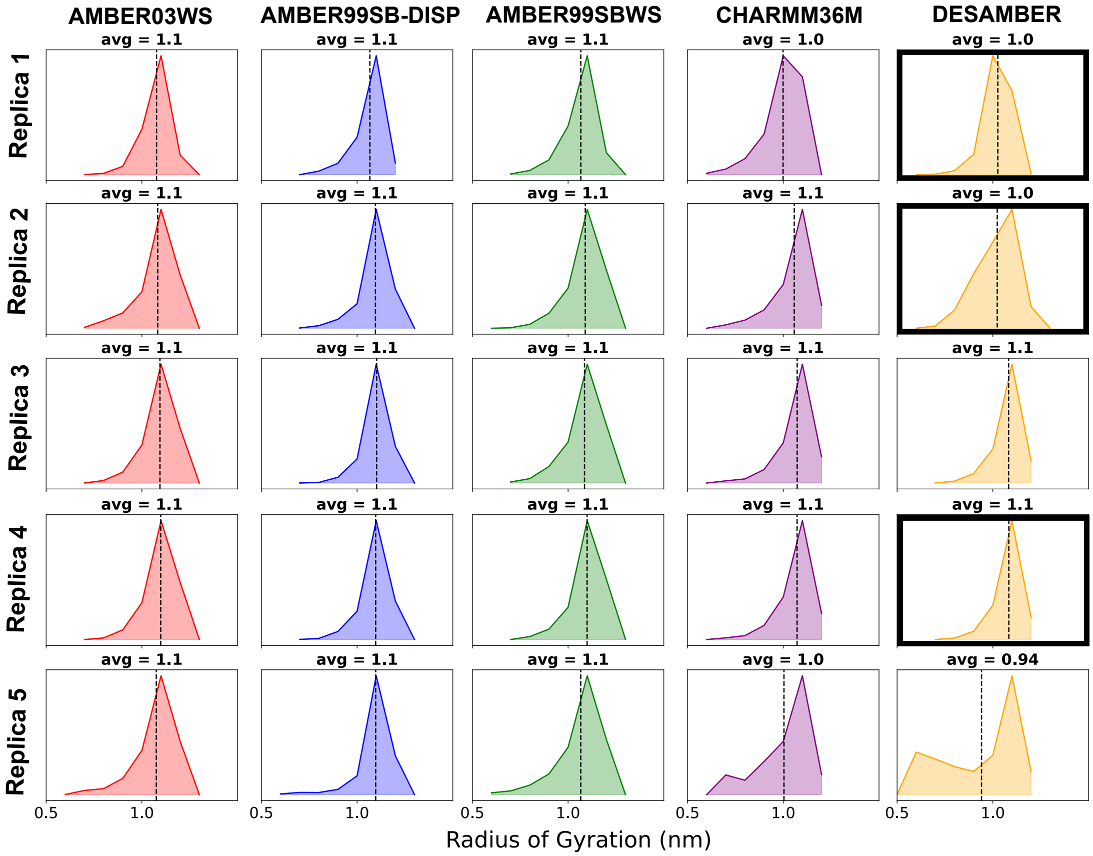

Figure S22: Radius of gyration distribution generated from five different initial structures and force fields for the $K(AP)_5K$ peptide.

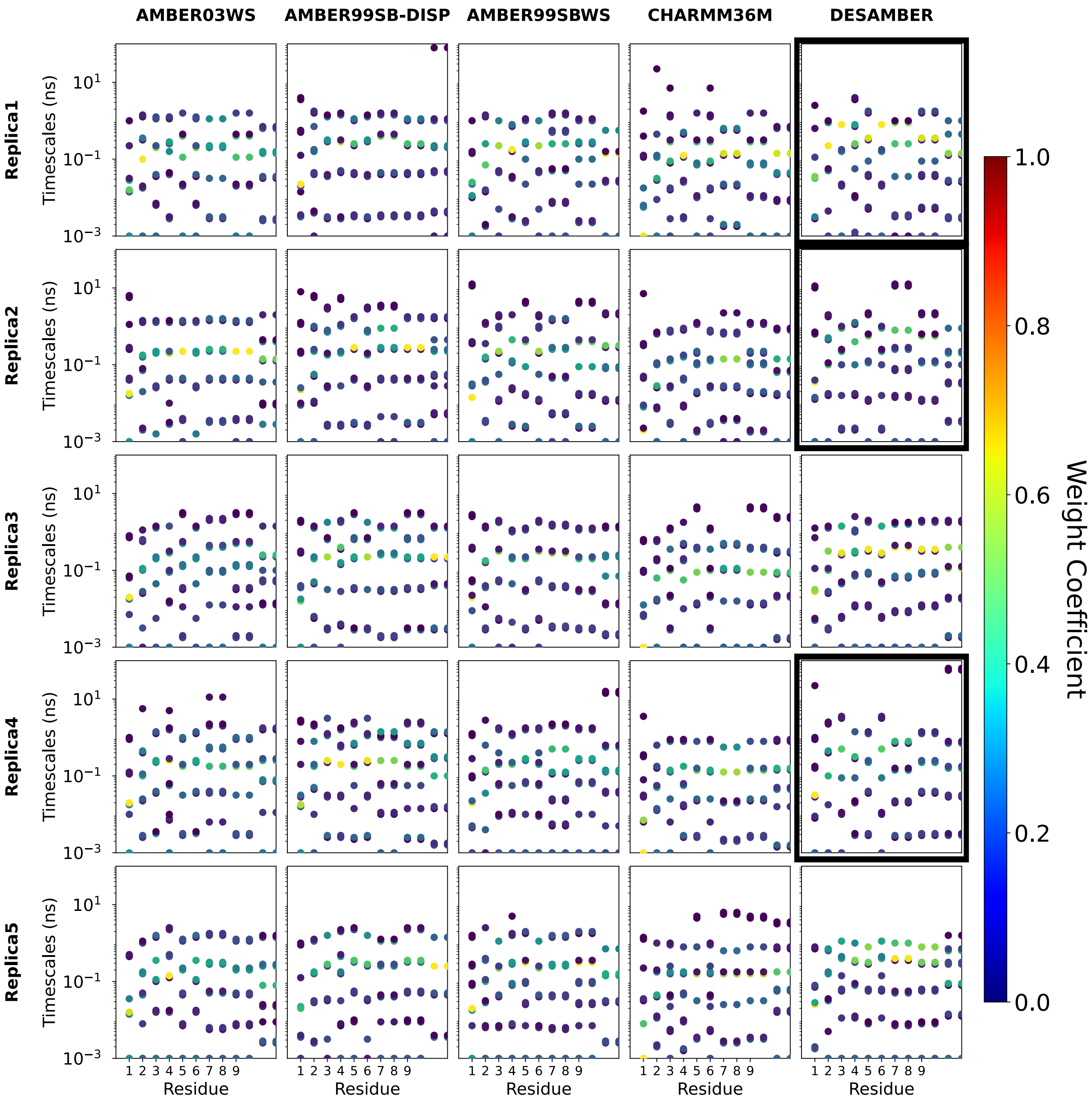

Figure S23: Timescales of of backbone N-H bonds in $K(AP)_5K$.

Charged sequence: KKEEVKKEEV(PK)$_7$KEEVKKEEVKK

Force field	Replica	R1 RMSD	R1 (%)	R2 RMSD	R2 (%)	hetNOE RMSD	hetNOE (%)	Sum (%)
CHARMM36M	replica01	0.064	140	0.37	170	0.23	120	430
AMBER03WS	replica04	0.08	170	0.28	130	0.25	130	430
AMBER03WS	replica01	0.079	170	0.31	140	0.25	130	440
AMBER03WS	replica02	0.094	200	0.22	100	0.26	140	440
CHARMM36M	replica04	0.091	200	0.42	190	0.37	190	580
DESAMBER	replica04	0.12	250	0.48	220	0.25	130	600
AMBER99SBWS	replica05	0.17	370	0.35	160	0.25	130	660
AMBER99SB-DISP	replica04	0.19	420	0.39	180	0.28	140	740
CHARMM36M	replica02	0.11	250	0.59	270	0.46	240	760
AMBER99SBWS	replica01	0.16	340	0.79	360	0.19	100	800
CHARMM36M	replica05	0.08	170	1.0	470	0.38	200	840
AMBER03WS	replica05	0.046	100	1.4	660	0.28	150	900
DESAMBER	replica02	0.15	320	1.0	480	0.26	130	940
AMBER99SBWS	replica03	0.26	550	1.0	460	0.21	110	1100
AMBER99SB-DISP	replica05	0.18	380	1.2	550	0.24	130	1100
AMBER99SBWS	replica04	0.26	570	1.0	460	0.24	130	1200
AMBER99SBWS	replica02	0.21	460	1.7	790	0.22	110	1400
CHARMM36M	replica03	0.074	160	2.7	1200	0.32	170	1500
DESAMBER	replica05	0.19	420	2.1	960	0.28	150	1500
AMBER03WS	replica03	0.097	210	2.7	1200	0.23	120	1600
DESAMBER	replica03	0.19	400	2.8	1300	0.27	140	1800
AMBER99SB-DISP	replica01	0.2	430	3.3	1500	0.25	130	2100
DESAMBER	replica01	0.15	330	3.6	1600	0.24	130	2100
AMBER99SB-DISP	replica02	0.17	360	3.8	1700	0.21	110	2200
AMBER99SB-DISP	replica03	0.21	460	3.8	1700	0.28	150	2300

Figure S24: charged peptide: RMSD and percentage difference to experiment values for relaxation rates R1, R2 and hetNOE. Ranking numbers are determined by their fraction of the lowest RMSD value for each spin relaxation rate. Simulations deviating less than 50 from the lowest RMSD for all spin relaxation rates are selected as best and highlighted in bold

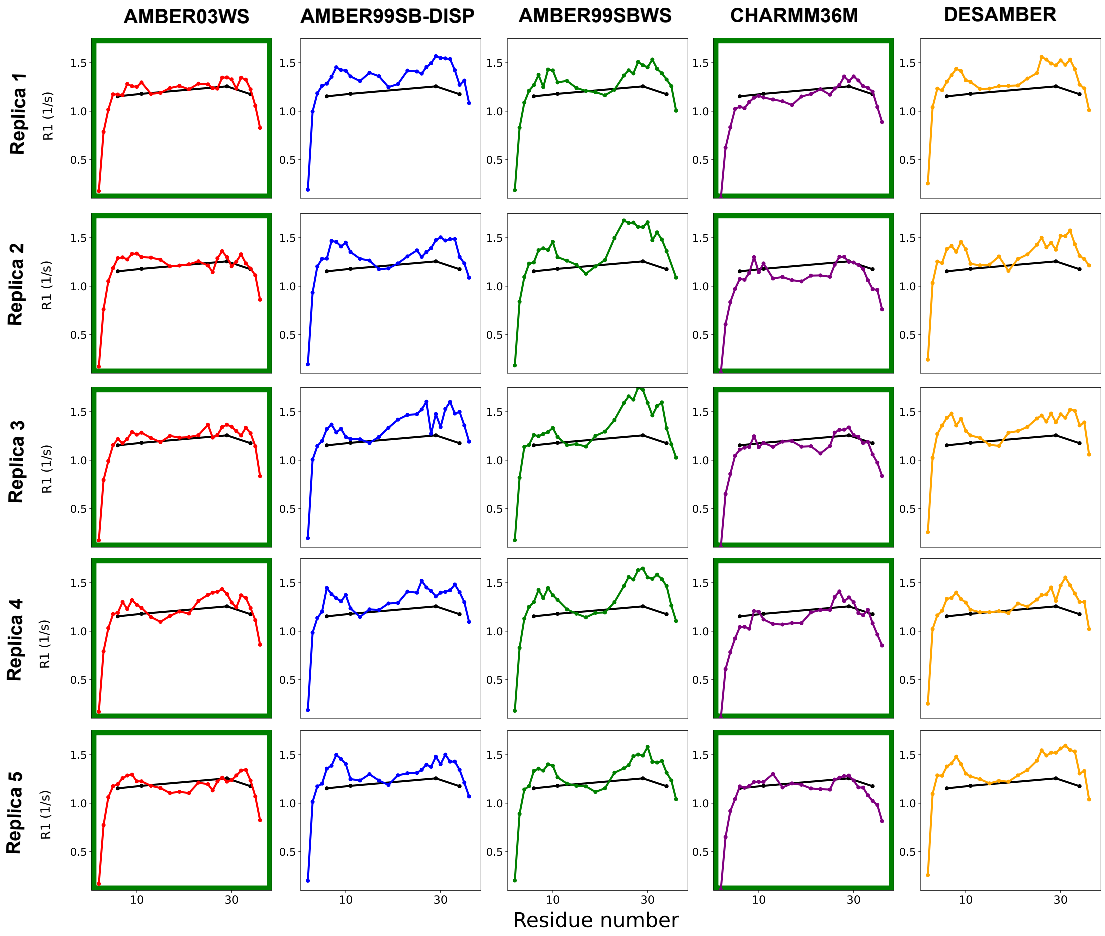

Figure S25: R1 relaxation rate plots for 25 simulations of different models and force fields of charged peptide.

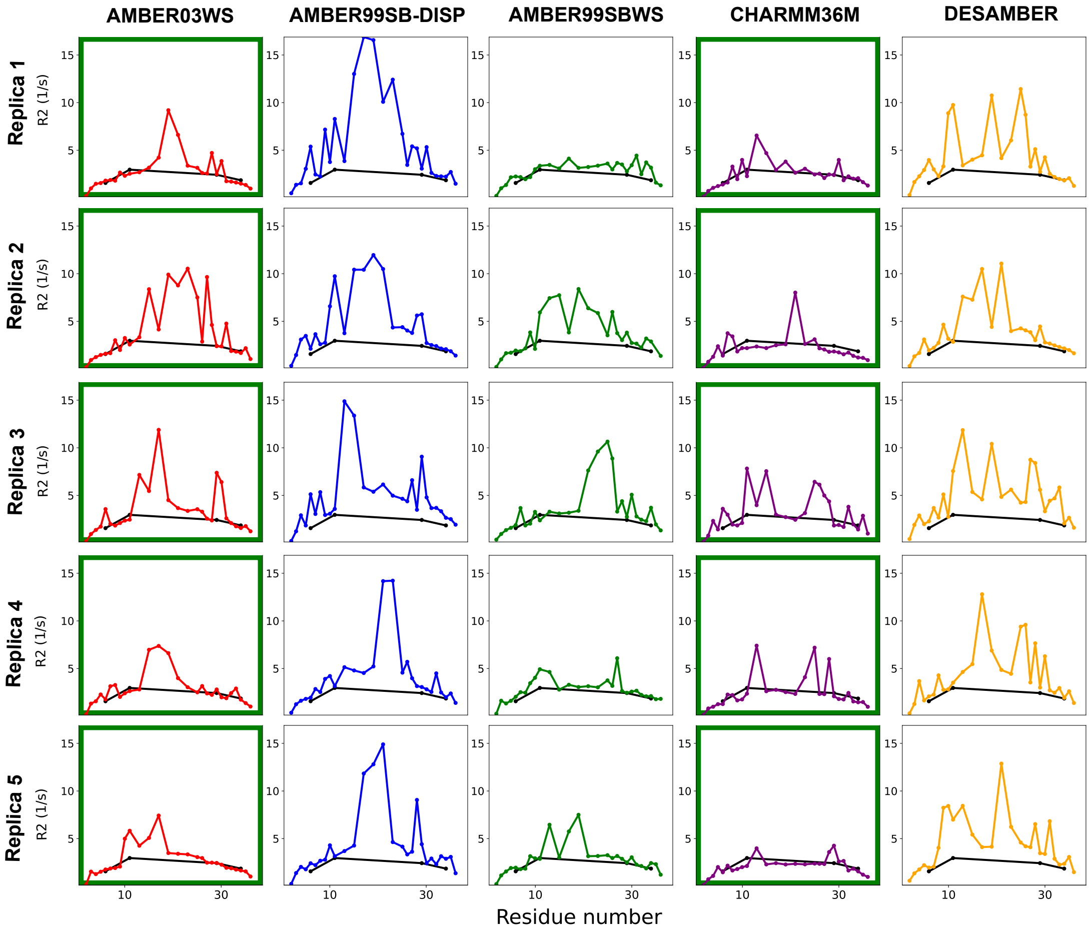

Figure S26: R2 relaxation rate plots for 25 simulations of different models and force fields of charged peptide.

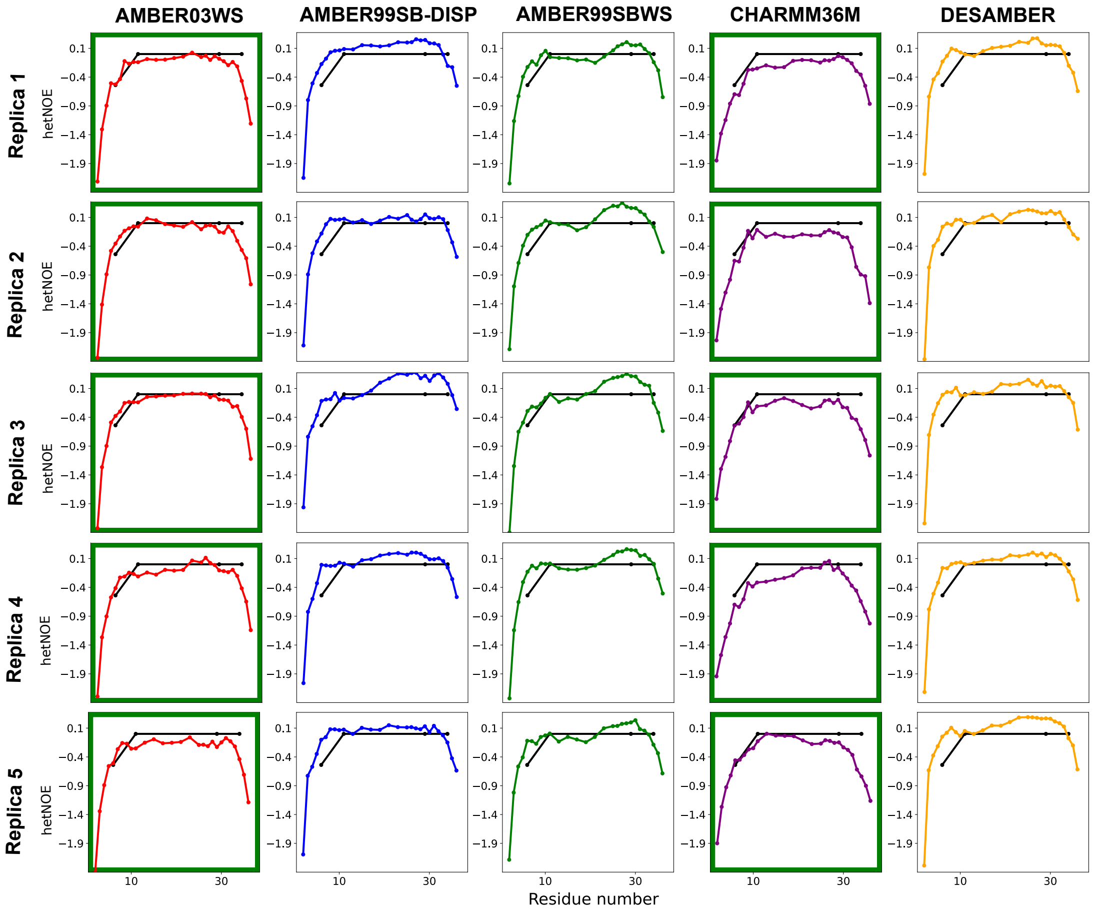

Figure S27: HetNoe relaxation time plots for 25 simulations of different models and force fields of charged peptide.

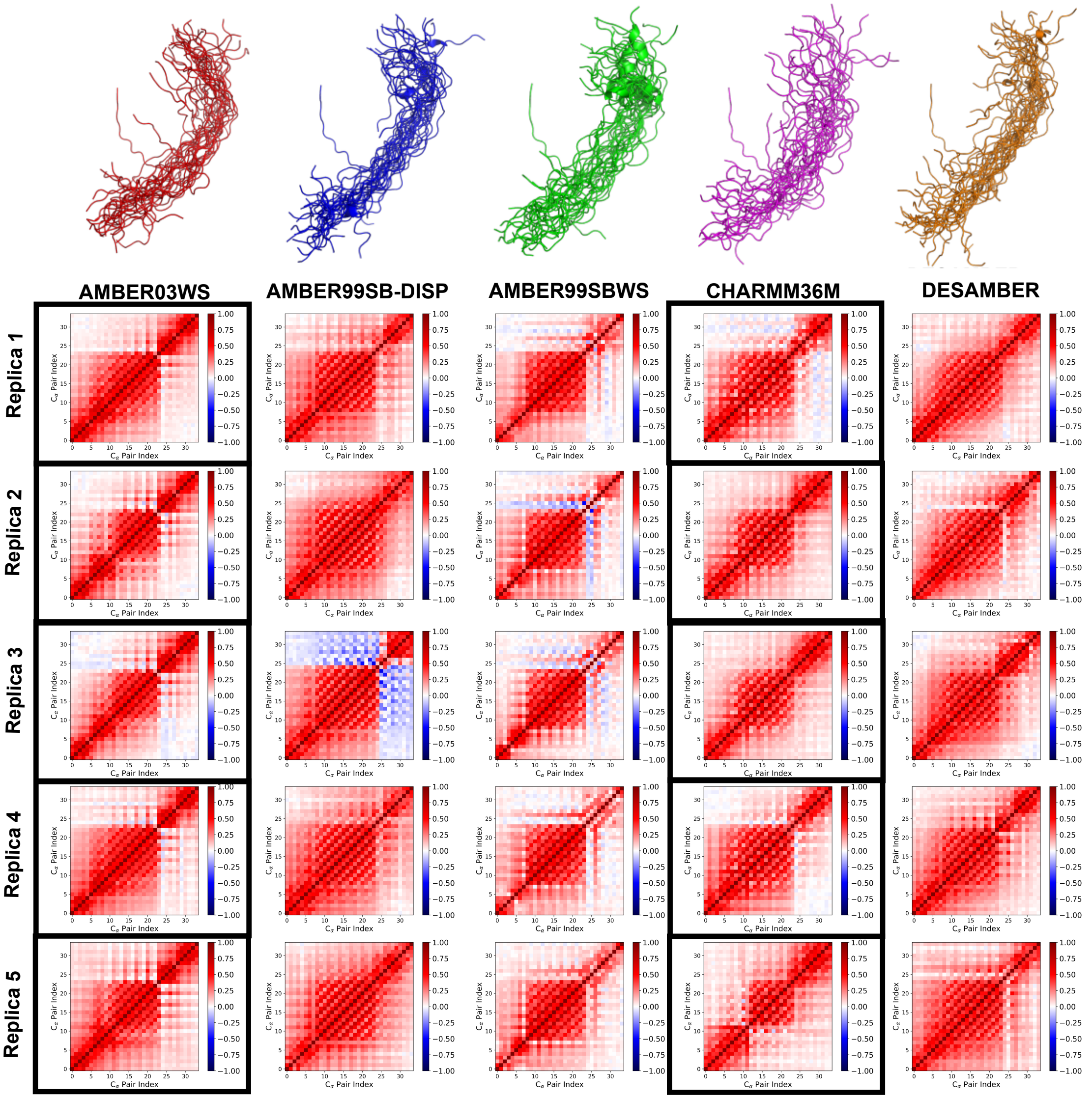

Figure S28: Consecutive Ca vector correlation maps generated from five different initial structures and force fields, along with representative snapshots for the charged peptide. Positive and negative values indicate positive and negative correlation respectively. The snapshots represent 5 overlayed frames from each force field for the same initial structure model of the peptide. The selected simulations from the quality evaluation are highlighted with the square.

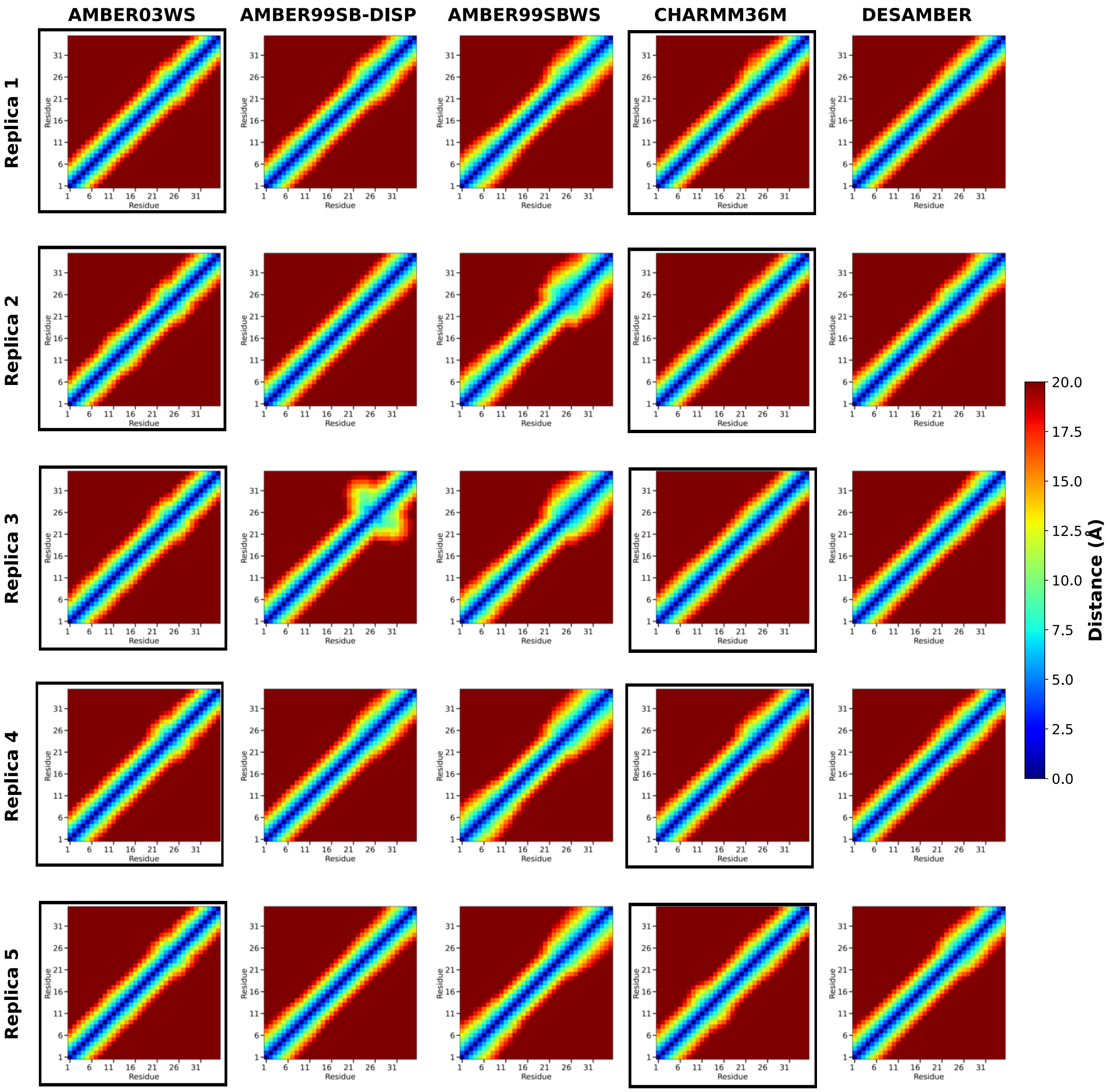

Figure S29: Distance maps for the charged peptide.

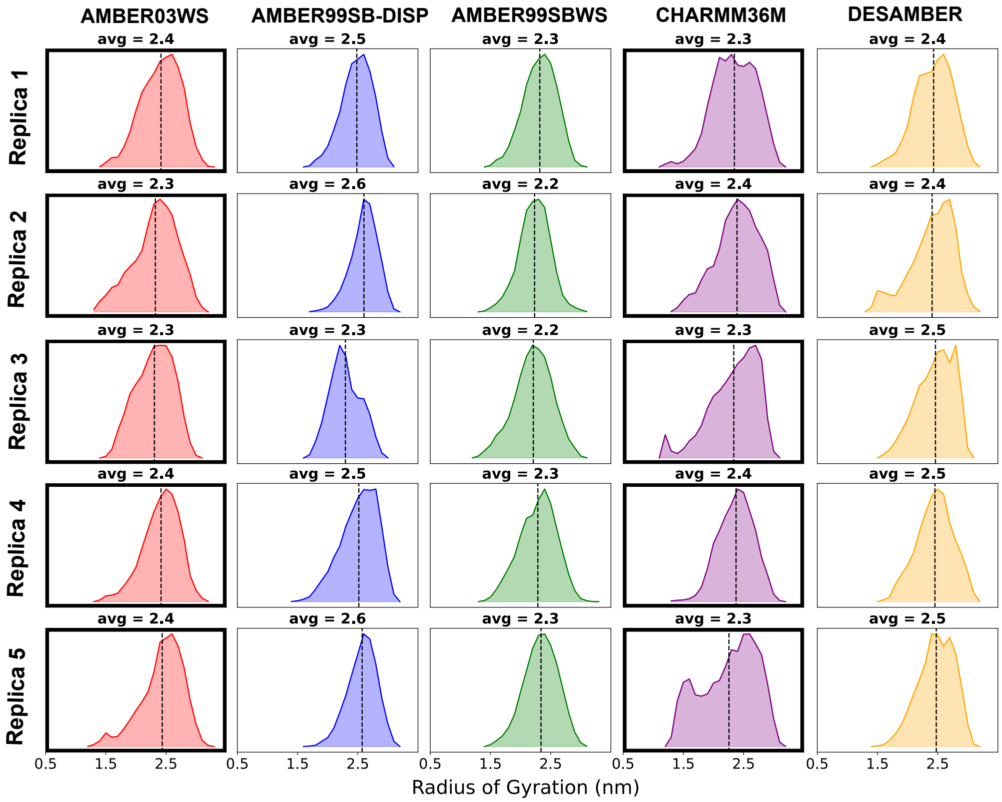

Figure S30: Radius of gyration distribution generated from five different initial structures and force fields for the charged peptide.

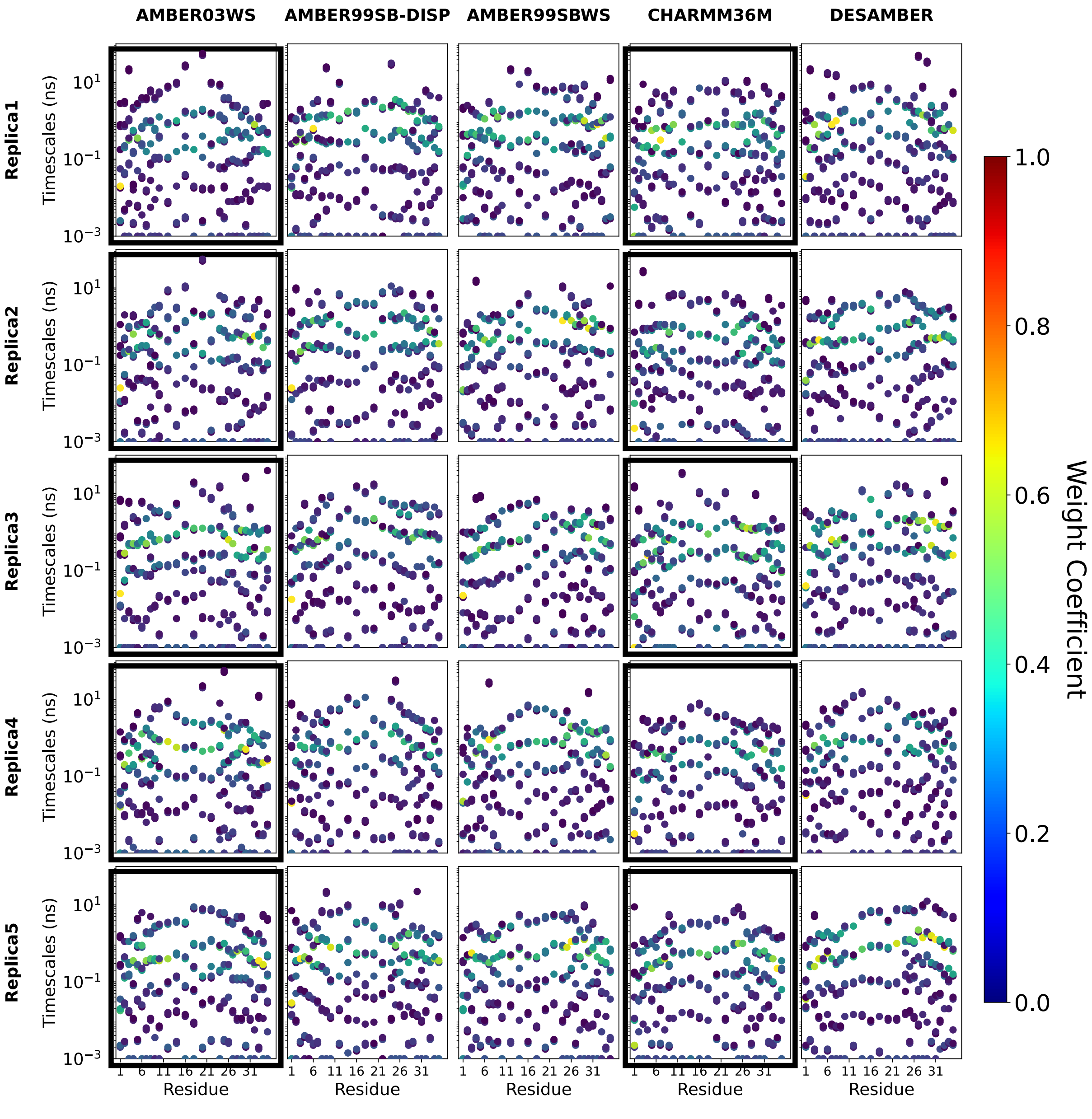

Figure S31: Timescales of backbone N-H bonds in the charged peptide.

Charged sequence with NaCl

Force field	Replica	R1 RMSD	R1 (%)	R2 RMSD	R2 (%)	hetNOE RMSD	hetNOE (%)	Sum (%)
AMBER03WS	replica02	0.073	110	0.14	100	0.11	100	310
AMBER03WS	replica01	0.094	150	0.17	120	0.12	110	380
CHARMM36M	replica02	0.085	130	0.29	200	0.19	180	510
CHARMM36M	replica01	0.078	120	0.33	230	0.18	170	520
CHARMM36M	replica03	0.094	140	0.24	170	0.21	200	520
AMBER99SB-DISP	replica01	0.14	220	0.46	320	0.28	260	810
AMBER99SB-DISP	replica02	0.13	200	0.55	390	0.26	240	830
AMBER03WS	replica04	0.15	230	0.71	500	0.11	100	830
CHARMM36M	replica05	0.093	140	0.75	530	0.21	200	870
AMBER99SBWS	replica02	0.19	290	0.63	440	0.35	320	1100
AMBER99SBWS	replica04	0.18	280	0.68	480	0.32	300	1100
AMBER99SBWS	replica05	0.16	250	0.78	550	0.31	290	1100
AMBER03WS	replica03	0.065	100	1.6	1100	0.11	100	1300
DESAMBER	replica04	0.18	280	1.2	840	0.34	320	1400
AMBER99SBWS	replica01	0.17	260	1.4	970	0.33	310	1500
AMBER99SB-DISP	replica04	0.2	300	1.2	850	0.35	330	1500
AMBER99SB-DISP	replica03	0.16	250	1.5	1100	0.31	290	1600
AMBER03WS	replica05	0.13	200	2.0	1400	0.14	130	1700
CHARMM36M	replica04	0.11	170	2.1	1400	0.2	190	1800
AMBER99SBWS	replica03	0.18	280	1.9	1400	0.29	270	1900
DESAMBER	replica05	0.14	210	3.3	2300	0.25	230	2800
DESAMBER	replica02	0.17	260	4.7	3300	0.33	310	3900
DESAMBER	replica03	0.17	270	4.7	3300	0.34	320	3900
AMBER99SB-DISP	replica05	0.17	270	4.8	3400	0.29	270	3900
DESAMBER	replica01	0.24	370	5.1	3600	0.39	360	4300

Figure S32: charged peptide in NaCl: RMSD and percentage difference to experiment values for relaxation rates R1, R2, and hetNOE. Ranking numbers are determined by their fraction of the lowest RMSD value for each spin relaxation rate. Simulations deviating less than 50 from the lowest RMSD for all spin relaxation rates are selected as best and highlighted in bold.

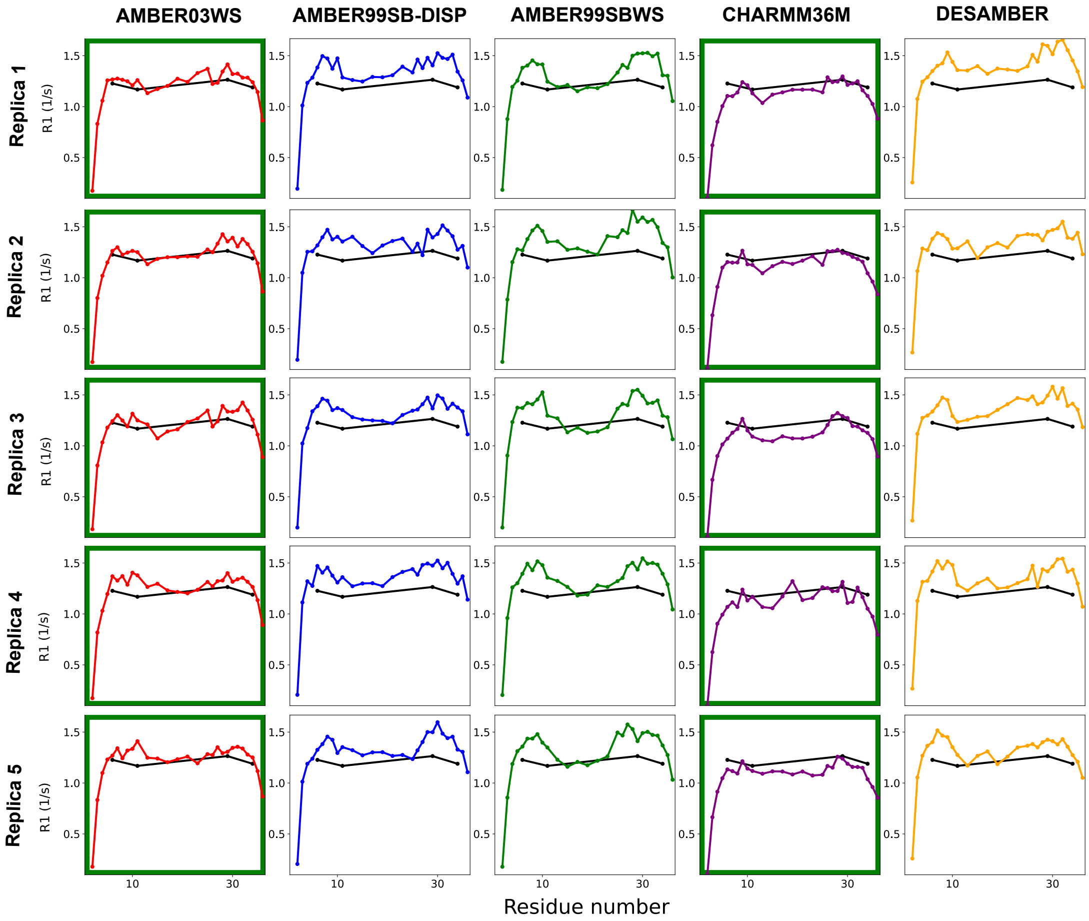

Figure S33: R1 relaxation rate plots for 25 simulations of different models and force fields of charged peptide in the presence of 150mM NaCl.

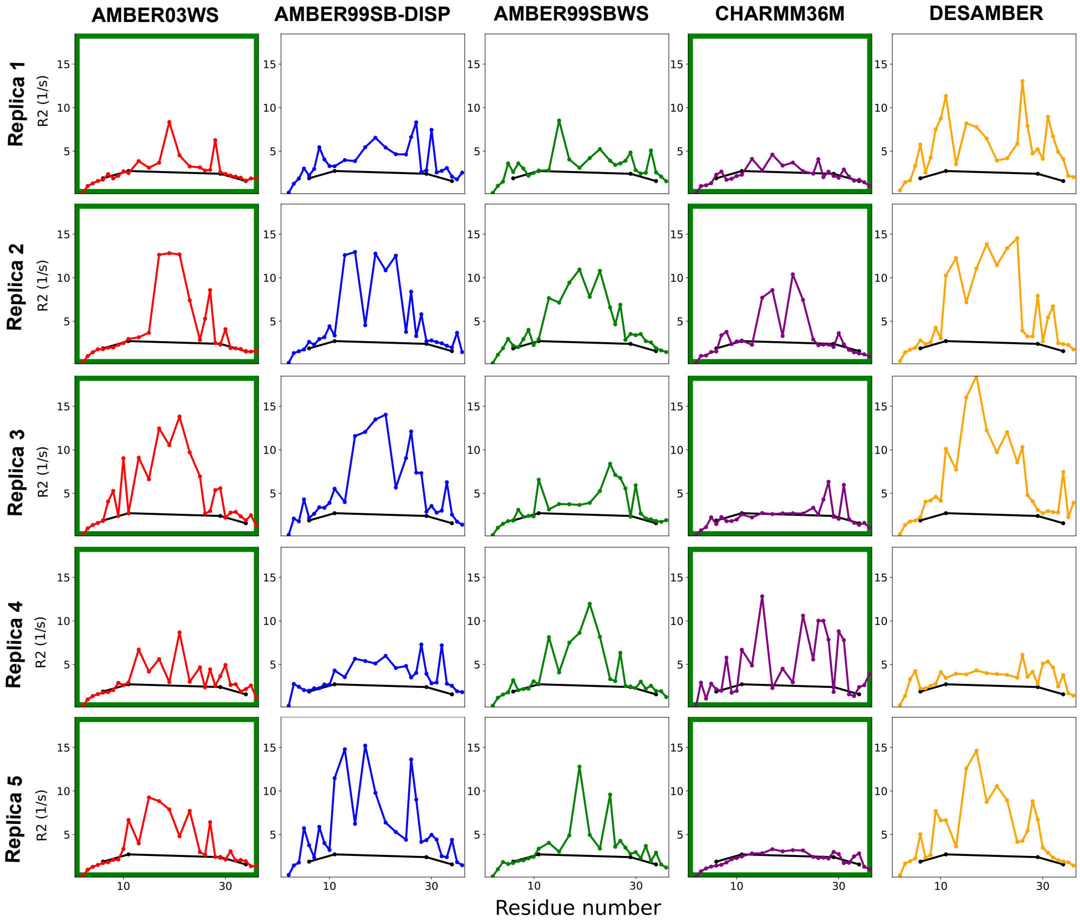

Figure S34: R2 relaxation rate plots for 25 simulations of different models and force fields of charged peptide in the presence of 150mM NaCl.

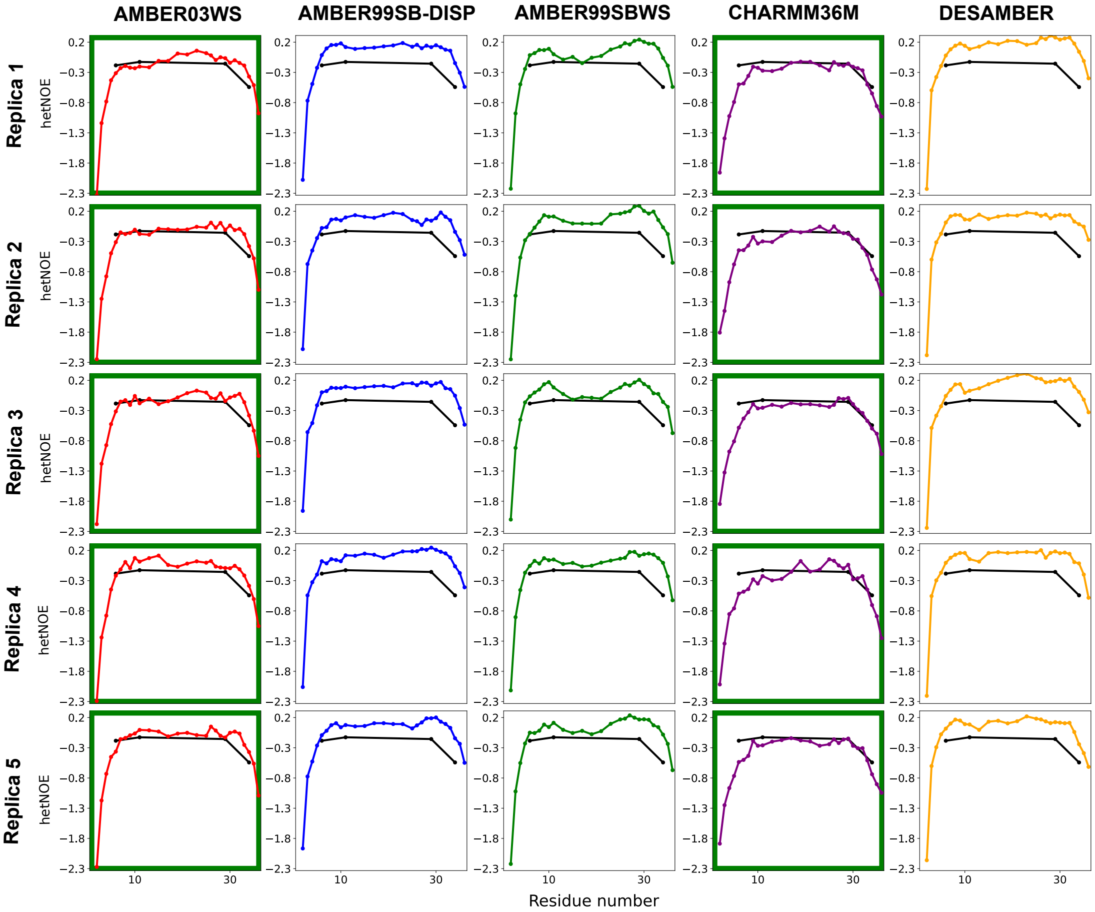

Figure S35: HetNoe relaxation time plots for 25 simulations of different models and force fields of Ton-derived peptide in the presence of 150mM NaCl.

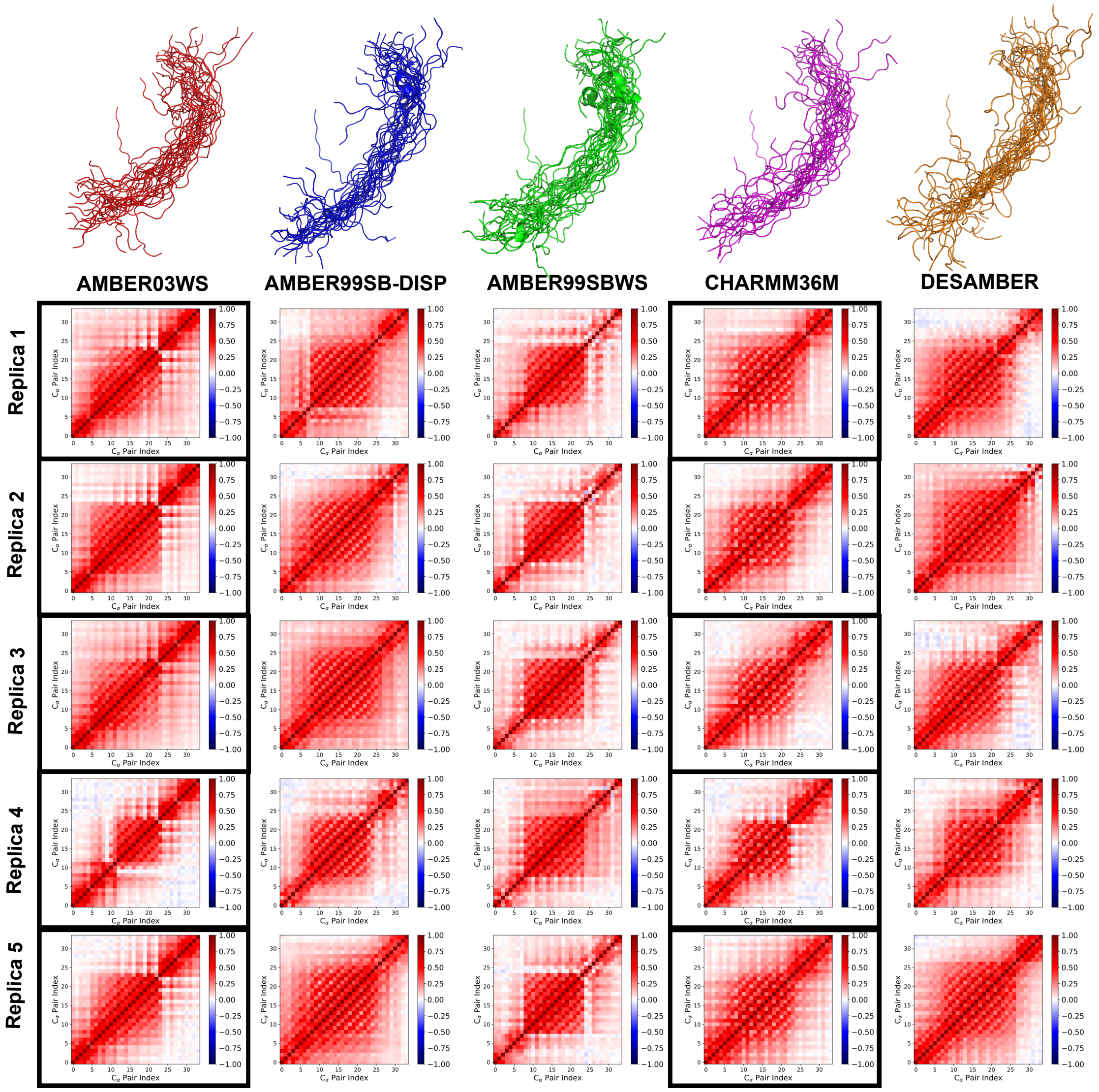

Figure S36: Consecutive Ca vector correlation maps generated from five different initial structures and force fields, along with representative snapshots for the charged peptide in 150mM NaCl. Positive and negative values indicate positive and negative correlation respectively. The snapshots represent 5 overlayed frames from each force field for the same initial structure model of the peptide. The selected simulations from the quality evaluation are highlighted with the square.

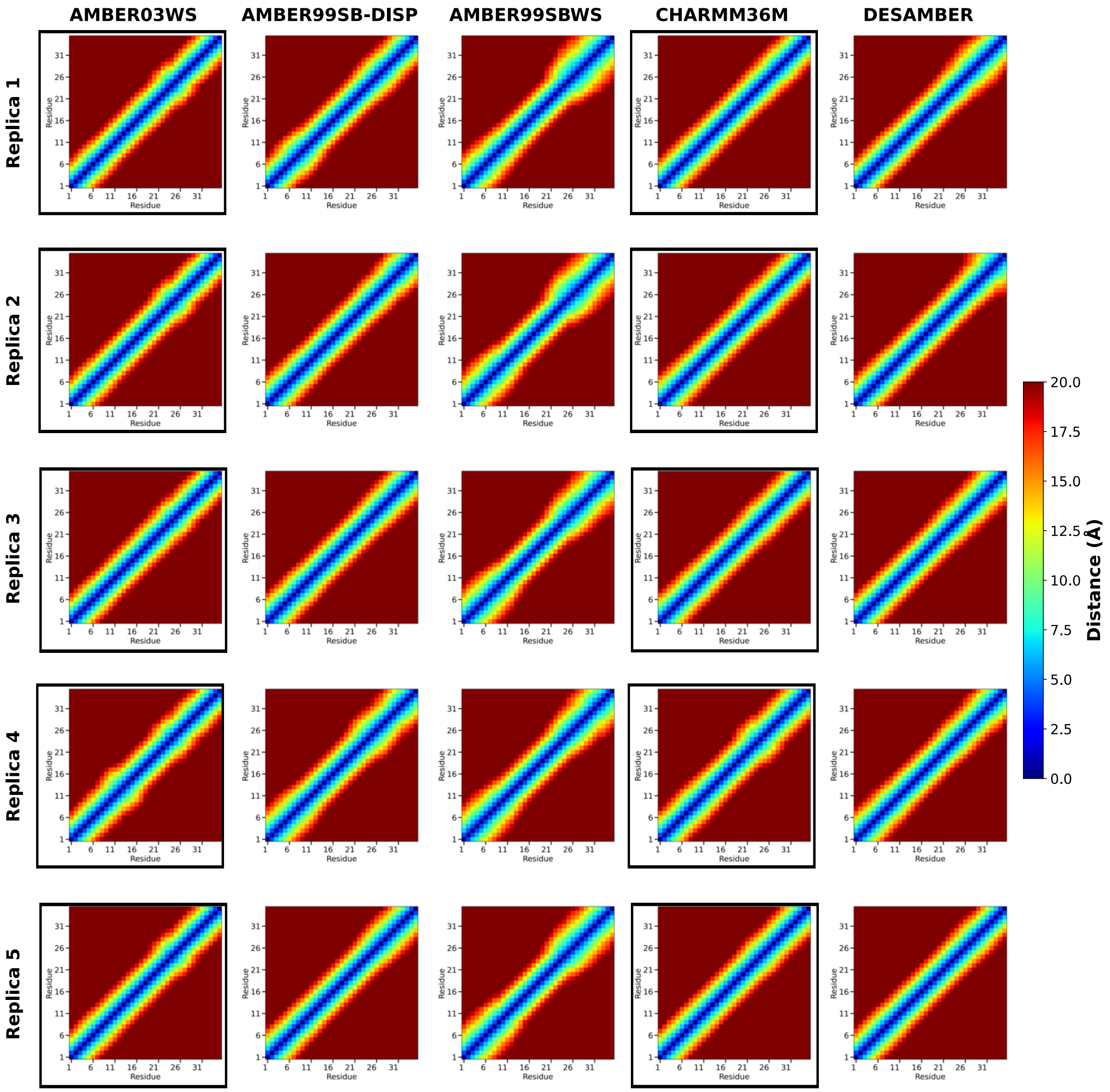

Figure S37: Distance maps for the charged peptide in 150mM NaCl.

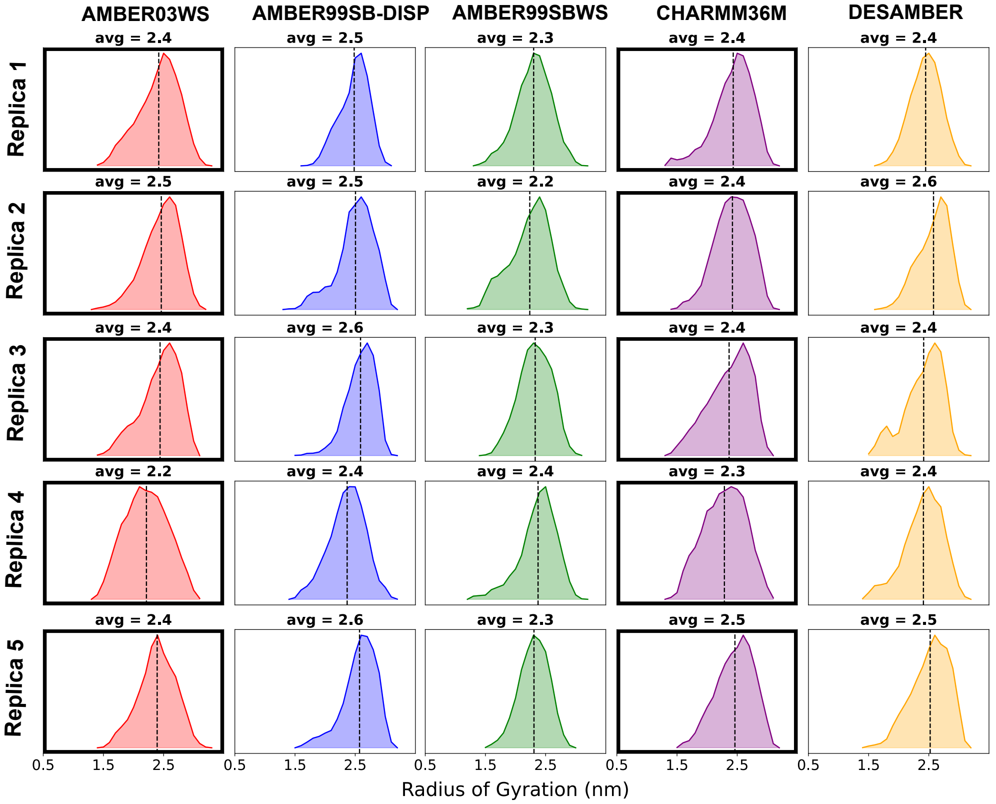

Figure S38: Radius of gyration distribution generated from five different initial structures and force fields for the charged peptide in the presence of 150mM NaCl.

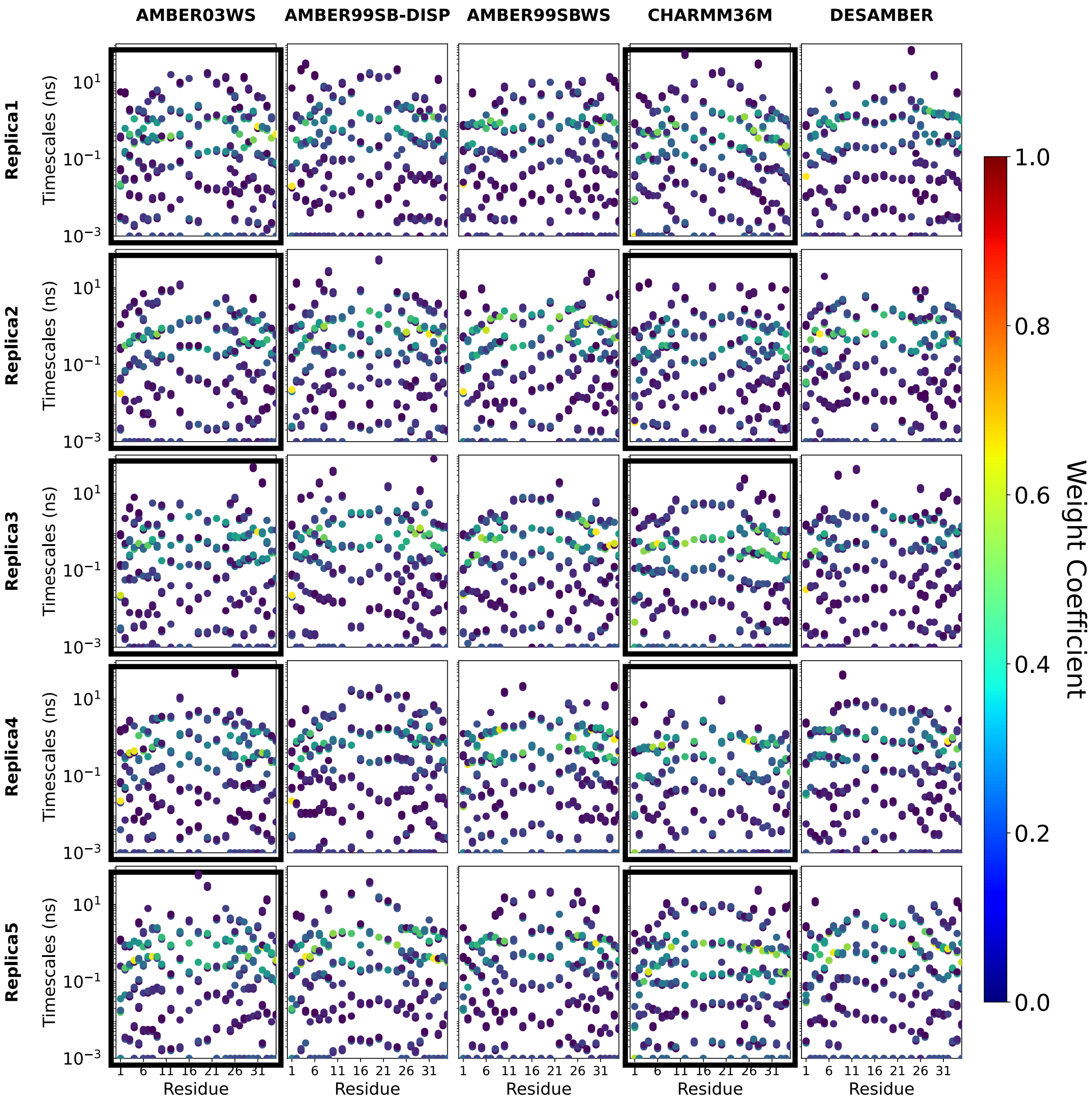

Figure S39: Timescales of of backbone N-H bonds in the charged peptide in 150mM NaCl.

Charged sequence with $CaCl_2$

Force field	Replica	R1 RMSD	R1 (%)	R2 RMSD	R2 (%)	hetNOE RMSD	hetNOE (%)	Sum (%)
CHARMM36M	replica04	0.054	120	0.17	100	0.1	210	430
AMBER99SB-DISP	replica04	0.066	150	0.5	290	0.071	140	580
AMBER99SB-DISP	replica01	0.096	220	0.31	180	0.11	210	610
DESAMBER	replica02	0.06	130	0.68	400	0.049	100	630
DESAMBER	replica03	0.087	190	0.59	350	0.082	170	710
CHARMM36M	replica03	0.13	290	0.2	120	0.21	430	830
CHARMM36M	replica05	0.09	200	0.59	340	0.23	460	1000
CHARMM36M	replica02	0.045	100	0.89	520	0.22	450	1100
DESAMBER	replica05	0.09	200	1.2	700	0.1	210	1100
CHARMM36M	replica01	0.065	150	1.2	690	0.24	480	1300
AMBER99SB-DISP	replica02	0.094	210	1.5	850	0.11	220	1300
AMBER03WS	replica01	0.23	520	0.8	470	0.27	550	1500
AMBER03WS	replica04	0.28	630	1.0	580	0.28	580	1800
AMBER99SBWS	replica04	0.28	640	1.1	660	0.32	640	1900
AMBER99SB-DISP	replica05	0.24	530	1.6	940	0.28	560	2000
AMBER99SB-DISP	replica03	0.12	270	2.6	1500	0.13	270	2100
AMBER99SBWS	replica03	0.27	600	1.3	740	0.37	740	2100
AMBER03WS	replica02	0.25	550	2.2	1300	0.24	490	2300
AMBER99SBWS	replica02	0.17	390	2.5	1400	0.28	570	2400
DESAMBER	replica01	0.11	250	3.5	2100	0.11	230	2500
DESAMBER	replica04	0.065	150	3.8	2200	0.084	170	2600
AMBER03WS	replica05	0.12	260	3.6	2100	0.16	320	2700
AMBER99SBWS	replica05	0.33	750	1.9	1100	0.41	830	2700
AMBER99SBWS	replica01	0.27	600	2.7	1600	0.31	630	2800
AMBER03WS	replica03	0.28	630	2.8	1600	0.33	680	2900

Figure S40: charged peptide in $CaCl_2$: RMSD and percentage difference to experiment values for relaxation rates R1, R2 and hetNOE. Ranking numbers are determined by their fraction of the lowest RMSD value for each spin relaxation rate. Simulations deviating less than 50 from the lowest RMSD for all spin relaxation rates are selected as best and highlighted in bold

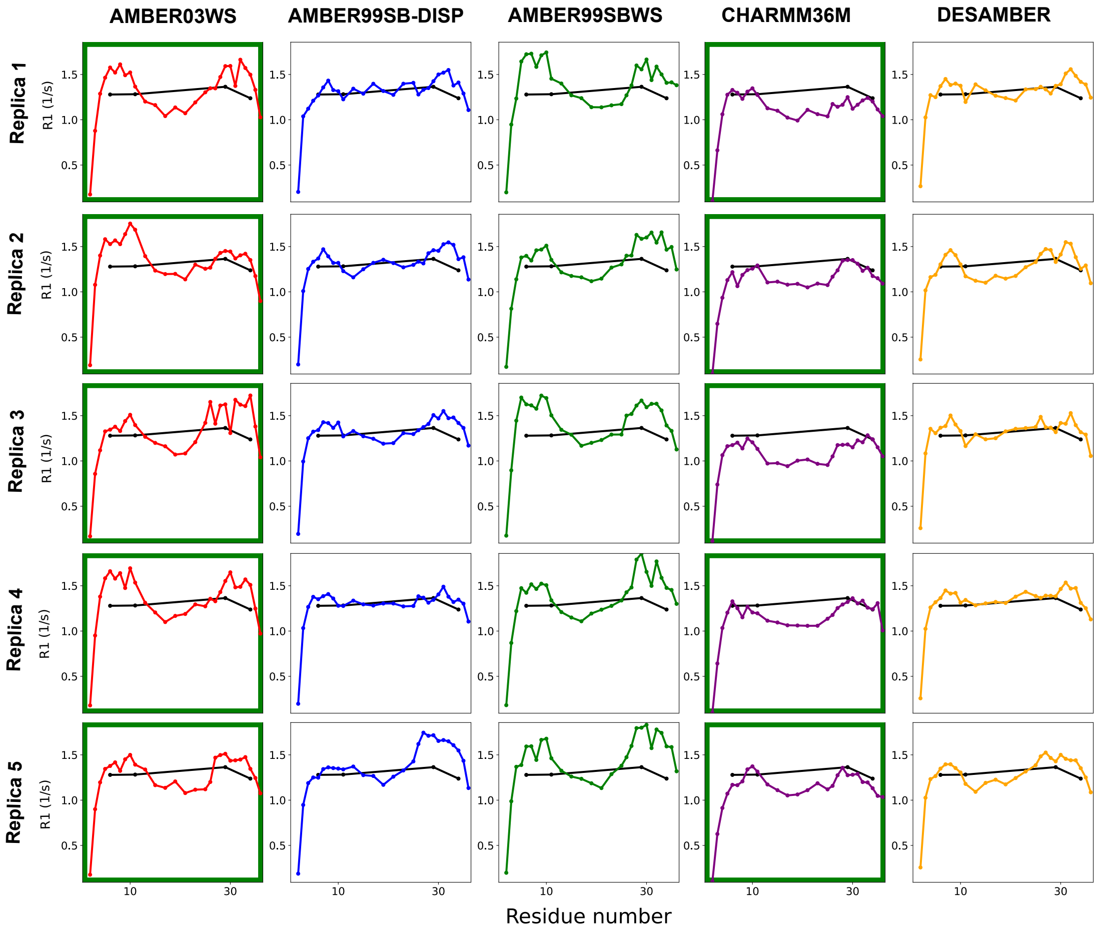

Figure S41: R1 relaxation rate plots for 25 simulations of different models and force fields of charged peptide in the presence of 10mM $CaCl_2$.

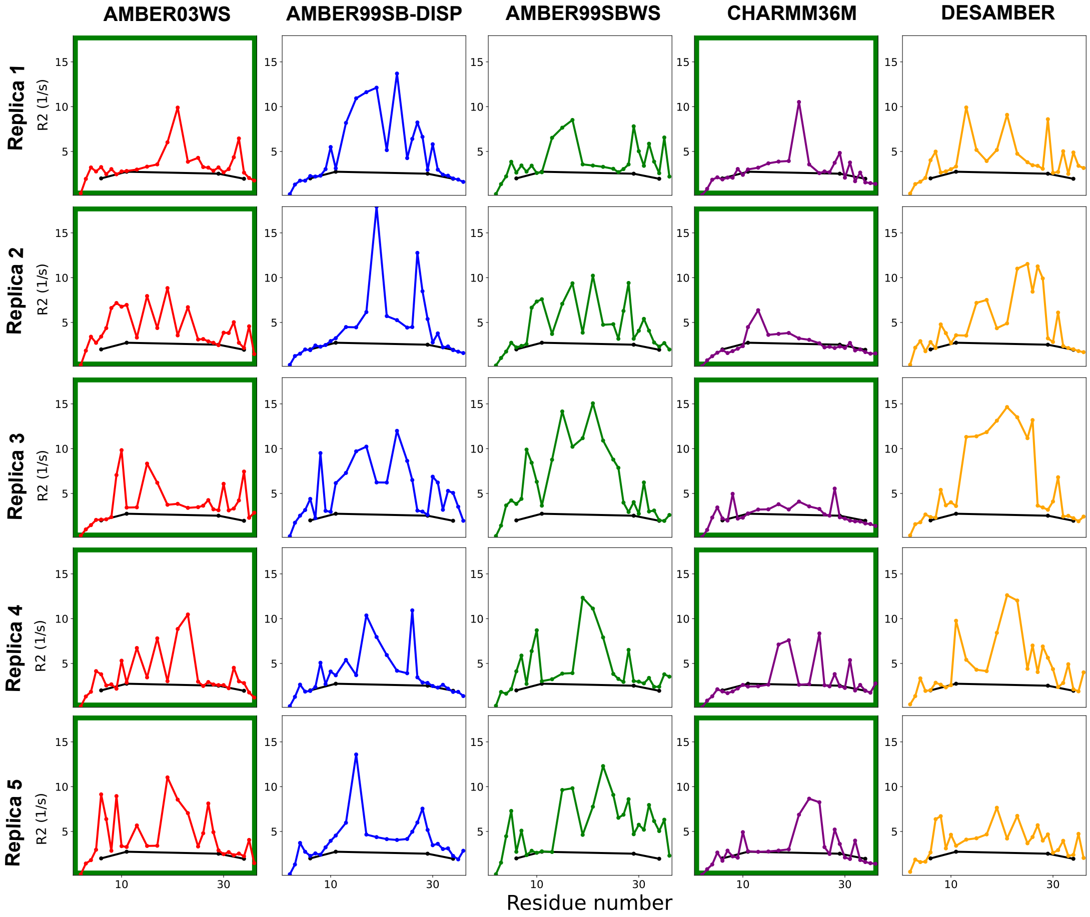

Figure S42: R2 relaxation rate plots for 25 simulations of different models and force fields of charged peptide in the presence of 10mM $CaCl_2$.

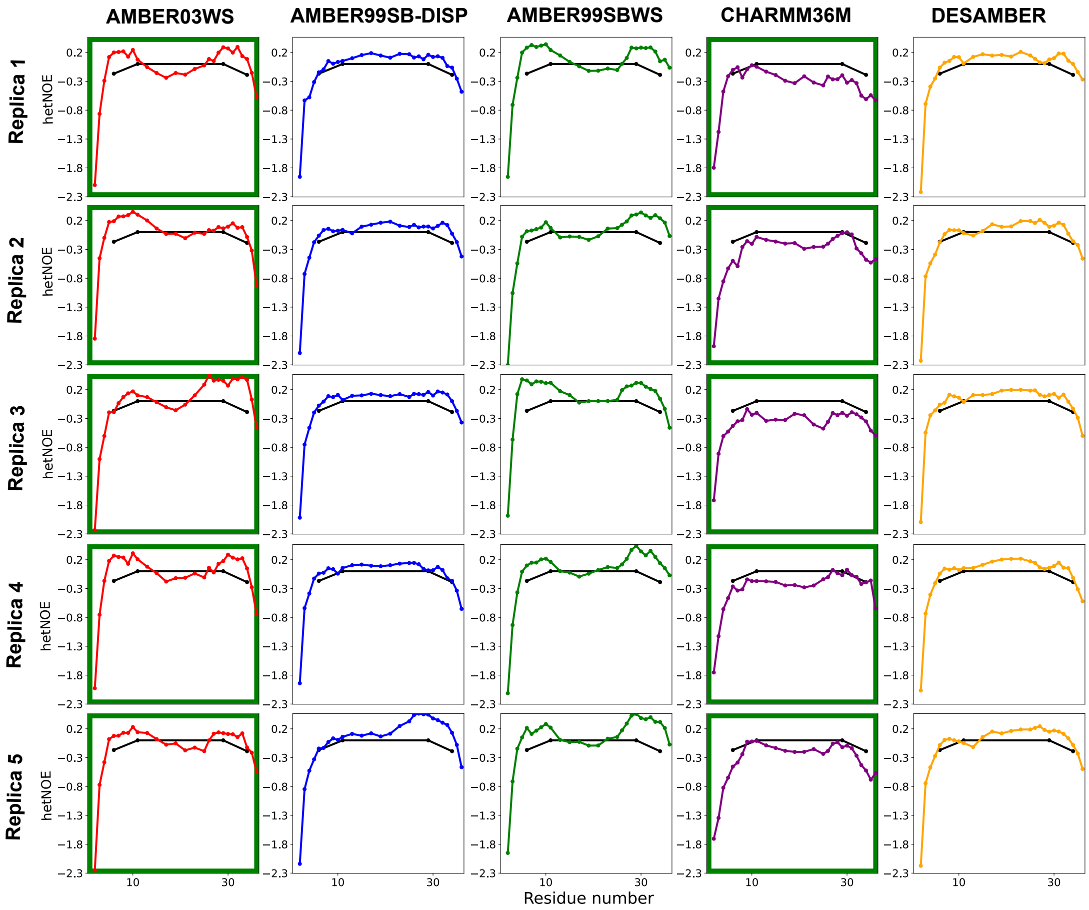

Figure S43: HetNoe relaxation time plots for 25 simulations of different models and force fields of Ton-derived peptide in the presence of 10mM $CaCl_2$.

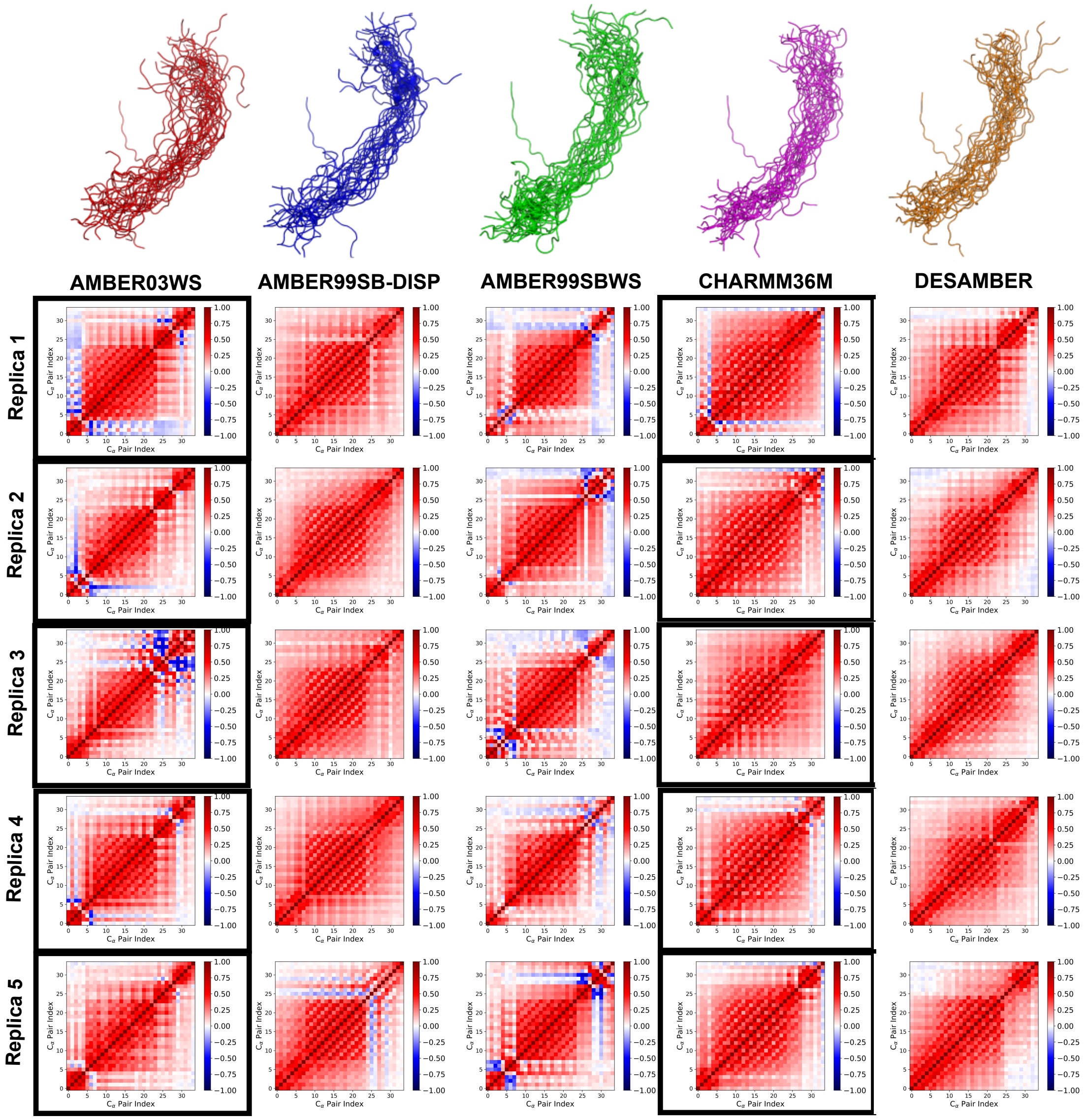

Figure S44: Consecutive Ca vector correlation maps generated from five different initial structures and force fields, along with representative snapshots for the charged peptide in 10mM $CaCl_2$. Positive and negative values indicate positive and negative correlation respectively. The snapshots represent 5 overlayed frames from each force field for the same initial structure model of the peptide. The selected simulations from the quality evaluation are highlighted with the square.

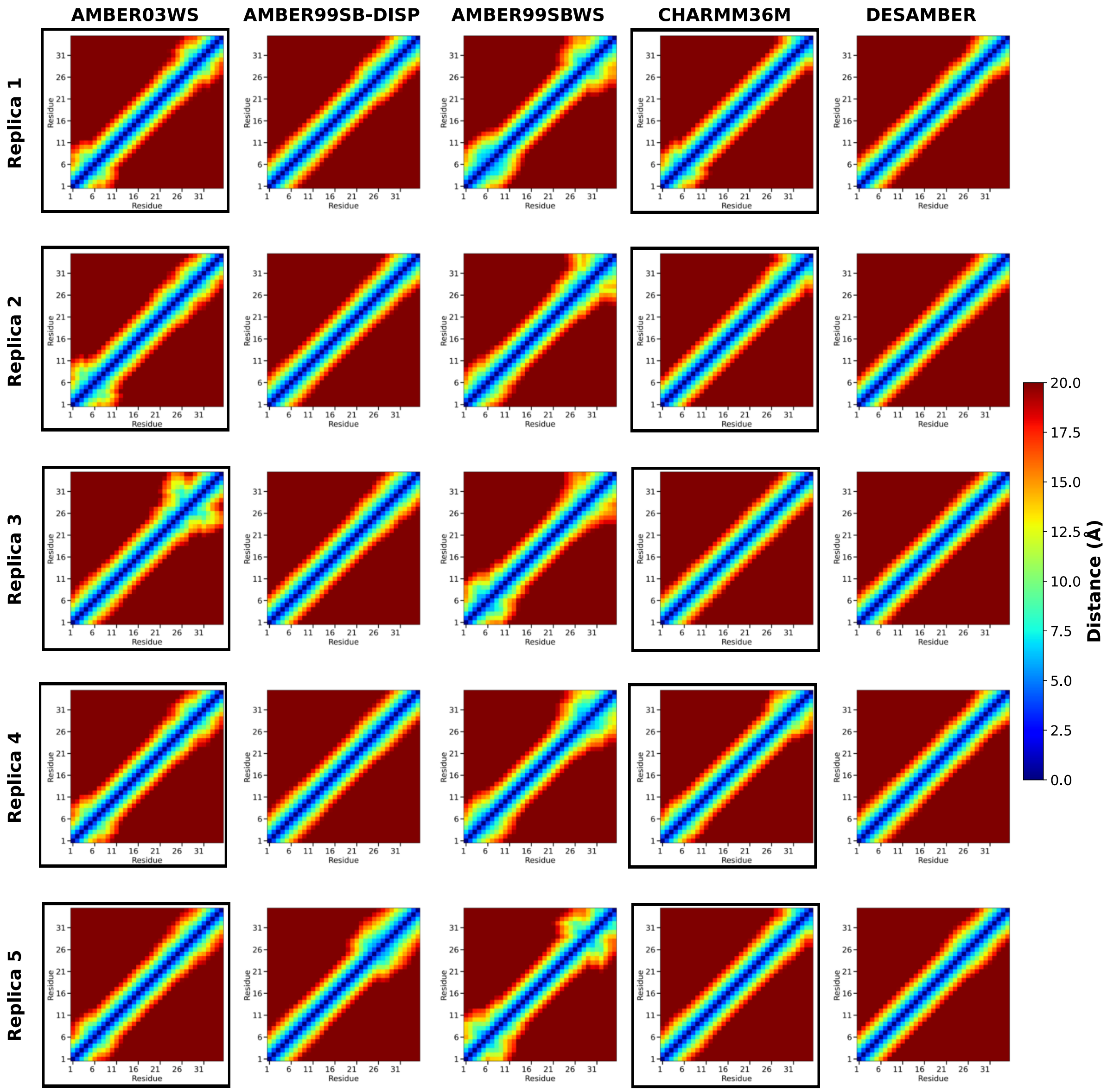

Figure S45: Distance maps for charged peptide in 10mM $CaCl_2$.

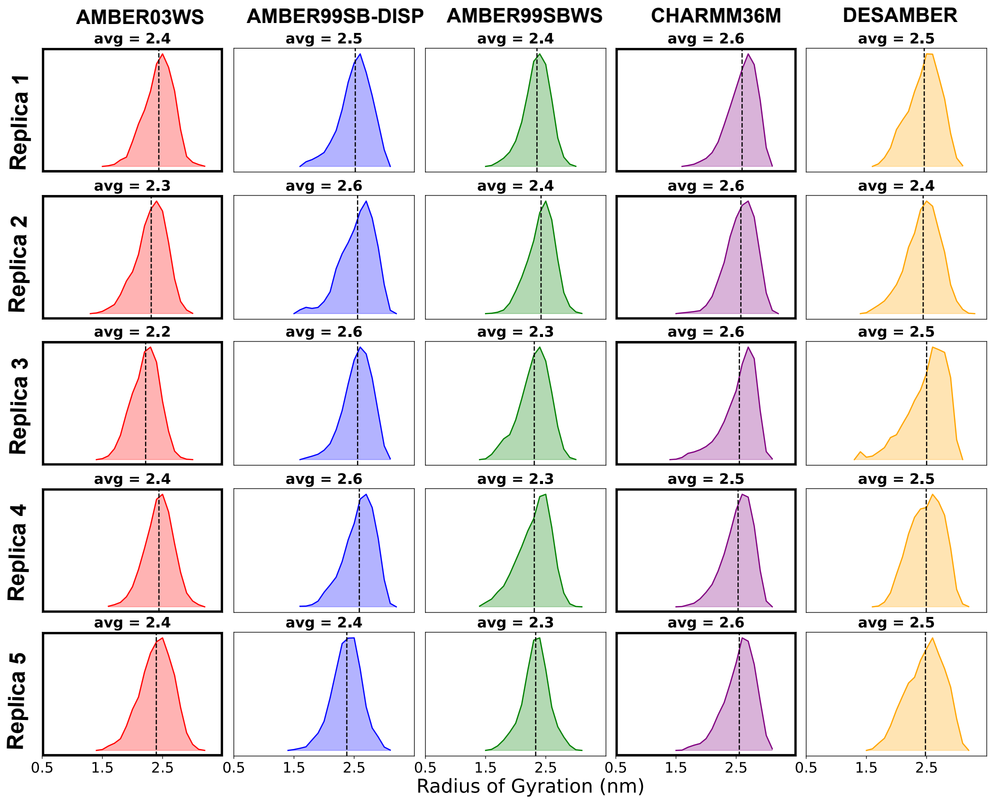

Figure S46: Radius of gyration distribution generated from five different initial structures and force fields for the charged peptide in the presence of 10mM $CaCl_2$.

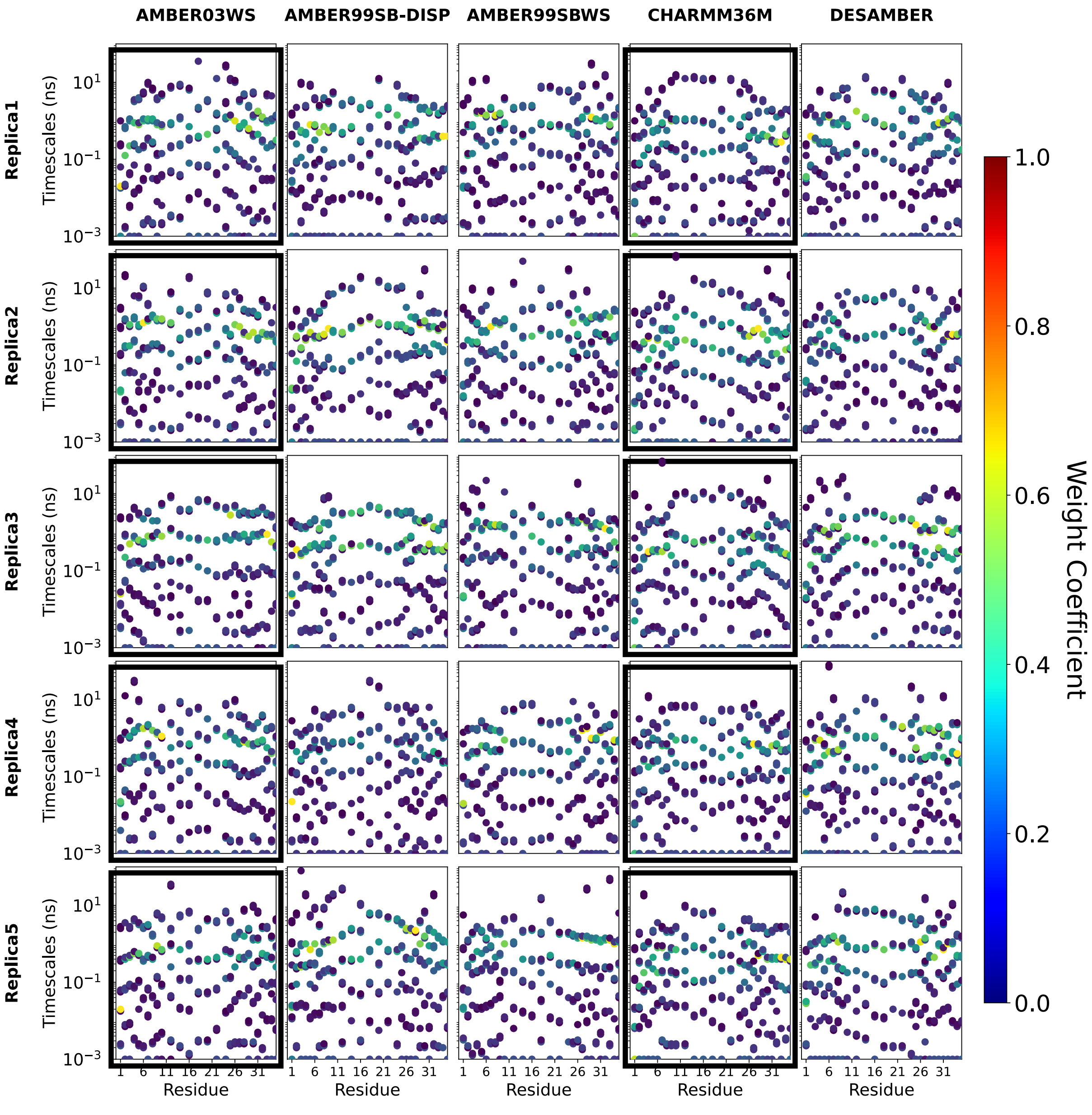

Figure S47: Timescales of of backbone N-H bonds in the charged peptide in 10mM CaCl2.

Effect of sodium and calcium ions on the charged peptide

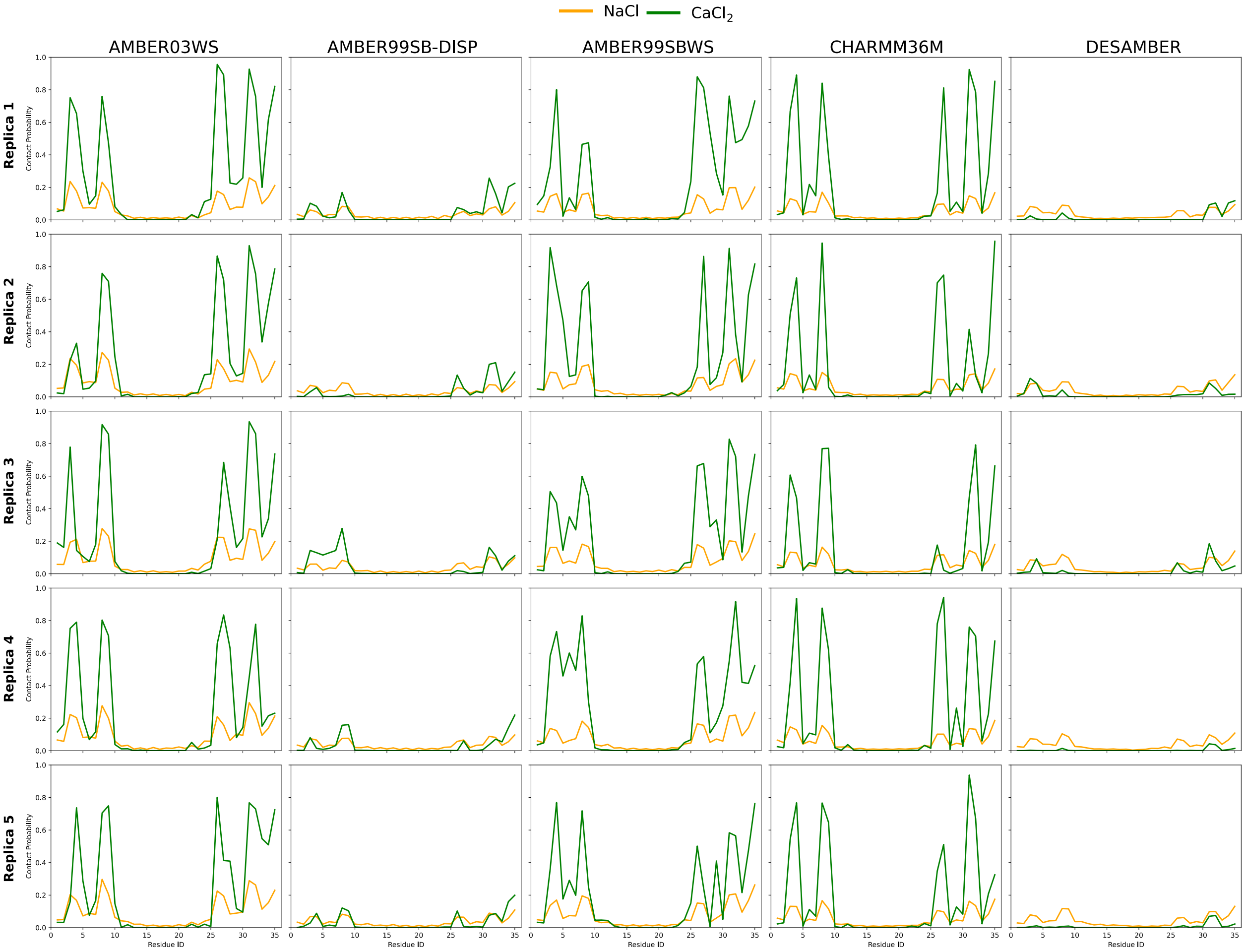

Figure S48: Contact probabilities of calcium and sodium ions with the glutamic acid pairs of the charged peptide over all the simulations.

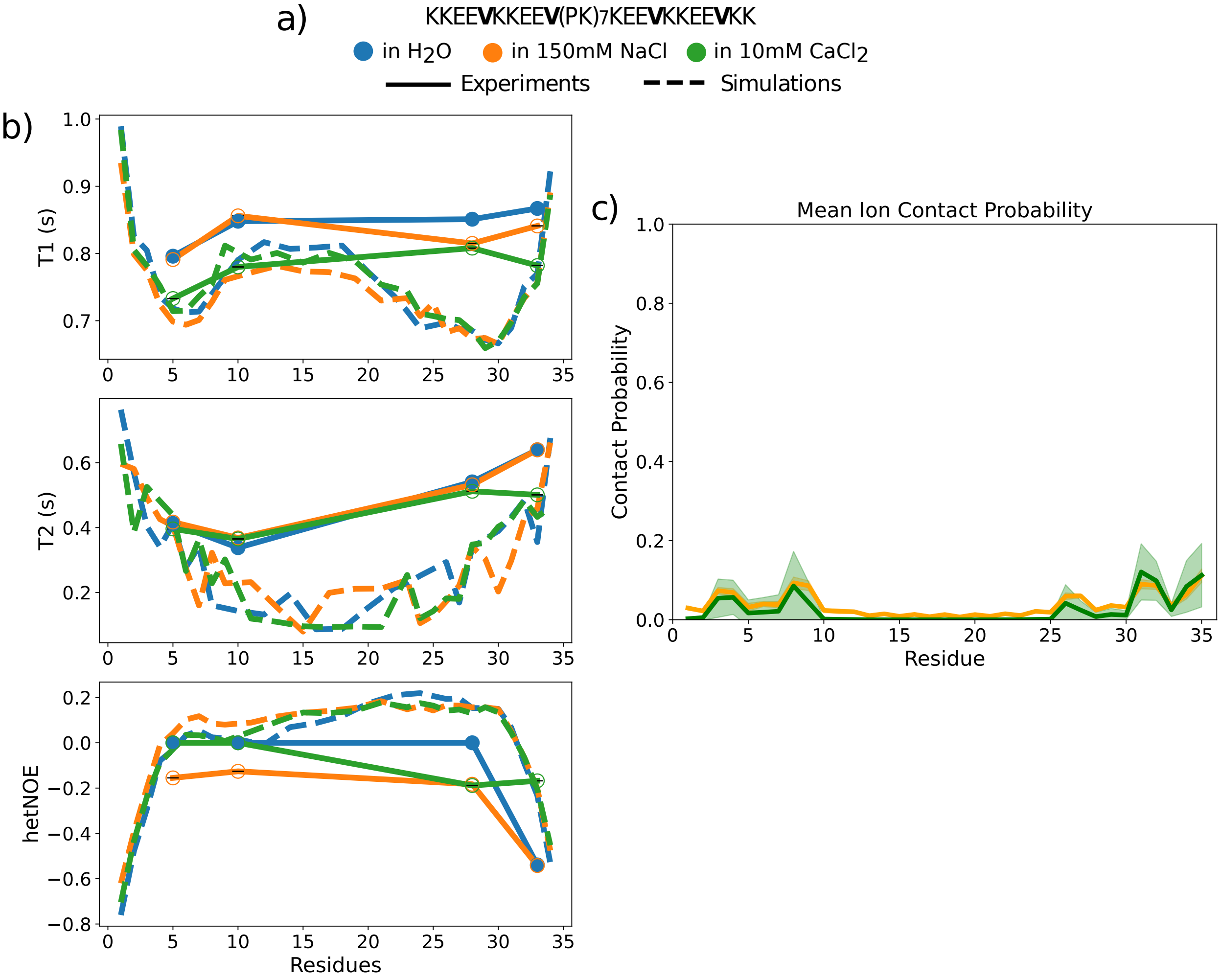

Figure S49: (a) Amino acid sequences of the studied peptide with ^{15}N labeled residues shown in bold. (b) Spin relaxation times from experiments and the AMBER99SB-DISP and DESAMBER simulations. Spin relaxation times from MD simulations of residue 1 was removed due to scaling reasons; Residue one exhibits very fast dynamics leading to long times. (c) Average contact probabilities of calcium and sodium ions with the glutamic acid pairs of the charged peptide over the AMBER99SB-DISP and DESAMBER simulations. The errors were calculated as standard mean errors.

Simulation data availability

Table S1: All simulation data is available in Zenodo repositories.

Protein	Force Field	DOI
$(GGS)_3$	AMBER03WS	10.5281/zenodo.16925399
$(GGS)_3$	AMBER99SB-DISP	10.5281/zenodo.16926107
$(GGS)_3$	AMBER99SBWS	10.5281/zenodo.16926198
$(GGS)_3$	CHARMM36M	10.5281/zenodo.16926256
$(GGS)_3$	DESAMBER	10.5281/zenodo.16926284
$(GPS)_3$	AMBER03WS	10.5281/zenodo.16926303
$(GPS)_3$	AMBER99SB-DISP	10.5281/zenodo.16926459
$(GPS)_3$	AMBER99SBWS	10.5281/zenodo.16926477
$(GPS)_3$	CHARMM36M	10.5281/zenodo.16926503
$(GPS)_3$	DESAMBER	10.5281/zenodo.16926510
$K(AP)_5K$	AMBER03WS	10.5281/zenodo.16926564
$K(AP)_5K$	AMBER99SB-DISP	10.5281/zenodo.16928503
$K(AP)_5K$	AMBER99SBWS	10.5281/zenodo.16928511
$K(AP)_5K$	CHARMM36M	10.5281/zenodo.16928545
$K(AP)_5K$	DESAMBER	10.5281/zenodo.16928586
Charged sequence	AMBER03WS	10.5281/zenodo.16928640
Charged sequence	AMBER99SB-DISP	10.5281/zenodo.16928790
Charged sequence	AMBER99SBWS	10.5281/zenodo.16929062
Charged sequence	CHARMM36M	10.5281/zenodo.16929122
Charged sequence	DESAMBER	10.5281/zenodo.16929115
Charged sequence with NaCl	AMBER03WS	10.5281/zenodo.16929574
Charged sequence with NaCl	AMBER99SB-DISP	10.5281/zenodo.16931819
Charged sequence with NaCl	AMBER99SBWS	10.5281/zenodo.16932024
Charged sequence with NaCl	CHARMM36M	10.5281/zenodo.16932035
Charged sequence with NaCl	DESAMBER	10.5281/zenodo.16932041
Charged sequence with $CaCl_2$	AMBER03WS	10.5281/zenodo.16932078
Charged sequence with $CaCl_2$	AMBER99SB-DISP	10.5281/zenodo.16932100
Charged sequence with $CaCl_2$	AMBER99SBWS	10.5281/zenodo.16932128
Charged sequence with $CaCl_2$	CHARMM36M	10.5281/zenodo.16932180
Charged sequence with $CaCl_2$	DESAMBER	10.5281/zenodo.16932203
