# Supplementary material for: Backbone rigidity of disordered protein linkers from NMR experiments and MD simulations
Source: Biophys J. 2026 Mar 10;125(7):1753–64. doi: 10.1016/j.bpj.2026.03.009 (PMC13351908; doi:10.1016/j.bpj.2026.03.009)
Supplement: Document S2. Article plus supporting material [file mmc2.pdf]

Biophysical Journal
Article

Biophysical Society

Backbone rigidity of disordered protein linkers from NMR experiments and MD simulations

Efstathia Mantzari,[1,2,3] Cajsa K. Malm,[1] Ricky Nencini,[2,4] Amanda E. Sandelin,[2,5] and O. H. Samuli Ollila[1,2,*]
[1]VTT Technical Research Centre of Finland, 02044 Espoo, Finland; [2]Institute of Biotechnology, University of Helsinki, 00014 Helsinki, Finland; [3]Department of Chemistry and Materials Science, Aalto University, 00076 Espoo, Finland; [4]Division of Pharmaceutical Biosciences, Faculty of Pharmacy, University of Helsinki, 00014 Helsinki, Finland; and [5]Division of Pharmacology and Pharmacotherapy, Faculty of Pharmacy, University of Helsinki, 00014 Helsinki, Finland

ABSTRACT Disordered protein linkers are essential for multidomain protein function and engineering, but quantitative methods for their biophysical characterization remain limited. We combined NMR experiments with molecular dynamics simulations to demonstrate that protein backbone ^{15}N spin relaxation times correlate with backbone rigidities in short, disordered linkers. Using a tailored version of the Quality Evaluation Based Simulation Selection framework, we characterized four model peptides: $(GGS)_3$, $(GPS)_3$, $K(AP)_5K$, and KKEEVKKEEV-$(PK)_7$KEEVKKEEVKK, representing common natural and engineered linker repeats. Glycine-rich sequences showed slight looping tendencies, whereas proline-containing sequences adopted extended conformations with increased approximate persistence lengths and slower dynamics. Notably, sodium and calcium binding to charged peptides minimally affected rigidity, indicating electrostatics don't dominate linker stiffness. This integrated approach provides quantitative insights into disordered linker properties and MD simulation accuracy, offering biophysical understanding for protein design and machine learning model development.

SIGNIFICANCE Disordered protein linkers are critical for multidomain protein function and engineering, yet quantitative methods to characterize their biophysical properties remain limited. This study introduces an integrated approach combining NMR spin relaxation measurements with molecular dynamics simulations to quantitatively characterize backbone rigidity in short, disordered peptides. By demonstrating that protein backbone ^{15}N spin relaxation times correlate with backbone rigidity, we provide a framework for quantitative characterization of these important protein regions. Our results quantify sequence effects—particularly proline content—on linker stiffness while showing electrostatics play a minor role. This methodology addresses a critical gap and offers insights for rational protein design in biotechnology while providing benchmark data for validating computational models and developing machine learning approaches for predicting linker properties.

INTRODUCTION

Most proteins are composed of multiple folded domains that are connected by linker domains (1). Besides their biological relevance, protein linkers can be used in a wide range of protein engineering applications, from self-assembling proteins (2) to functional proteins for creating active materials used in drug discovery (3), electronics (4,5), biosensing (6), antibody and nanobody design (7), or to stabilize protein structures for structural studies (8). Depending on their amino acid sequence, these linkers may fold to rigid alpha helical structures or remain as disordered chains with varying stiffness (9,10). Several sequences are known to form rigid α-helical linkers, whereas different combinations of aliphatic and hydrophilic residues, such as lysine, glutamate, glycine, serine, and alanine, compose flexible and disordered linkers, which can be rigidified by the presence of prolines without forming stable α-helices (9,11). Rigid alpha helical linkers can be characterized using multiple experimental techniques (12,13) and also designed computationally with high accuracy (9,14). However, properties of disordered linkers can be derived from their sequence only on an approximate level, and experimental methods for their characterization are sparse (15).

Submitted November 21, 2025, and accepted for publication March 3, 2026.
*Correspondence: samuli.ollila@vtt.fi
Editor: Scott Showalter
https://doi.org/10.1016/j.bpj.2026.03.009
© 2026 The Author(s). Published by Elsevier Inc. on behalf of Biophysical Society.
This is an open access article under the CC BY license (http://creativecommons.org/licenses/by/4.0/).

Backbone rigidities of disordered proteins have been previously measured quantitatively using fluorescence resonance energy transfer (16–19) and force spectroscopy (20). Also, molecular dynamics (MD) simulations have been used (21,22). However, comparison studies reveal that their results for disordered proteins depend on the used force field parameters and should be therefore carefully evaluated against experimental data (23–28). The recently introduced Quality Evaluation Based Simulation Selection (QEBSS) approach (26,29) selects conformational ensembles from a pool of diverse MD simulations based on agreement with experimental protein backbone ^{15}N-H longitudinal (T_1) and transverse (T_2) spin relaxation times and heteronuclear NOE (hetNOE) from NMR experiments. QEBSS was used previously to characterize conformational ensembles and dynamics of multidomain (29) and disordered (26) proteins.

Here, we use a tailored version of this approach to demonstrate that spin relaxation times correlate with backbone rigidities of common disordered protein linkers. We characterize backbone rigidities of four model peptides, $(GGS)_3$, $(GPS)_3$, $K(AP)_5K$, and KKEEVKKEEV$(PK)_7$KEEVKKEEVKK, composed of common repeats in natural and engineered disordered linkers (9,30–33). The 33-amino-acid peptide with highly charged lysine and glutamic acid-rich ends and a proline-lysine repeat region at the middle is coined as the charged peptide from now on. Glycine-serine repeats are common in flexible linkers and XP (X: alanine, lysine or glutamic acid, P: proline) repeats typically impose conformational restrains (9,32). Potential applications of alanine-proline repeats as PEG polymer replacements are proposed due to their inherited disordered nature and high solubility (33). The sequence of the charged peptide is inspired by the disordered region of TonB protein, which reaches across the periplasmic space in Gram-negative bacteria (25,34). Similar charge patterns are also found, for example, in molecular chaperone protein Hsp90, where a negatively charged flexible linker plays a role in the conformational response of the protein upon substrate binding and serves as a posttranslation modification hotspot, suggesting a possible regulatory role (35).

Our results demonstrate that disordered protein backbone rigidity can be quantitatively characterized by combining spin relaxation data with MD simulations. Although proline addition is known to rigidify linkers (9,32), experimental methods to quantify this rigidification have been lacking. Our approach does not only provide quantitative persistence lengths describing the backbone rigidity, but it provides also more detailed information in terms of pairwise backbone orientational and distance maps. Furthermore, we demonstrate that the approach can be used to investigate dependence of backbone rigidity on external conditions such as salt concentration.

Our results advance fundamental knowledge on behavior of disordered protein linkers. This can support the design of linkers for protein engineering applications in protein-based biomaterials, bionanotechnology, synthetic biology, and bioelectronics. Furthermore, our results can provide insights on biological functions of disordered protein sequences.

MATERIALS AND METHODS

Quality Evaluation Based Simulation Selection for short peptides

We utilize here the QEBSS protocol that was previously introduced for two-domain proteins (29) and then automatized for disordered proteins (26) (available at https://github.com/vttresearch/QEBSS/). The main approach is similar to previous studies, but here, we have tailored some details to better function for short peptides with experimental spin relaxation times available only for few residues. As previously, QEBSS is implemented in five steps.

Initial configurations

Five independent initial configurations of peptides with the sequences of $(GGS)_3$, $(GPS)_3$, $K(AP)_5K$, and KKEEVKKEEV$(PK)_7$KEEVKKEEVKK were generated using the IDPConfomerGenerator (36). Five different torsion angle databases, downloaded from the PISCES database (http://dunbrack.fccc.edu/lab/pisces (37)) were used as an input for IDPConformerGenerator. Lists of conformers from each database were then generated using IDPConformerGenerator with options *–dstrand* and *–dloop-off*. One configuration from each generated list of conformations was then manually selected for the starting configuration of a given replica such that it was not similar to any previous selected configuration for the given peptide. Amino acids were set to their default protonation states at pH 7.

Running simulations

From each initial structure generated in step 1, five simulations were launched using different force fields listed in Table 1. CHARMM36M was used with TIP4P water model because its default TIP3P water model has unrealistic viscosity leading to incorrect rotational dynamics and therefore inaccurate spin relaxation times (48). Each peptide configuration was placed in a dodecahedron simulation box, using the *gmx editconf* gromacs command (49). The systems were solvated with 10,896, 10,688, 23,968, and 142,752 water molecules for $(GGS)_3$, $(GPS)_3$, $K(AP)_5K$, and KKEEVKKEEV$(PK)_7$KEEVKKEEVKK, respectively, using *gmx solvate*. Neutralizing ions were added using *gmx genion*. Default ions parameters provided with force fields were used. For the simulations examining the effect of ion concentration for the KKEEVKKEEV$(PK)_7$KEEVKKEEVKK peptide, extra sodium and chloride ions were added to reach the ionic strength of 150 mM and extra calcium and chloride ions to reach 10 mM ionic strength using *gmx genion*.

After energy minimization, NVT, and NPT equilibration, the systems were simulated for 1 μs with 2-fs timestep using GROMACS 2024.1 (49) with periodic boundary conditions. Temperature was coupled using velocity rescaling with a stochastic term at 303 K (v-rescale) (50) and pressure coupled to 1 bar with isotropic Parrinello-Rahman barostat (51). Particle mesh Ewald (52) was used for electrostatics in all simulations. Lennard-Jones cutoff values, constraint algorithms, and water models used with each force field are listed in Table 1.

All simulation trajectories and relevant files for their reproduction are available in Zenodo (https://zenodo.org/) repositories that are listed in Table S1.

Calculation of spin relaxation times from MD simulation trajectories

Spin relaxation times, T_1 and T_2, and hetNOE values were calculated from MD simulations using Redfield equations (53,54) with the previous

TABLE 1 Force field descriptions with the respective water model used in the simulations and specific parameters

Force field	LJ cutoff (nm)	Constraint for hydrogen bond	Water model	Description
AMBER03WS (38)	1.4	LINCS	TIP4P/2005s (39)	rescaled protein-water interactions from AMBER03 force field (40)
AMBER99SBWS (38)	1.4	LINCS	TIP4P/2005s (39)	based on Amber ff99SB*-ILDN-Q force field (41,42), with scaled protein-water interactions.
AMBER99SB-DISP (23)	1.2	SHAKE	a99SB-disp (23)	optimization of backbone torsion, Lennard-Jones term based on the amber99SB-ILDN force field (41).
CHARMM36M (43)	1.0	LINCS	TIP4P (44)	optimized description of specific salt bridge interactions, based on CHARMM36 force field (45).
DESAMBER (46)	1.2	SHAKE	TIP4P-D (47)	developed for ordered and disordered proteins as well as protein-protein complexes, based on the AMBER99SB-DISP force field (23).

implementation (25,29,48,55) that is described in detail in Ref. (26). Spin relaxation rates are inverse of spin relaxation times, $R_1 = 1/T_1$ and $R_2 = 1/T_2$,

$$\frac{1}{T_1} = \frac{d_{\mathrm{NH}}^2 N_{\mathrm{H}}}{20}\left[J(\omega_{\mathrm{H}} - \omega_{\mathrm{N}}) + 3J(\omega_{\mathrm{N}}) + 6J(\omega_{\mathrm{H}} + \omega_{\mathrm{N}})\right] + \frac{(\rho\omega_{\mathrm{N}})^2}{15}J(\omega_{\mathrm{N}}) \tag{1}$$

$$\frac{1}{T_1} = \frac{1}{2}\frac{d_{\mathrm{NH}}^2 N_{\mathrm{H}}}{20}\left[4J(0)+J(\omega_{\mathrm{H}} - \omega_{\mathrm{N}})+3J(\omega_{\mathrm{N}}) + 6J(\omega_{\mathrm{H}}) + 6J(\omega_{\mathrm{H}} + \omega_{\mathrm{N}})\right] + \frac{(\rho\omega_{\mathrm{N}})^2}{90}\left[4J(0) + 3J(\omega_{\mathrm{N}})\right] \tag{2}$$

$$\mathrm{hetNOE} = 1 + \frac{d_{\mathrm{NH}}^2 N_{\mathrm{H}}}{20}\left[J(\omega_{\mathrm{H}} - \omega_{\mathrm{N}}) + 6J(\omega_{\mathrm{H}} + \omega_{\mathrm{N}})\right]\frac{\gamma_{\mathrm{H}} T_1}{\gamma_{\mathrm{N}}}, \tag{3}$$

Here, ω_{H} and ω_{N} are Larmor frequencies of ^{1}H and ^{15}N, respectively, $N_{\mathrm{H}} = 1$ is the number of protons in the N-H bond, and $\Delta\rho = -160$ ppm is the chemical shift anisotropy (56). The dipolar coupling constant is defined as $d_{\mathrm{NH}} = \frac{\mu_0 \hbar \gamma_{\mathrm{H}} \gamma_{\mathrm{N}}}{4\pi\langle r_{\mathrm{NH}^3}\rangle}$, where μ_0 is vacuum permeability, $\hbar$ is the reduced Planck constant, γ_{H} and γ_{N} are the gyromagnetic constants of ^{1}H and ^{15}N, respectively, and the average cubic length is calculated as $\langle r^3{}_{\mathrm{NH}}\rangle = (0.101\ \mathrm{nm})^3$.

Spectral density, $J(\omega)$, is the Fourier transformation of the N-H rotational correlation function

$$C(t) = \left\langle \frac{3}{2}\cos^2\theta_{t'+t} - \frac{1}{2}\right\rangle_{t'}, \tag{4}$$

where $\theta_{t'+t}$ is the angle between bond vector at the times t and t'. Spectral densities were calculated from MD simulations as described previously (25,29,48,55). Rotational correlation functions (Equation 4) for the N-H bonds were calculated using *gmx rotacf* from gromacs package (57). A sum of exponential functions

$$C_{\mathrm{fit}}(t) = \sum_{i=1}^{N} \alpha_i e^{-t/\tau_i}. \tag{5}$$

with the large number of prefixed timescales ($N = 100$, τ_i values were equidistantly spaced in logarithmic scale between 1 ps and 100 ns) was fitted to the correlation functions from simulations using the Python scipy optimize.nnls to solve the weight, α_i, for each timescale that represents the relevance of the given timescale for the rotational relaxation of the bond. Spectral densities were then calculated as

$$J(\omega) = 2\int_0^{\infty} C_{\mathrm{fit}}(t)cos(\omega t)\mathrm{d}t = 2\sum_{i=1}^{N}\alpha_i\frac{\tau_i}{1+\omega^2\tau_i^2} \tag{6}$$

Ranking of simulations and selection of best ensembles

Similarly to previous work on multidomain and disordered proteins (26,29), root mean-square deviations (RMSDs) between simulations and experiments were calculated for each spin relaxation rate by averaging over residues as

$$\mathrm{RMSD}_y = \sqrt{\frac{1}{n}\sum_{i=1}^{n}\left(y_i^{\mathrm{sim}} - y_i^{\mathrm{exp}}\right)^2}, \tag{7}$$

where y refers to a type of spin relaxation (R_1, R_2 or hetNOE), and y_i^{sim} and y_i^{exp} are its values from simulations and experiments, respectively. Summation goes over all residues with experimental data available, but four points with largest RMSD values were excluded to mitigate the potential effects of outliers. For each spin relaxation rate, comparison numbers were then defined by dividing the RMSD by the lowest RMSD observed for the given spin relaxation rate among all simulations of the given protein:

$$C_y[\mathrm{sim}] = \frac{\mathrm{RMSD}_y[\mathrm{sim}]}{\min\limits_{\mathrm{sim}}\mathrm{RMSD}_y[\mathrm{sim}]} \times 100\% \tag{8}$$

RMSDs and comparison numbers for peptides studied here are shown in Figs. S2, S9, S16, S24, S32, and S40.

In previous studies (26,29), simulations having comparison numbers below 150% simultaneously for all spin relaxation rates were selected for further analysis, or if none of the simulations satisfied this condition, the one with the lowest sum of comparison numbers for different spin relaxation rates was selected. However, experimental data for the short peptides studied here are available for only a few labeled amino acids, which may lead to suboptimal selections with the previously used criteria. For example, for $(\mathrm{GPS})_3$, replica 3 from DESAMBER simulations has two R_1 values very close to experimental data (Fig. S10), which leads to very low value for minimum RMSD among the simulations (denominator in Eq. 8), and therefore to large comparison numbers for all other simulations (Fig. S9),

although spin relaxation times would not be very far from experiments. Such situations are less likely for previously studied larger proteins with more spin relaxation time values available from many residues (26,29).

For this reason, here, we manually selected simulations that are in best overall agreement with experimental spin relaxation rates for each peptide. For $(GGS)_3$, all AMBER99SB-DISP simulations were selected because they were clearly in best overall agreement with experiments, as evident also from Fig. S2. For $(GPS)_3$, all AMBER99SB-DISP replicas were selected mainly due to their best performance for hetNOE values in Fig. S12, whereas other spin relaxation times are at least equally close to experiments as in other simulations in Figs S10 and S11. For, $K(AP)_5K$, DESAMBER and AMBER99SB-DISP had the best agreement with experiments for hetNOE in Fig. S19, but DESAMBER replicas 1, 2, and 4 were finally selected due to their better agreement with experimental R_2 values in Fig. S18. For the charged peptide, all AMBER03WS and CHARMM36M replicas were selected mainly due to their best performance for R_1 in Fig. S25, whereas other spin relaxation times are at least equally close to experiments as in other simulations in Figs. S26 and S27. The same systems were selected for charged simulations with added NaCl and $CaCl_2$ because the main goal was to investigate the effect of ions to the system.

Notably, differences between sequences are qualitatively similar for all force fields except CHARMM36M, for which looping back is not observed in $(GGS)_3$ simulations. Therefore, the main conclusions in this work are not sensitive for the exact selection of simulations for the final ensemble. This differs from previous studies for multidomain (29) and disordered proteins (26) where substantial differences in results between force fields and initial configurations were observed.

Characterization of the selected ensembles

All simulations and the ensembles selected by QEBSS protocol were characterized using the automatized version of QEBSS (26), which calculates and plots spin relaxation times against experiments, radius of gyration distributions, effective correlation times and timescales, overlayed snapshots, backbone correlation, and distance maps. Full details of analyses are described in Ref. (26). Briefly, radii of gyration distributions were calculated using the *gmx gyrate* from GROMACS 2024.1 package (49). Effective rotational correlation times of the backbone N-H bonds were calculated as weighted average over all timescales in a multiexponential decay fitted to N-H bond rotational correlation functions (58). For the calculation of spin relaxation times and effective correlation times, the average correlation functions over selected simulations were first calculated for each residue, and these correlation functions were used in the calculation. Error bars for the spin relaxation times, effective correlation times, and the backbone rigidity parameter, k, were calculated as the standard error of the mean over the corresponding values calculated separately from each QEBSS selected simulation using the SciPy Python library (59). Overlaid snapshots were generated by extracting each conformation every 10,000 frames (100 ns) from each replica for each of the five force fields using PyMOL (60). Backbone orientational correlation maps were calculated from dot products of vectors between consecutive C_α backbone atoms (25). Distance and contact maps are calculated as the pairwise average distances between backbone carbon alpha atoms from each trajectory, using MDAnalysis library (61). Furthermore, contact probabilities of calcium and sodium ions with the glutamic acid pairs of the charged peptide were calculated with a cutoff distance of 5.0 Å using MDAnalysis (61).

NMR experiments

Sample preparation

The ^{15}N isotopically labeled $(GPS)_3$, $(GGS)_3$, (glycines labeled in both sequences), $K(AP)_5K$ (alanines labeled), and the charged peptide (valines labeled) were purchased from Peptide Synthetics, Peptide Protein Research (https://www.peptidesynthetics.co.uk/) in powder form. For sequences and labeled residues, see Fig. 1 *a*. Approximately 0.3 mg of the powder peptide was weighted and dissolved to Milli-Q water with 5% of D_2O (to acquire high resolution spectra), which was buffered to pH 7.4 using 20 mM sodium phosphate. The final volume was 500 μL. The samples were then transferred to 5-mm NMR tubes. For experiments with charged peptide and monovalent ions, powder was dissolved with 150 mM NaCl and 20 mM of sodium phosphate buffer at pH 7.4. For experiments with divalent ions, powder was dissolved with 10mM $CaCl_2$ and 20mM of Tris-HCl buffer at pH 7.5 because $CaCl_2$ would precipitate sodium phosphate.

NMR measurements

NMR measurements were performed in the Bruker Avance IIIHD 850 MHz equipped with a cryogenically cooled probe head at the Institute of Biotechnology, University of Helsinki. [^1H,^{15}N]-HSQC and spin relaxation times T_1 and T_2 and hetNOE were collected with standard pulse sequences (62) at 298 K. The series of delay times are the following for T_1: 20, 50, 100, 200, 300, 500, 700, 900, 1500 and 1900 ms and T_2: 1, 2, 4, 8, 10, 16, 20, 24, 28, 32 ms. Recycle delays were 2.5 s and 3.5 s for T_1 and T_2 respectively. To detect the potential chemical exchange contribution, CPMG refocusing pulses (63) were applied with the CPMG frequencies of 50, 100, 200, 250, 500, 750, 1000, 1500, and 2000 Hz. T_1 and T_2 experiments were processed and analyzed using Bruker Dynamic Center software (version 2.7.2). For the analysis of hetNOE, peak intensities were determined by TopSpin software (version 4.3.0) from spectra with saturated hydrogens and the reference spectrum. Error bars are included in the graphs but are smaller than the size of the data points.

Peak assignment

For $(GGS)_3$, we observed five peaks in the HSQC spectra as expected based on the labeling (Fig. S1). The glycines numbered 4,5 and 7,8 were assigned to the peaks that are in very close vicinity in the spectra (Fig. S1) due to their similar chemical environment in the primary sequence. Final assignment was selected to provide optimal agreement with spin relaxation times from MD simulations.

The spectra of $(GPS)_3$ had two peaks with high intensity as expected, but also some additional peaks with lower intensity. We interpret the additional smaller peaks to correspond the proline *cis*-isomers with lower abundance than *trans*-isomers that result in higher intensity peaks (64,65). The high intensity peaks of GPS were assigned to the glycines 4 and 7 as shown in Fig. S1. The selected assignment provided the best agreement between simulations and experiments, but swapping the assignment would not change the conclusions of this work.

For $K(AP)_5K$, we observed three high intensity peaks of which one had distinctly higher intensity than the others. In addition to the main peaks, peaks with lower intensities were observed and interpreted to arise from proline *cis*-isomers (64,65) similarly to the $(GPS)_3$. Since the three alanines in the middle of the sequence (4,6,8) are surrounded by similar amino acids (either alanine or proline), the highest observed peak was assigned to be overlapping peak from these three alanines in the middle of the sequence (Fig. S1). The other two alanines (2 and 10) were assigned to remaining high-intensity peaks as in Fig. S1. Similarly to $(GPS)_3$, the selected assignment provided the best agreement between simulations and experiments, but swapping the assignment would not change the conclusions of this work.

For the KKEEVKKEEV$(PK)_7$KEEVKKEEVKK peptide, we observed four distinct peaks as expected based on the labeling (Fig. S1). These were assigned as shown in Fig. S1 because such assignment resulted in good agreement between simulations and experiments. Alternative assignments were also tested, but conclusions in this work were not affected.

RESULTS AND DISCUSSION

Spin relaxation measurements reveal sequence-dependent backbone dynamics

The selected peptides were first characterized experimentally by measuring T_1, T_2, and hetNOE from selectively ^{15}N-labeled backbone residues shown in Fig. 1 *a*. Assignments of labeled residues in ^1H-^{15}N HSQC spectra are

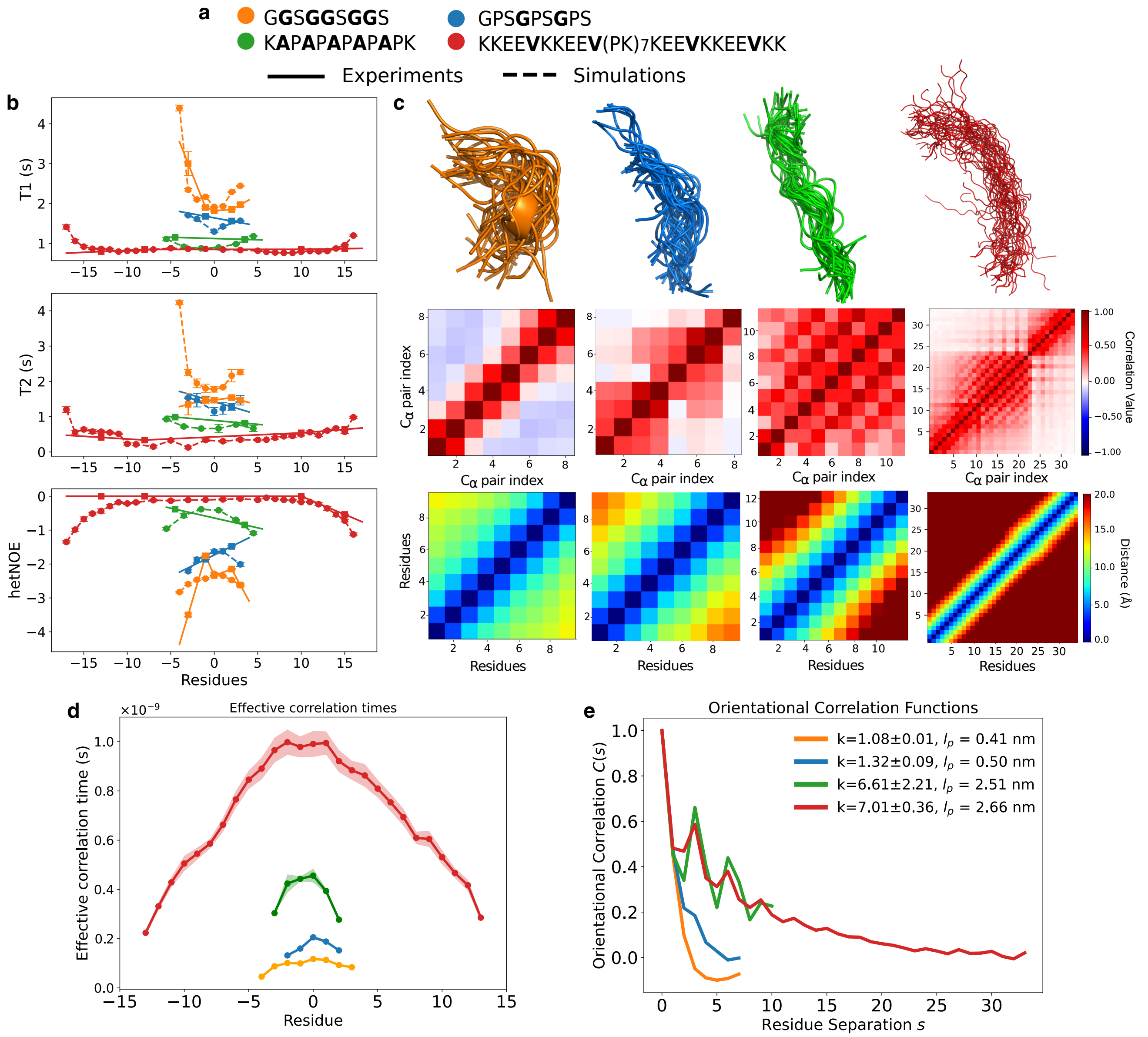

FIGURE 1 Characterization of studied peptides. (*a*) Amino acid sequences of the studied peptides with ^{15}N-labeled residues shown in bold: $(GGS)_3$ = G**G**S**GG**S**GG**S, $(GPS)_3$ = GPS**G**PS**G**PS, $K(AP)_5K$ = K**A**P**A**P**A**P**A**P**A**PK, and KKEE**V**KKEE**V**$(PK)_7$KEE**V**KKEE**V**KK = "charged sequence." (*b*) Spin relaxation times from experiments and the selected ensembles. Origin of x-axis is set to the center of the sequence. Spin relaxation times from MD simulations for first residues in sequences are not shown for clarity because they exhibit very fast dynamics leading to long times. (*c*) Representative snapshots, backbone orientation correlation maps, and distance maps from QEBSS ensembles. (*d*) Effective correlation time plots for the selected simulations for each disordered linker. (*e*) Orientational correlation functions as function of the pairwise residue sequence separation, *s*, along with the approximate persistence lengths, l_p. Errors in (*b*), (*d*), and (*e*) were calculated from the standard error of the mean when averaging over QEBSS selected simulations.

shown in Fig. S1. Spectra from $(GGS)_3$ and charged peptide have five and four distinct peaks corresponding to the labeled glycines and valines, respectively. However, the spectra of $(GPS)_3$ and $K(AP)_5K$ exhibit additional peaks with lower intensities. The additional peaks can be explained by the presence of prolines in their less abundant *cis* isomer conformation (64–66). Because only larger peaks are used in further analyses, our results apply only for chains containing prolines as *trans*-isomers. Conformational exchange was not observed for any of the peptides in the CPMG relaxation dispersion experiments.

Systematic comparison of experimental spin relaxation times T_1 and T_2 across the sequences reveals a decreasing trend in the order $(GGS)_3$ > $(GPS)_3$ > $K(AP)_5K$ > KKEE VKKEEV$(PK)_7$KEEVKKEEVKK, whereas hetNOE exhibits similar systematic trends but in the opposite direction (Fig. 1 *b*). The decreasing T_2 values indicate a slowdown in backbone rotational dynamics (58), whereas the increasing

hetNOE values reflect enhanced protein order (58,67). However, a more detailed physical interpretation of these experimental trends requires additional theoretical models (68).

Molecular dynamics simulations reveal a correlation between backbone rigidity and spin relaxation times

To interpret the experimentally observed differences between the linker sequences in more detail, we used the tailored QEBSS approach to create conformational ensembles based on MD simulations that reproduce the experimental spin relaxation data. The pool of MD simulations is first created by running simulations with five different initial configurations and force fields (AMBER99SB-DISP (23), DESAMBER (46), AMBER99SBWS (38), AMBER03WS (38) and CHARMM36M (43)) resulting in 25 simulations for each sequence. Spin relaxation times calculated from simulations are then compared with the experimental ones, and this comparison is used to select the ensemble that best describes the experimental data for each sequence.

Because ensembles resulting from the QEBSS approach reproduce the experimentally observed spin relaxation time differences between sequences (Fig. 1 *b*), we use them to provide interpretation for experimentally observed trends. Peptide backbone stiffness described by the backbone orientational correlations in Fig. 1 *c* follows the same trend as T_1 and T_2 spin relaxation times and increases in the order of $(GGS)_3 < (GPS)_3 < K(AP)_5K <$ KKEEVKKEEV$(PK)_7$KEEV KKEEVKK, suggesting that the backbone ^{15}N spin relaxation times are sensitive to the backbone rigidity. Ranking tables, comparison of spin relaxation rates with experiments, contact maps, and radius of gyration distributions are shown for all created simulations and peptides in Figs. S2–S46.

More detailed analysis reveals essential differences in peptide characteristics between different sequences. For glycine-rich $(GGS)_3$ peptide, backbone orientational correlation values decrease to negative values after separation of two residues (Fig. 1 *c* and *e*), suggesting a slight tendency of the protein chain to loop back. When prolines are introduced into the sequence, the peptide adopts more open conformations having positive backbone correlations observed throughout the $(GPS)_3$ sequence. This effect is even more pronounced in $K(AP)_5K$ and the charged sequence with higher proline percentages. Our results are well aligned with previous studies where looping back of glycine-rich sequences (69,70) and straightening due to prolines (70,71) are reported. Moreover, our results demonstrate that the backbone rigidity of disordered peptides, associated with the backbone orientational correlations, can be characterized by combining NMR experiments and MD simulations.

For a disordered protein behaving like an ideal freely jointed polymer chain, rigidity could be described by the backbone orientational correlation function $C(s) = \langle \boldsymbol{n}_i \times \boldsymbol{n}_{i+s} \rangle = e^{-s/k}$, where $\boldsymbol{n}_i$ is the unit vector between adjacent alpha carbon atoms, s is the separation distance, and decay constant is related to the persistence length as $l_p = k \times 0.38$ nm (22). Larger l_p values indicate stiffer protein backbones and vice versa. However, backbone orientational correlation functions in Fig. 1 *e* do not follow clear exponential behavior, suggesting that persistence length and other simple polymer theory concepts are not sufficient to fully characterize the studied peptides. For $(GGS)_3$, the looping back of the protein chain leads to a rapid decay to negative values. Other peptides exhibit a rapid decrease up to the second residue, followed by a small rebound at the third residue, indicating a short-range ordering in the backbone orientation that leads to the nonexponential decay. Similar behavior was observed also for some disordered proteins (26). Nevertheless, backbone rigidities approximated by l_p values, acquired by fitting the correlation function C(s) to the exponential, are shown in Fig. 1 *e*. The range of resulting approximations for persistence lengths, 0.4–2.7 nm, span the previously reported values for disordered proteins in the literature, 0.3–2.6 nm (16–20,22,72).

Increasing backbone rigidity with proline content also correlates with the slower average rotational timescales of peptides described by effective correlation times (Fig. 1 *d*), in line with the literature (73). However, the relatively large difference in effective correlation times between $K(AP)_5K$ and the charged peptide (Fig. 1 *d*) suggests that also increasing peptide length seems to slow down the backbone rotational dynamics. This is somewhat reflected also in the spin relaxation times as the $K(AP)_5K$ and the charged peptide have approximately similar rigidity in terms of persistence length, but slightly different spin relaxation times (Fig. 1). Although our results suggest that backbone rigidity would largely determine the spin relaxation times of at least short, disordered peptides, the possible effects of peptide length should be considered when interpreting such results.

Force field evaluation reveals varying performance across peptide sequences

In addition to interpreting experimental data, our results provide new insights into the ability of MD simulation models to accurately capture the experimental behavior of disordered proteins. Applications of MD simulations to characterize disordered proteins are often limited by the accuracy of the force fields and slow convergence of conformational sampling with respect to feasible trajectory lengths (23–27). QEBSS approach is designed to circumvent these issues by generating a diverse set of ensembles with multiple force fields and initial configurations (26,29). MD simulation results for two-domain and disordered proteins in previous studies show significant dependence on initial configurations (26,29). Here, we observed converged results from independent initial configurations

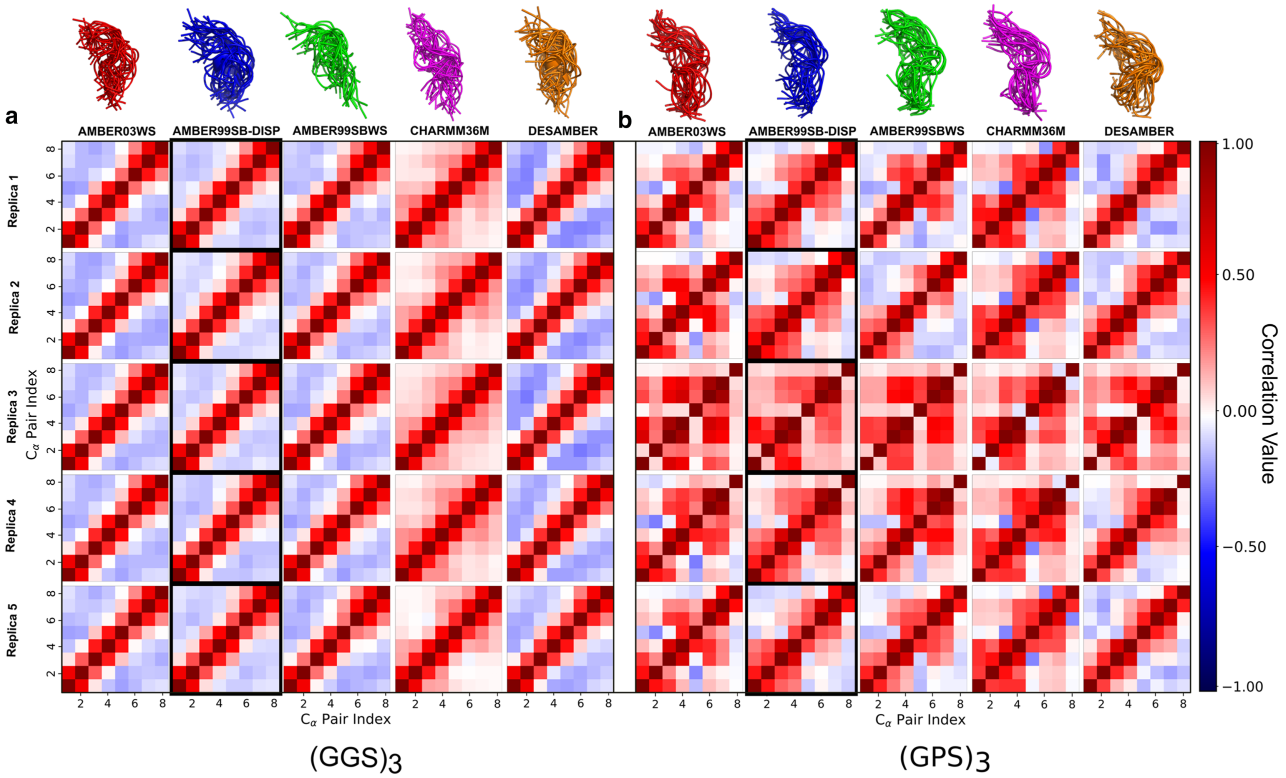

FIGURE 2 Backbone orientation correlation maps from QEBSS simulations with five different initial structures and force fields. (*a*) $(GGS)_3$ and (*b*) $(GPS)_3$. Selected simulations are highlighted with black squares.

for $(GGS)_3$ (Figs. 2 *a* and S3–S7), suggesting that conformational ensembles from different force fields are equilibrated for this peptide. However, conformational ensembles of other peptides studied here somewhat depend on initial configurations (Figs. 2 *b*, S20, S28, S36, and S44), suggesting that simulation times of 1–2 μs were not sufficient to generate fully equilibrated ensembles even for short peptides with proline residues. Slower conformational sampling of proline than glycine-containing sequences has been previously reported in triplet-triplet energy transfer experiments (73,74). These results indicate that times required for full conformational space sampling depend on amino acid content in addition to the length of a sequence. Further studies are needed to determine how long simulations are required for conformational sampling of disordered proteins with different sequences.

The converged results for $(GGS)_3$ enable us to rank the quality of force fields against experimental data for this peptide. The ranking from best to worst is AMBER99SB-DISP, DESAMBER, AMBER99SBWS, AMBER03WS, and CHARMM36M (Fig. S2). Ranking of force field qualities for other peptides is less straightforward because results depend on initial configurations. Even stronger dependencies on initial configurations were observed in previous studies for two-domain and disordered proteins (26,29). Nevertheless, useful trends indicating performance of force fields for different sequences can be derived from the results. AMBER99SB-DISP is the best performing force field for most flexible peptides, $(GGS)_3$ and $(GPS)_3$. DESAMBER simulations are selected for $K(AP)_5K$, and AMBER03WS and CHARMM36M for the charged peptide. The only force field that is not among the selected simulations for any of the peptides studied in this work is AMBER99SBWS. However, it gave the most realistic ensemble for one of the previously studied disordered proteins (26), advocating its value in generating diverse ensembles in QEBSS approach even though it was not selected for peptides in this work.

Despite some systematic differences in results between different force field parameters, all used force fields predict qualitatively similar backbone rigidities for individual peptides and differences between them—except CHARMM36M, which predicts a more extended configuration for $(GGS)_3$, lacking the looping-back behavior observed with other force fields. These results suggest that current force fields designated for intrinsically disordered proteins (IDPs) can qualitatively reproduce differences between backbone rigidities of short sequences lacking significant intrachain interactions. However, for IDPs with substantial intrachain interactions, simulation predictions depend on the specific force field used, and variations in backbone rigidity with sequence composition are not accurately predicted (26). Our findings are consistent with previous results that suggest that protein-protein and protein-water interactions require refinement in current force field parameters because they

significantly affect intrachain interactions, whereas local backbone rigidities are better described by existing parameters (24).

Ion binding minimally affects backbone rigidity of charged peptides

Beyond amino acid sequence, the conformational ensembles and backbone rigidities of IDPs containing charged residues are expected to depend on ion concentration in the surrounding solvent (25,75). To characterize these effects, we investigated the charged peptide in the presence of 150 mM NaCl and 10 mM $CaCl_2$. Changes in labeled valine peak positions in HSQC spectra upon addition of NaCl and $CaCl_2$ indicate that ions interact with the peptide termini where valines are located (Fig. 3 *e*). Changes in spin relaxation times due to ion addition are also observed (Fig. 3 *b*); however, these changes are neither systematic nor substantial, being significantly smaller than the differences observed between different amino acid sequences in Fig. 1.

Ion binding affinity in MD simulations strongly depends on the force field parameters employed (Fig. S48). The best force fields for charged peptides based on ranking against experimental spin relaxation times, AMBER03WS and CHARMM36M, predict substantially stronger binding for calcium ions compared with sodium ions (Fig. 3 *d*). These simulations qualitatively reproduce the experimentally observed differences in spin relaxation times between NaCl and $CaCl_2$ solutions, though with slight overestimation (Fig. 3 *b*). In contrast, AMBER99SB-DISP and DESAMBER force fields predict approximately equal ion binding probabilities for 150 mM NaCl and 10 mM $CaCl_2$ and do not capture experimental differences in spin relaxation times (Fig. S49). Based on these results, the larger binding affinity differences between sodium and calcium ions in AMBER03WS and CHARMM36M simulations (Fig. 3 *e*) appear slightly overestimated when compared with experiments but more realistic than the smaller differences observed in AMBER99SB-DISP and DESAMBER simulations (Fig. S49).

However, changes in spin relaxation times upon addition of ions are not fully systematic in experiments as the hetNOE values with NaCl deviated more from the values without additional than $CaCl_2$ result despite the presumable stronger ion binding in the latter (Fig. 3 *b*). Considering this and the relatively small ion-induced changes observed, more systematic experimental data measured across different ion concentrations are required for definitive conclusions regarding ion binding affinities to IDPs. Nevertheless, our results suggest that carefully designed spin relaxation experiments with systematically varying ion concentration have potential to provide highly valuable experimental NMR data for evaluating ion binding to IDPs in different simulation models, analogous to lipid studies where lipid headgroup order parameters have been utilized (76).

MD simulations in best agreement with the spin relaxation data suggest slight backbone stiffening upon $CaCl_2$ addition (Fig. 3 *c*). However, changes in spin relaxation times and peptide biophysical characteristics upon ion addition are relatively modest even with high amounts of bound ions. Although contact probabilities for calcium exceed 0.6 (Fig. 3 *e*), the observed differences in peptide behavior remain relatively small (Fig. 3 *b* and *c*). Together with another study reporting unexpectedly weak salt concentration dependence (19), our results suggest that electrostatics may not be the dominant factor governing backbone rigidity even for highly charged proteins, as evidenced by the negligible effect of ion binding on rigidity and spin relaxation times.

CONCLUSIONS

Our results indicate that peptide backbone ^{15}N spin relaxation times correlate with the backbone rigidity of disordered linkers. Therefore, NMR spin relaxation data and ensembles resolved with QEBSS (26,29) are useful in characterization of IDP backbone rigidities. This combination of techniques is particularly valuable for probing differences in the properties of short peptides that are difficult to resolve with many experimental approaches commonly paired with MD simulations for characterizing IDPs (77,78). Scattering and paramagnetic relaxation enhancement (PRE) measurements often lack sensitivity for very small peptides, whereas chemical shifts respond more readily to changes in the local chemical environment than to backbone rigidity.

Backbone rigidity shows substantial dependence on amino acid sequence, particularly increasing upon addition of prolines, but it exhibits no significant correlation with ion solution conditions. These results are in line with previous studies (19,69–71), yet our approach provides more detailed and quantitative analysis on backbone rigidity.

Protein backbone rigidity regulates many important biological and biotechnological functions of disordered linkers, including their effective length, conformational flexibility, and accessibility of amino acids for intermolecular interactions. The characterization capabilities demonstrated here open new avenues for more detailed understanding of molecular biology and enable more efficient protein design and engineering strategies.

Furthermore, our results provide valuable insights into the performance of different MD simulation force fields for disordered linkers under various solution conditions. This information facilitates the selection of optimal parameters for specific applications, guides the development of improved force fields, and supports the usage of MD simulation data for the training of machine learning models capable of predicting linker properties from sequence alone. Finally, the synergistic approach combining NMR experimental data with MD simulations provides a powerful framework for interpreting complex experimental observations that would otherwise be difficult to understand in terms of underlying MD and structural behavior.

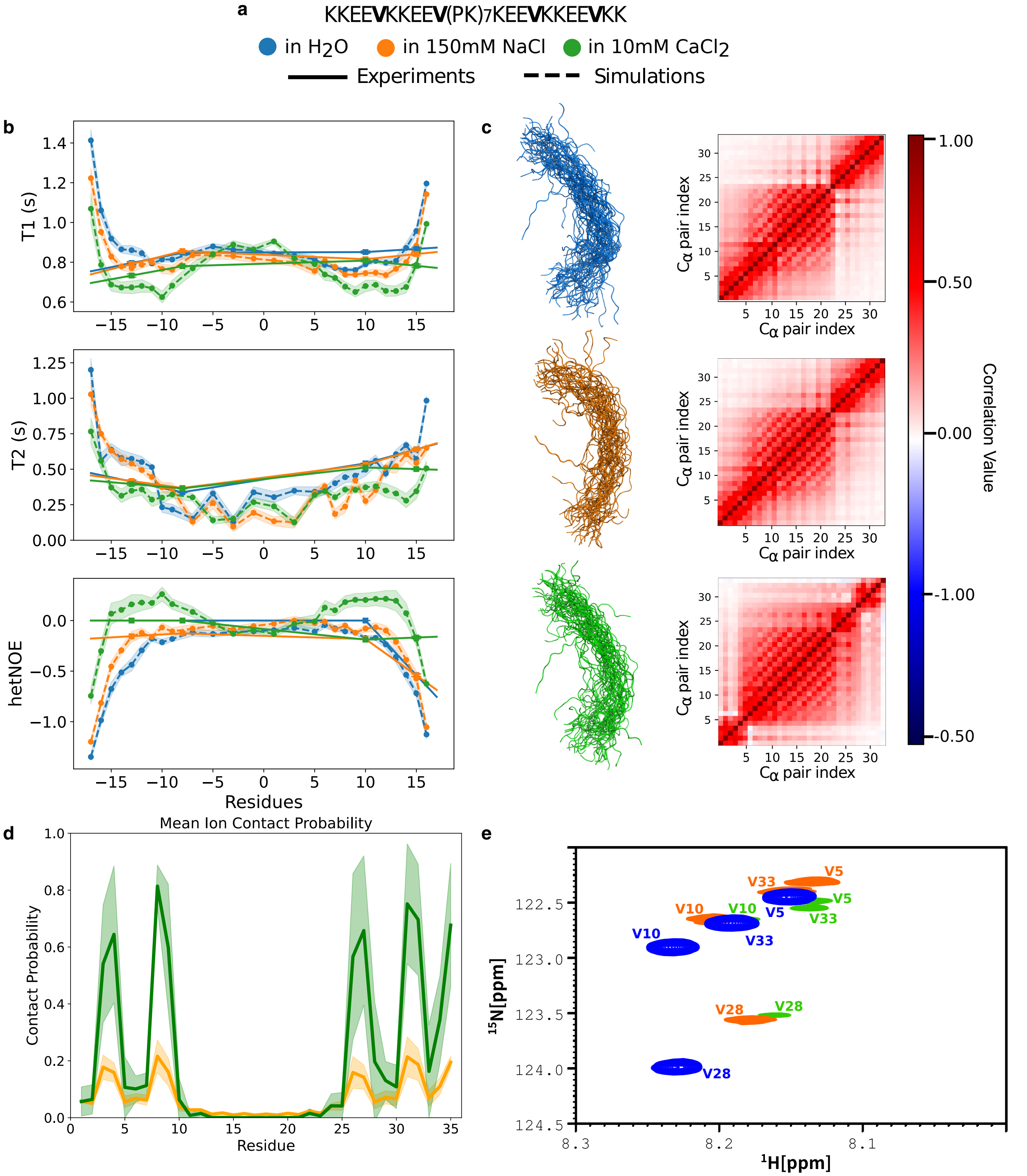

FIGURE 3 Effect of ion interactions on studied peptides. (*a*) Amino acid sequence of the charged peptide studied with different ion concentrations. ^{15}N-labeled residues are shown in bold. (*b*) Spin relaxation times from experiments and the selected ensembles. Spin relaxation times from MD simulations for first residues in sequences are not shown for clarity because they exhibit very fast dynamics leading to long times. (*c*) Representative snapshots and backbone orientation correlation maps from QEBSS ensembles. (*d*) Average contact probability of calcium and sodium ions with the glutamic acid pairs of the charged peptide over the selected ensembles. (*e*) Overlaying HSQC spectra for the charged peptide under 150 mM NaCl and 10m M $CaCl_2$. Errors in (*b*) and (*d*) were calculated from the standard error of the mean when averaging over QEBSS selected simulations.

DATA AND CODE AVAILABILITY

All simulation trajectories and relevant files for their reproduction are available in Zenodo (https://zenodo.org/) repositories that are listed in Table S1. The scripts used for preparation, running and analysis of the simulations are available at https://github.com/vttresearch/QEBSS/.

ACKNOWLEDGMENTS

We gratefully acknowledge the facilities and expertise of the HiLIFE NMR unit at the University of Helsinki, a member of Instruct-ERIC Center Finland, FINStruct, and Biocenter Finland. We acknowledge CSC – IT Center for Science for computational resources and the Research Council of Finland for funding (grant nos. 315596, 356568, and 350636). R.N. acknowledges funding from Emil Aaltonen Foundation. E.M. acknowledges funding from the Ministry of Education and Culture's Doctoral Education Pilot under Decision no. VN/3137/2024-OKM-6 (Circular Materials Bioeconomy Network, CIMANET).

AUTHOR CONTRIBUTIONS

E.M. performed all simulations included in the article and their analyses, prepared samples, performed and analyzed NMR experiments, made all figures, and wrote the first version of the manuscript. C.K.M. developed the automatized QEBSS protocol, supported in the analysis of simulations, and commented on the manuscript. R.N. supervised NMR experiments, supported in analysis of simulations and NMR experiments, and commented on the manuscript. A.E.S. performed preliminary simulations that supported the conceptualization of the work and commented on the manuscript. O.H.S.O. conceptualized and supervised the work and wrote the article together with other authors.

DECLARATION OF INTERESTS

The authors declare no competing financial interests or personal relationships that could have appeared to influence the work reported in this paper.

DECLARATION OF GENERATIVE AI AND AI-ASSISTED TECHNOLOGIES IN THE WRITING PROCESS

During the preparation of this work the authors used Claude (Anthropic) in order to polish the text. After using this service, the authors reviewed and edited the content as needed and take full responsibility for the content of the published article.

SUPPORTING MATERIAL

Supporting material can be found online at https://doi.org/10.1016/j.bpj.2026.03.009.

REFERENCES

1. Vogel, C., M. Bashton, …, S. A. Teichmann. 2004. Structure, function and evolution of multidomain proteins. *Curr. Opin. Struct. Biol.* 14:208–216.
2. Hilditch, A. T., A. Romanyuk, …, D. N. Woolfson. 2024. Assembling membraneless organelles from de novo designed proteins. *Nat. Chem.* 16:89–97.
3. Mahmood, T., A. Shahbaz, …, K. Rizwan. 2023. Recent Advancements in Fusion Protein Technologies in Oncotherapy: A Review. *Int. J. Biol. Macromol.* 230:123161.
4. Zhang, L., J. R. Lu, and T. A. Waigh. 2021. Electronics of Peptide- and Protein-Based Biomaterials. *Adv. Colloid Interface Sci.* 287:102319.
5. Mendes, G. G., B. Faulk, …, S. E. Bondos. 2024. Genetic Functionalization of Protein-Based Biomaterials via Protein Fusions. *Biomacromolecules.* 25:4639–4662.
6. Gan, S. Y., G. J. Tye, …, N. S. Lai. 2023. Linker-Mediated Oriented Antibody Immobilisation Strategies for a More Efficient Immunosensor and Diagnostic Applications: A Review. *Biosens. Bioelectron. X.* 14:100379.
7. Hannula, L., S. Kuivanen, …, I. Rissanen. 2024. Nanobody Engineering for SARS-CoV-2 Neutralization and Detection. *Microbiol. Spectr.* 12:e0419922.
8. Liu, S., and W. Li. 2022. Protein Fusion Strategies for Membrane Protein Stabilization and Crystal Structure Determination. *Crystals.* 12:1041.
9. Chen, X., J. L. Zaro, and W.-C. Shen. 2013. Fusion protein linkers: Property, design and functionality. *Adv. Drug Deliv. Rev.* 65:1357–1369.
10. Papaleo, E., G. Saladino, …, R. Nussinov. 2016. The Role of Protein Loops and Linkers in Conformational Dynamics and Allostery. *Chem. Rev.* 116:6391–6423.
11. Gräwe, A., and V. Stein. 2021. Linker Engineering in the Context of Synthetic Protein Switches and Sensors. *Trends Biotechnol.* 39:731–744.
12. Jeong, W. H., H. Lee, …, J.-O. Lee. 2016. Connecting Two Proteins Using a Fusion Alpha Helix Stabilized by a Chemical Cross Linker. *Nat. Commun.* 7:11031.
13. Collu, G., T. Bierig, …, R. M. Benoit. 2022. Chimeric Single α-Helical Domains as Rigid Fusion Protein Connections for Protein Nanotechnology and Structural Biology. *Structure.* 30:95–106.e7.
14. Patel, D. K., D. Vettiatil, …, G. Dave. 2022. Linkers: A Synergistic Way for the Synthesis of Chimeric Proteins. *Protein Expr. Purif.* 191:106012.
15. Maiti, S., A. Singh, …, S. De. 2024. Experimental Methods to Study the Structure and Dynamics of Intrinsically Disordered Regions in Proteins. *Curr. Res. Struct. Biol.* 7:100138.
16. Evers, T. H., E. M. W. M. van Dongen, …, M. Merkx. 2006. Quantitative Understanding of the Energy Transfer between Fluorescent Proteins Connected via Flexible Peptide Linkers. *Biochemistry.* 45:13183–13192.
17. van Rosmalen, M., M. Krom, and M. Merkx. 2017. Tuning the Flexibility of Glycine-Serine Linkers To Allow Rational Design of Multidomain Proteins. *Biochemistry.* 56:6565–6574.
18. Hofmann, H., A. Soranno, …, B. Schuler. 2012. Polymer scaling laws of unfolded and intrinsically disordered proteins quantified with single-molecule spectroscopy. *Proc. Natl. Acad. Sci. USA.* 109:16155–16160.
19. Chin, A. F., D. Toptygin, …, V. J. Hilser. 2016. Phosphorylation Increases Persistence Length and End-to-End Distance of a Segment of Tau Protein. *Biophys. J.* 110:362–371.
20. Zhou, H.-X. 2004. Polymer Models of Protein Stability, Folding, and Interactions. *Biochemistry.* 43:2141–2154.
21. Cheng, S., M. Cetinkaya, and F. Gräter. 2010. How Sequence Determines Elasticity of Disordered Proteins. *Biophys. J.* 99:3863–3869.
22. Shrestha, U. R., J. C. Smith, and L. Petridis. 2021. Full structural ensembles of intrinsically disordered proteins from unbiased molecular dynamics simulations. *Commun. Biol.* 4:243.
23. Robustelli, P., S. Piana, and D. E. Shaw. 2018. Developing a molecular dynamics force field for both folded and disordered protein states. *Proc. Natl. Acad. Sci. USA.* 115:E4758–E4766.
24. Zerze, G. H., W. Zheng, …, J. Mittal. 2019. Evolution of All-Atom Protein Force Fields to Improve Local and Global Properties. *J. Phys. Chem. Lett.* 10:2227–2234.
25. Virtanen, S. I., A. M. Kiirikki, …, O. H. S. Ollila. 2020. Heterogeneous dynamics in partially disordered proteins. *Phys. Chem. Chem. Phys.* 22:21185–21196.

26. Malm, C., M. Girych, …, O. H. S. Ollila. 2025. NMR spin relaxation enables rigorous selection of realistic intrinsically disordered protein ensembles from molecular dynamics simulations. *Preprint at ChemRxiv.* https://doi.org/10.26434/chemrxiv-2025-m5m0p.
27. Rindfleisch, T., R. Nencini, …, A. Thalhammer. 2025. Molecular dynamics of the Intrinsically disordered protein COR15A—A force field validation on structure and dynamics. *J. Chem. Theory Comput.* 21:9147–9163.
28. Henriques, J., C. Cragnell, and M. Skepö. 2015. Molecular Dynamics Simulations of Intrinsically Disordered Proteins: Force Field Evaluation and Comparison with Experiment. *J. Chem. Theory Comput.* 11:3420–3431.
29. Sandelin, A. E., R. Nencini, …, O. H. S. Ollila. 2025. Quality Evaluation Based Simulation Selection (QEBSS) for analysis of conformational ensembles and dynamics of multidomain proteins. *Commun. Chem.* 8:241.
30. Chen, H., Z. Chen, …, T. Tan. 2017. Influences of Various Peptide Linkers on the Thermotoga Maritima MSB8 Nitrilase Displayed on the Spore Surface of Bacillus Subtilis. *J. Mol. Microbiol. Biotechnol.* 27:64–71.
31. Silacci, M., N. Baenziger-Tobler, …, D. Grabulovski. 2014. Linker Length Matters, Fynomer-Fc Fusion with an Optimized Linker Displaying Picomolar IL-17A Inhibition Potency. *J. Biol. Chem.* 289:14392–14398.
32. Theillet, F.-X., L. Kalmar, …, V. N. Uversky. 2013. The alphabet of intrinsic disorder. *Intrinsically Disord. Proteins.* 1. https://doi.org/10.4161/idp.24360.
33. Breibeck, J., and A. Skerra. 2018. The Polypeptide Biophysics of Proline/Alanine-Rich Sequences (PAS): Recombinant Biopolymers with PEG-like Properties. *Biopolymers.* 109:e23069.
34. Celia, H., N. Noinaj, and S. K. Buchanan. 2020. Structure and stoichiometry of the ton molecular motor. *Int. J. Mol. Sci.* 21:375.
35. López, A., A. R. Elimelech, …, M. Sattler. 2021. The Charged Linker Modulates the Conformations and Molecular Interactions of Hsp90. *Chembiochem.* 22:1084–1092.
36. Teixeira, J. M. C., Z. H. Liu, …, J. D. Forman-Kay. 2022. A Flexible Software Suite for Sampling the Conformational Space of Disordered Protein States. *J. Phys. Chem. A.* 126:5985–6003.
37. Wang, G., and R. L. Dunbrack, Jr. 2003. PISCES: a protein sequence culling server. *Bioinformatics.* 19:1589–1591.
38. Best, R. B., W. Zheng, and J. Mittal. 2014. Balanced Protein–Water Interactions Improve Properties of Disordered Proteins and Non-Specific Protein Association. *J. Chem. Theory Comput.* 10:5113–5124.
39. Abascal, J. L. F., and C. Vega. 2005. A general purpose model for the condensed phases of water: TIP4P/2005. *J. Chem. Phys.* 123:234505.
40. Best, R. B., and J. Mittal. 2010. Balance between α and β Structures in Ab Initio Protein Folding. *J. Phys. Chem. B.* 114:8790–8798.
41. Best, R. B., and G. Hummer. 2009. Optimized Molecular Dynamics Force Fields Applied to the Helix-Coil Transition of Polypeptides. *J. Phys. Chem. B.* 113:9004–9015.
42. Best, R. B., D. de Sancho, and J. Mittal. 2012. Residue-Specific α-Helix Propensities from Molecular Simulation. *Biophys. J.* 102:1462–1467.
43. Huang, J., S. Rauscher, …, A. D. MacKerell. 2017. CHARMM36m: an improved force field for folded and intrinsically disordered proteins. *Nat. Methods.* 14:71–73.
44. Jorgensen, W. L., J. Chandrasekhar, …, M. L. Klein. 1983. Comparison of simple potential functions for simulating liquid water. *J. Chem. Phys.* 79:926–935.
45. Best, R. B., X. Zhu, …, A. D. Mackerell, Jr. 2012. Optimization of the Additive CHARMM All-Atom Protein Force Field Targeting Improved Sampling of the Backbone ϕ, ψ and Side-Chain $\chi1$ and $\chi2$ Dihedral Angles. *J. Chem. Theory Comput.* 8:3257–3273.
46. Piana, S., P. Robustelli, …, D. E. Shaw. 2020. Development of a Force Field for the Simulation of Single-Chain Proteins and Protein–Protein Complexes. *J. Chem. Theory Comput.* 16:2494–2507.
47. Piana, S., A. G. Donchev, …, D. E. Shaw. 2015. Water dispersion interactions strongly influence simulated structural properties of disordered protein states. *J. Phys. Chem. B.* 119:5113–5123.
48. Ollila, O. H. S., H. A. Heikkinen, and H. Iwaï. 2018. Rotational dynamics of proteins from spin relaxation times and molecular dynamics simulations. *J. Phys. Chem. B.* 122:6559–6569.
49. Abraham, M. 2024. GROMACS 2024.4 Manual.
50. Bussi, G., D. Donadio, and M. Parrinello. 2007. Canonical Sampling through Velocity Rescaling. *J. Chem. Phys.* 126:014101.
51. Parrinello, M., and A. Rahman. 1981. Polymorphic Transitions in Single Crystals: A New Molecular Dynamics Method. *J. Appl. Phys.* 52:7182–7190.
52. Darden, T., D. York, and L. Pedersen. 1993. Particle mesh Ewald: An N. log (N) method for Ewald sums in large systems. *The Journal of chemical physics.* 98:10089–10092.
53. Abragam, A. 1961. The Principles of Nuclear Magnetism. Oxford University Press.
54. Kay, L. E., D. A. Torchia, and A. Bax. 1989. Backbone Dynamics of Proteins as Studied by Nitrogen-15 Inverse Detected Heteronuclear NMR Spectroscopy: Application to Staphylococcal Nuclease. *Biochemistry.* 28:8972–8979.
55. Nencini, R., M. L. G. Regnier, …, O. H. S. Ollila. 2024. Probing the dynamic landscape of peptides in molecular assemblies by synergized NMR experiments and MD simulations. *Commun. Chem.* 7:28.
56. Hiyama, Y., C. H. Niu, …, D. A. Torchia. 1988. Determination of 15N chemical shift tensor via 15N-2H dipolar coupling in Boc-glycylglycyl[15N glycine]benzyl ester. *J. Am. Chem. Soc.* 110:2378–2383.
57. Abraham, M. J., T. Murtola, …, E. Lindahl. 2015. GROMACS: High performance molecular simulations through multi-level parallelism from laptops to supercomputers. *SoftwareX.* 1–2:19–25.
58. Nencini, R., E. Mantzari, …, O. H. S. Ollila. 2024. Rapid Interpretation of Protein Backbone Rotation Dynamics Directly from Spin Relaxation Data. *J. Phys. Chem. Lett.* 15:10204–10209.
59. Virtanen, P., and R. Gommers. 2020. SciPy 1.0: Fundamental Algorithms for Scientific Computing in Python. *Nat. Methods.* 17:261–272.
60. Schrödinger, L.; DeLano, W. PyMOL. http://www.pymol.org/pymol.
61. Michaud-Agrawal, N., E. J. Denning, …, O. Beckstein. 2011. MDAnalysis: A Toolkit for the Analysis of Molecular Dynamics Simulations. *J. Comput. Chem.* 32:2319–2327.
62. Barbato, G., M. Ikura, …, A. Bax. 1992. Backbone Dynamics of Calmodulin Studied by Nitrogen-15 Relaxation Using Inverse Detected Two-Dimensional NMR Spectroscopy: The Central Helix Is Flexible. *Biochemistry.* 31:5269–5278.
63. Long, D., M. Liu, and D. Yang. 2008. Accurately Probing Slow Motions on Millisecond Timescales with a Robust NMR Relaxation Experiment. *J. Am. Chem. Soc.* 130:17629.
64. Sebák, F., J. Szolomájer, …, A. Bodor. 2023. Proline Cis/Trans Isomerization in Intrinsically Disordered Proteins and Peptides. *Front. Biosci.* 28:127.
65. Masoumzadeh, E., J. Ying, …, A. Bax. 2024. Proline Peptide Bond Isomerization in Ubiquitin Under Folding and Denaturing Conditions by Pressure-Jump NMR. *J. Mol. Biol.* 436:168587.
66. Pettitt, A. J., …, D. F. Hansen. 2024. An Integrative Characterization of Proline *Cis* and *Trans* Conformers in a Disordered Peptide. *Biophys. J.* 123:3798–3811.
67. Chaudhury, S., …, R. N. De Guzman. 2015. Nuclear Magnetic Resonance Characterization of the Type III Secretion System Tip Chaperone Protein PcrG of Pseudomonas aeruginosa. *Biochemistry.* 54:6576–6585.
68. Stenström, O., …, F. Ferrage. 2022. How does it really move? Recent progress in the investigation of protein nanosecond dynamics by NMR and simulation. *Curr. Opin. Struct. Biol.* 77:102459.
69. Bobrowski, K., K. L. Wierzchowski, …, M. Ciurak. 1990. Intramolecular Electron Transfer in Peptides Containing Methionine, Tryptophan and Tyrosine: A Pulse Radiolysis Study. *Int. J. Radiat. Biol.* 57:919–932.
70. Wriggers, W., S. Chakravarty, and P. A. Jennings. 2005. Control of protein functional dynamics by peptide linkers. *Biopolymers.* 80:736–746.

71. Marsh, J. A., and J. D. Forman-Kay. 2010. Sequence Determinants of Compaction in Intrinsically Disordered Proteins. *Biophys. J.* 98:2383–2390.

72. Koren, G., S. Meir, …, R. Beck. 2023. others Intramolecular structural heterogeneity altered by long-range contacts in an intrinsically disordered protein. *Proc. Natl. Acad. Sci. USA.* 120:e2220180120.

73. Krieger, F., A. Möglich, and T. Kiefhaber. 2005. Effect of Proline and Glycine Residues on Dynamics and Barriers of Loop Formation in Polypeptide Chains. *J. Am. Chem. Soc.* 127:3346–3352.

74. Krieger, F., B. Fierz, …, T. Kiefhaber. 2003. Dynamics of Unfolded Polypeptide Chains as Model for the Earliest Steps in Protein Folding. *J. Mol. Biol.* 332:265–274.

75. Perticaroli, S., J. D. Nickels, …, A. P. Sokolov. 2014. Dynamics and Rigidity in an Intrinsically Disordered Protein, β-Casein. *J. Phys. Chem. B.* 118:7317–7326.

76. Catte, A., M. Girych, …, S. Vilov. 2016. Molecular electrometer and binding of cations to phospholipid bilayers. *Phys. Chem. Chem. Phys.* 18:32560–32569.

77. Ahmed, M. C., L. K. Skaanning, …, K. Lindorff-Larsen. 2021. Refinement of α-Synuclein Ensembles Against SAXS Data: Comparison of Force Fields and Methods. *Front. Mol. Biosci.* 8:654333.

78. Borthakur, K., T. R. Sisk, …, P. Robustelli. 2025. Determining accurate conformational ensembles of intrinsically disordered proteins at atomic resolution. *Nat. Commun.* 16:9036.

Biophysical Journal, Volume *125*

Supplemental information

Backbone rigidity of disordered protein linkers from NMR experiments and MD simulations

Efstathia Mantzari, Cajsa K. Malm, Ricky Nencini, Amanda E. Sandelin, and O.H. Samuli Ollila

Supplementary information: Backbone rigidity of disordered protein linkers from NMR experiments and MD simulations

Efstathia Mantzari,[†,‡,¶] Cajsa K. Malm,[†] Ricky Nencini,[‡,§] Amanda E. Sandelin,[‖,‡] and O. H. Samuli Ollila[*,†,‡]

†*VTT Technical Research Centre of Finland, 02044 Espoo, Finland*

‡*Institute of Biotechnology, University of Helsinki, 00014 Helsinki, Finland*

¶*Department of Chemistry and Materials Science, Aalto University, 00076 Espoo, Finland*

§*Division of Pharmaceutical Biosciences, Faculty of Pharmacy, University of Helsinki, 00014 Helsinki, Finland*

‖*Division of Pharmacology and Pharmacotherapy, Faculty of Pharmacy, University of Helsinki, 00014 Helsinki, Finland*

E-mail: samuli.ollila@vtt.fi

Supplementary results

NMR experiments

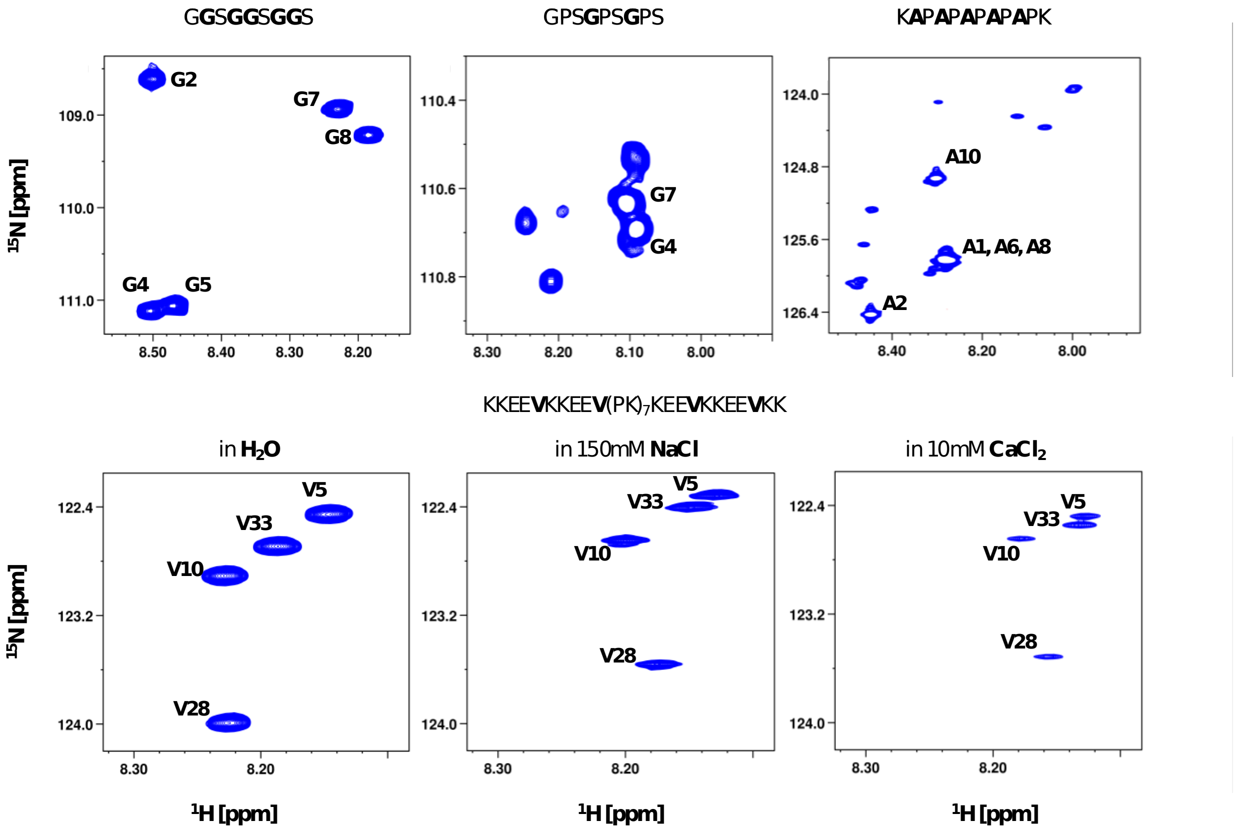

Figure S1: ^{1}H-^{15}N HSQC spectra for the short peptides (first row) and the charged peptide under different conditions (second row). Amino acid ^{1}5N labelled residues are highlighted in bold. Created with TopSpin 4.3.0.

Results from QEBSS simulations

$(GGS)_3$

Force field	Replica	R1 RMSD	R1 (%)	R2 RMSD	R2 (%)	hetNOE RMSD	hetNOE (%)	Sum (%)
AMBER99SB-DISP	replica04	0.053	100	0.24	110	0.48	110	320
AMBER99SB-DISP	replica01	0.063	120	0.24	110	0.43	100	330
AMBER99SB-DISP	replica03	0.071	130	0.25	110	0.45	100	350
AMBER99SB-DISP	replica05	0.068	130	0.28	130	0.47	110	360
AMBER99SB-DISP	replica02	0.071	130	0.27	120	0.48	110	370
DESAMBER	replica02	0.092	170	0.22	100	0.55	130	400
DESAMBER	replica03	0.088	160	0.27	120	0.53	120	410
DESAMBER	replica04	0.096	180	0.25	110	0.53	120	420
DESAMBER	replica05	0.092	170	0.28	130	0.53	120	420
DESAMBER	replica01	0.092	170	0.28	130	0.54	130	430
AMBER99SBWS	replica04	0.096	180	0.3	140	0.52	120	440
AMBER99SBWS	replica01	0.1	190	0.31	140	0.52	120	450
AMBER99SBWS	replica03	0.098	180	0.3	140	0.54	130	450
AMBER99SBWS	replica05	0.097	180	0.3	140	0.57	130	450
AMBER99SBWS	replica02	0.1	190	0.29	130	0.59	140	460
AMBER03WS	replica02	0.12	230	0.27	120	0.54	130	480
AMBER03WS	replica01	0.12	230	0.32	140	0.53	120	490
CHARMM36M	replica01	0.13	240	0.29	130	0.57	130	510
AMBER03WS	replica05	0.13	240	0.33	150	0.59	140	520
CHARMM36M	replica05	0.13	250	0.32	150	0.57	130	520
AMBER03WS	replica03	0.13	250	0.33	150	0.55	130	530
AMBER03WS	replica04	0.13	250	0.3	140	0.62	150	530
CHARMM36M	replica02	0.14	250	0.34	150	0.6	140	540
CHARMM36M	replica04	0.14	260	0.33	150	0.63	150	560
CHARMM36M	replica03	0.14	270	0.32	150	0.65	150	570

Figure S2: $(GGS)_3$: RMSD and percentage difference to experimental values for relaxation rates R1, R2 and hetNOE. Ranking numbers are determined by their fraction of the lowest RMSD value for each spin relaxation rate. Simulations deviating less than 50% from the lowest RMSD for all spin relaxation rates are selected as best and highlighted in bold

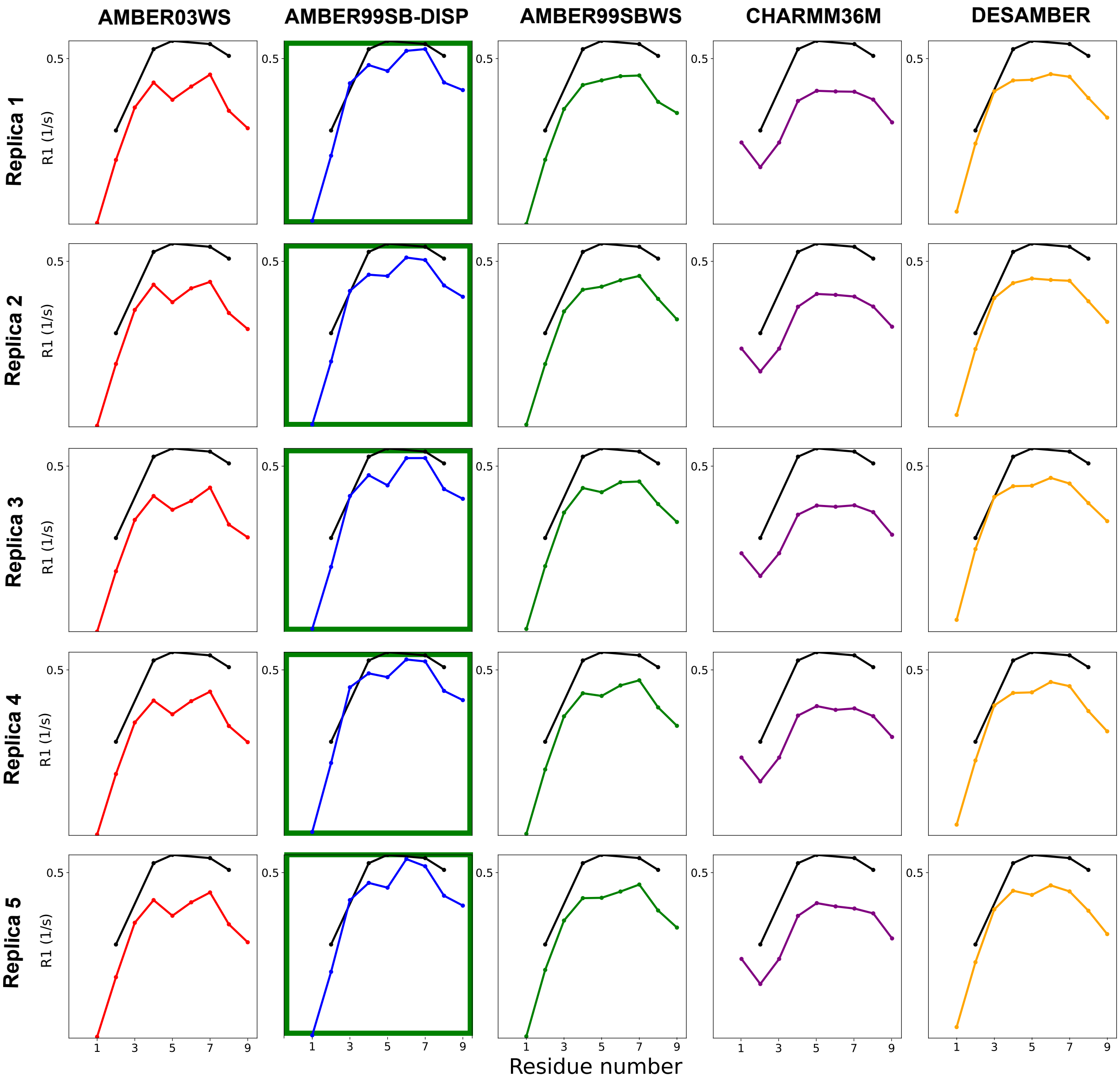

Figure S3: R1 relaxation rates from 25 simulations of the five replicas and force fields for the $(GGS)_3$ peptide (colored lines), plotted against experimental R1 rates (black lines).

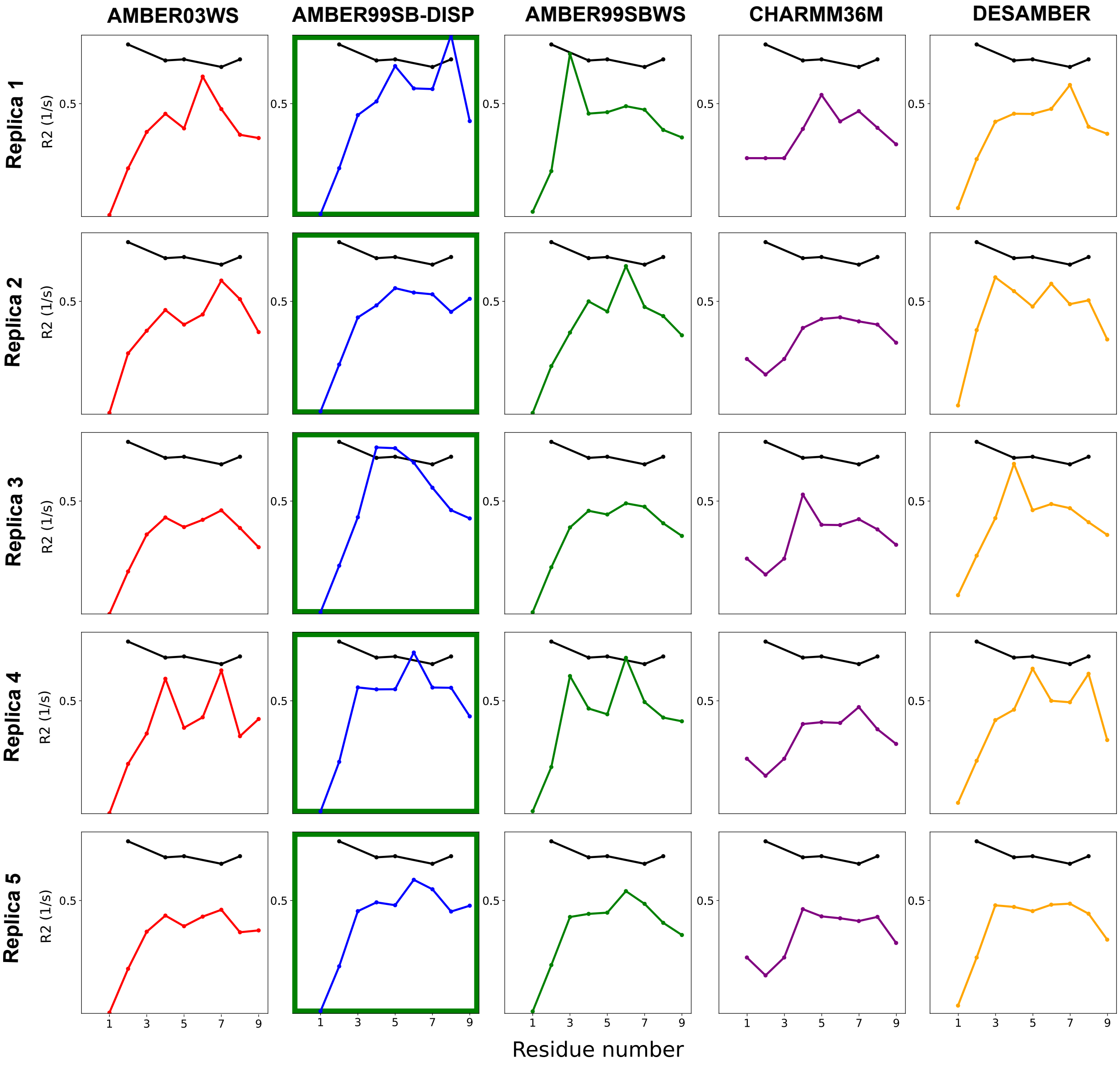

Figure S4: R2 relaxation rates from 25 simulations of the five replicas and force fields for the $(GGS)_3$ peptide (colored lines), plotted against experimental R2 rates (black lines).

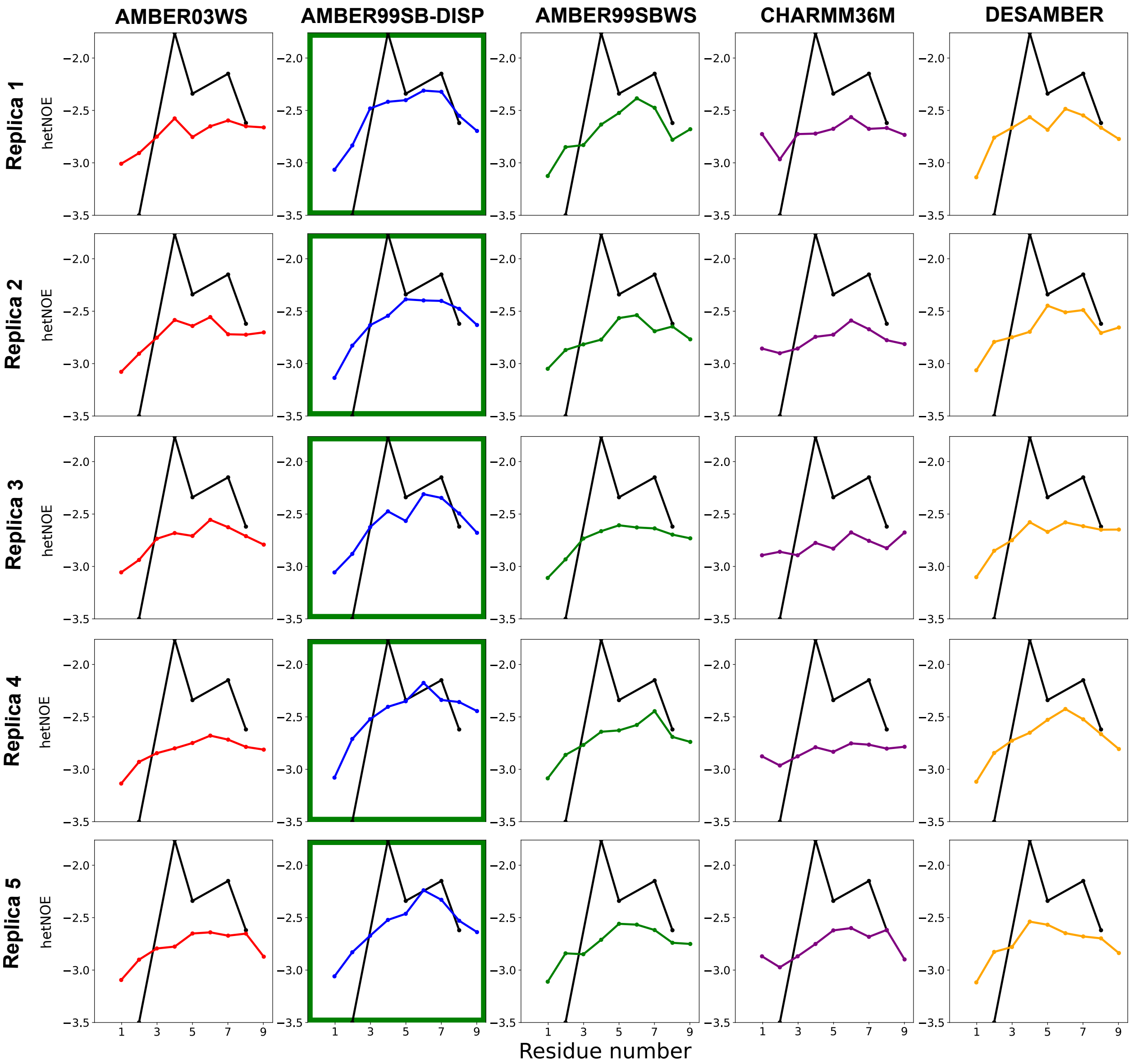

Figure S5: HetNoe relaxation rates from 25 simulations of the five replicas and force fields for the $(GGS)_3$ peptide (colored lines), plotted against experimental hetNoe rates (black lines).

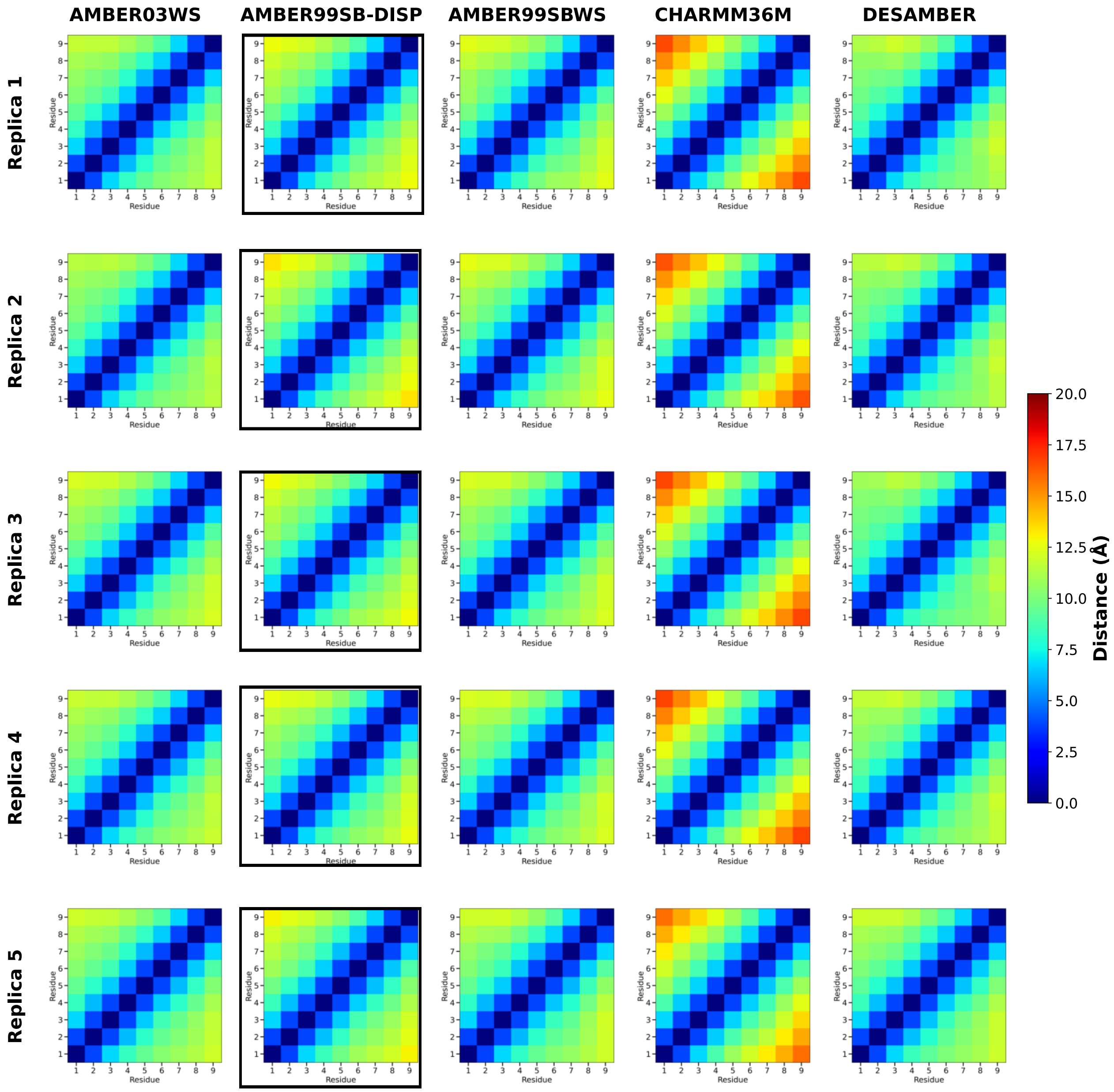

Figure S6: Distance maps for the $(GGS)_3$ peptide.

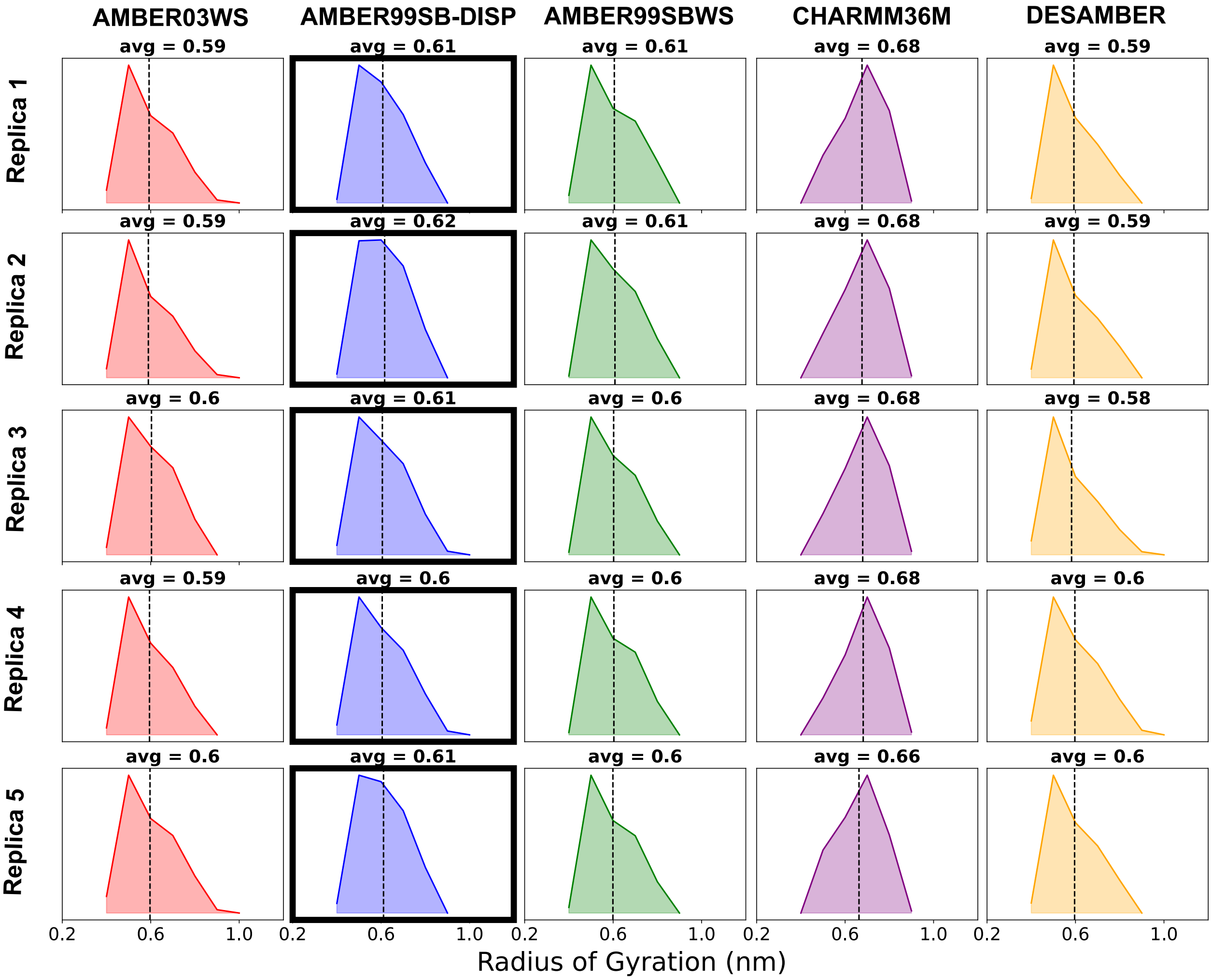

Figure S7: Radius of gyration distribution generated from five different initial structures and force fields for the $(GGS)_3$ peptide.

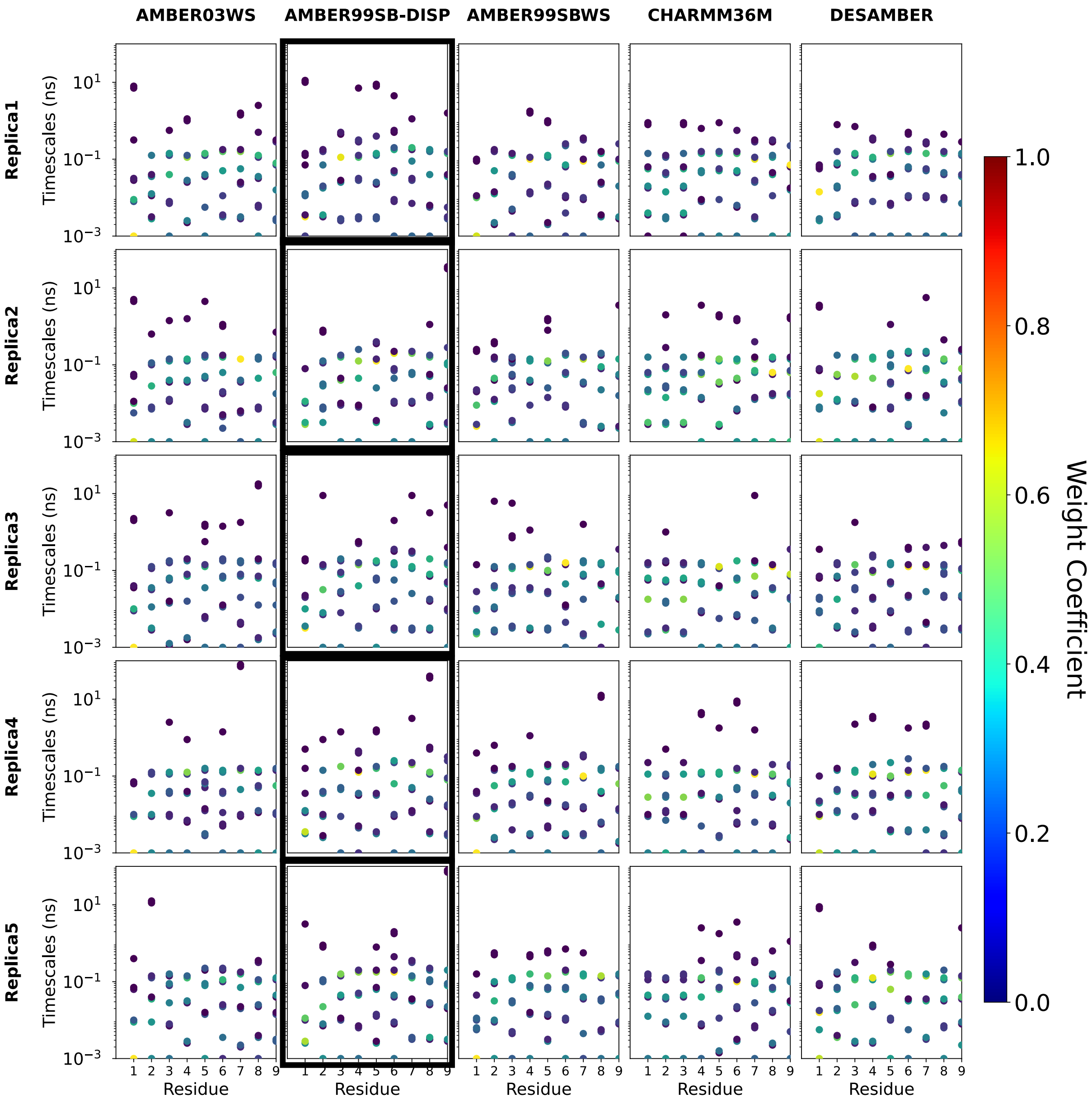

Figure S8: Timescales of backbone N-H bonds in $(GGS)_3$.

$(GPS)_3$

Force field	Replica	R1 RMSD	R1 (%)	R2 RMSD	R2 (%)	hetNOE RMSD	hetNOE (%)	Sum (%)
DESAMBER	replica03	0.012	100	0.047	180	0.21	190	470
AMBER99SB-DISP	replica04	0.037	320	0.027	100	0.21	190	610
DESAMBER	replica01	0.02	170	0.056	210	0.28	260	640
AMBER99SB-DISP	replica02	0.043	370	0.054	200	0.14	130	700
AMBER99SB-DISP	replica03	0.026	220	0.11	430	0.13	120	770
DESAMBER	replica05	0.027	230	0.076	280	0.35	320	830
DESAMBER	replica02	0.023	200	0.096	360	0.34	310	870
AMBER99SB-DISP	replica01	0.043	370	0.12	450	0.11	100	920
DESAMBER	replica04	0.022	190	0.089	330	0.45	410	940
AMBER99SBWS	replica02	0.031	260	0.085	320	0.6	550	1100
AMBER99SBWS	replica05	0.046	400	0.07	260	0.46	420	1100
AMBER99SB-DISP	replica05	0.075	650	0.11	410	0.11	100	1200
AMBER99SBWS	replica01	0.043	370	0.12	440	0.5	460	1300
AMBER03WS	replica02	0.068	580	0.11	430	0.44	400	1400
AMBER99SBWS	replica04	0.058	500	0.11	410	0.56	510	1400
AMBER03WS	replica05	0.064	550	0.12	460	0.44	410	1400
AMBER03WS	replica01	0.062	540	0.14	510	0.44	410	1500
AMBER99SBWS	replica03	0.059	510	0.14	520	0.49	450	1500
AMBER03WS	replica04	0.068	580	0.14	520	0.58	530	1600
AMBER03WS	replica03	0.082	710	0.12	460	0.59	550	1700
CHARMM36M	replica02	0.12	1000	0.11	410	0.87	800	2200
CHARMM36M	replica03	0.1	870	0.18	660	0.78	720	2200
CHARMM36M	replica01	0.11	940	0.2	750	0.9	820	2500
CHARMM36M	replica04	0.12	1100	0.15	550	0.9	830	2500
CHARMM36M	replica05	0.11	970	0.2	750	0.92	840	2600

Figure S9: $(GPS)_3$: RMSD and percentage difference to experiment values for relaxation rates R1, R2 and hetNOE. Ranking numbers are determined by their fraction of the lowest RMSD value for each spin relaxation rate. Due to insufficient number of experimental data points, comparison numbers are very large, surpassing the 150. Thus, in this case the simulation exhibiting comparison numbers close to 150 for R1 and R2 rates and one of the smallest comparison numbers for hetNOE, at the same time, was selected.

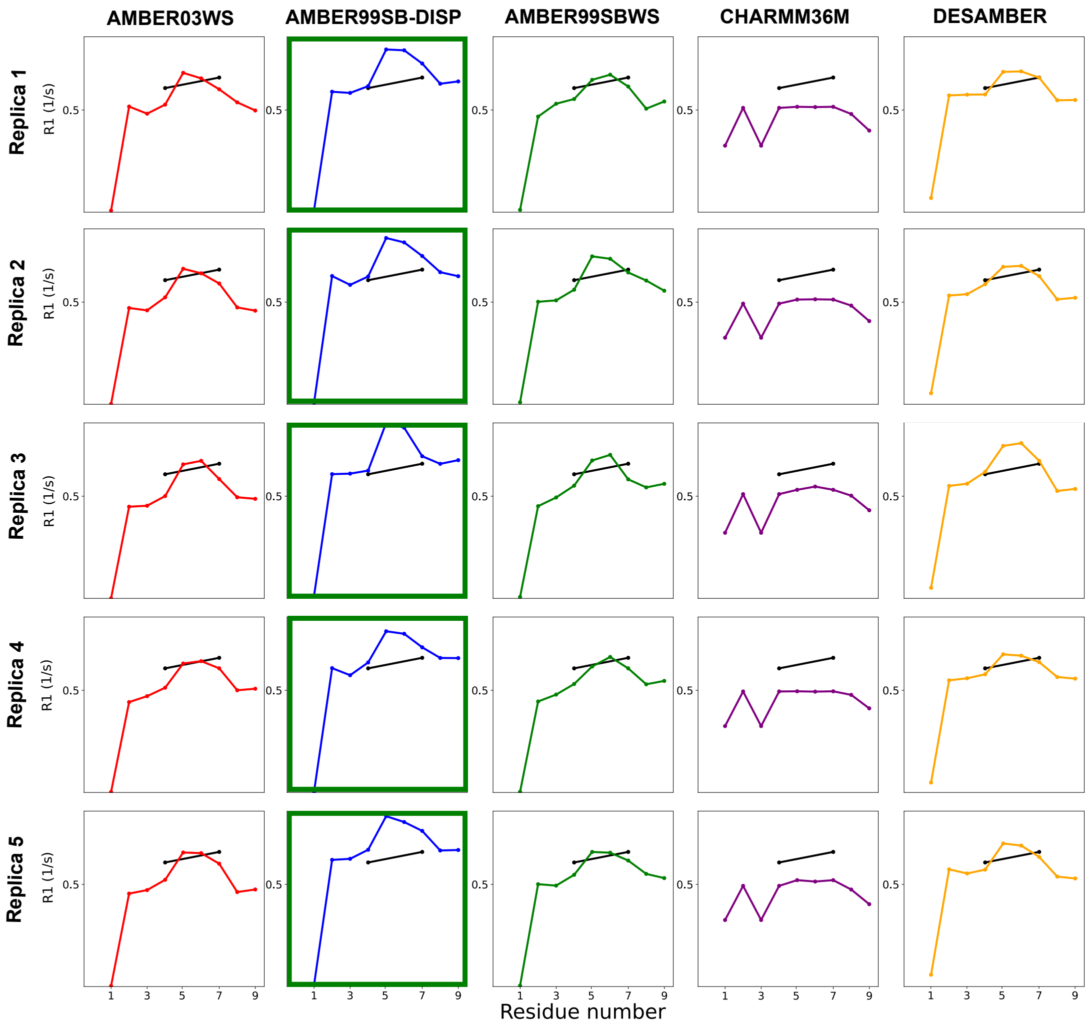

Figure S10: R1 relaxation rate plots for 25 simulations of different models and force fields of $(GPS)_3$ peptide.

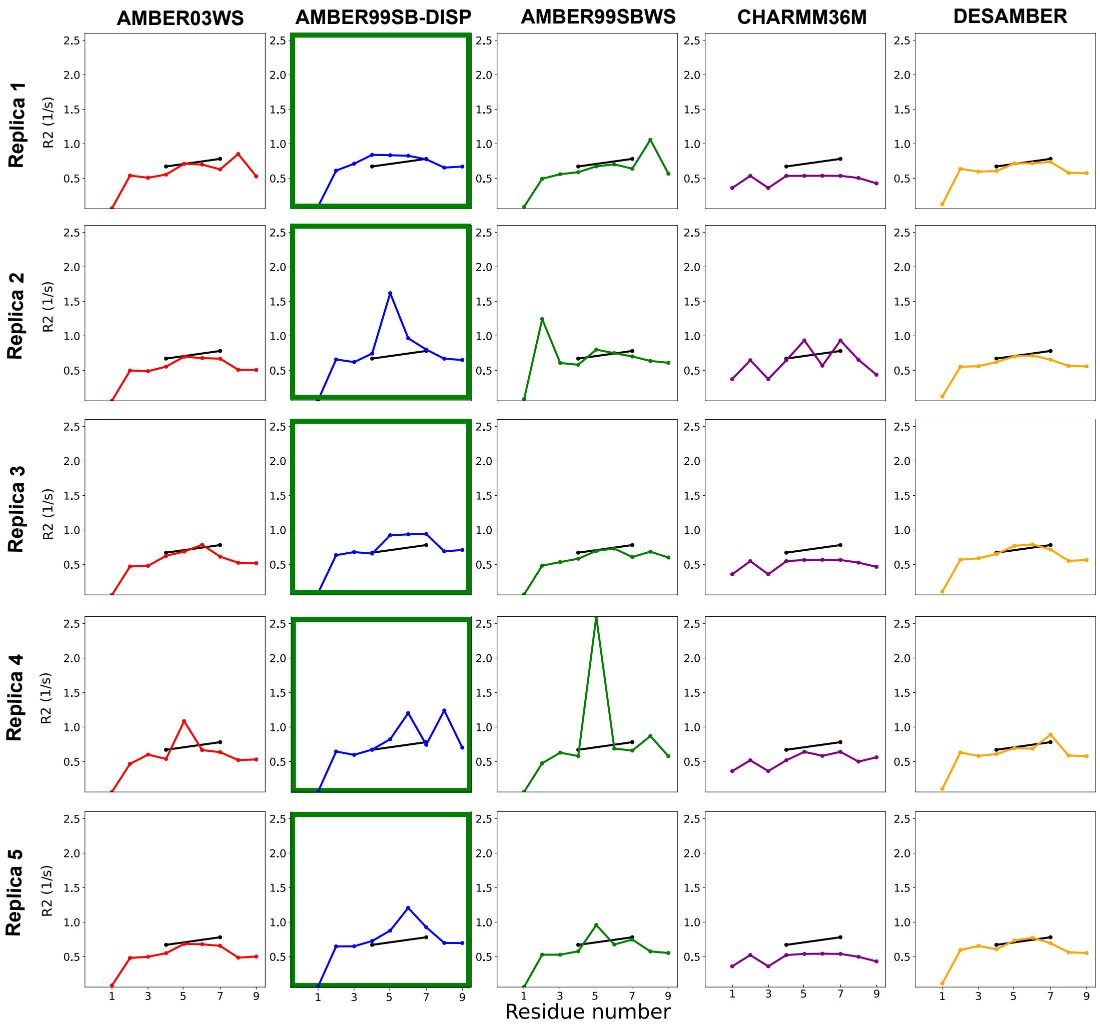

Figure S11: R2 relaxation rate plots for 25 simulations of different models and force fields of $(GPS)_3$ peptide.

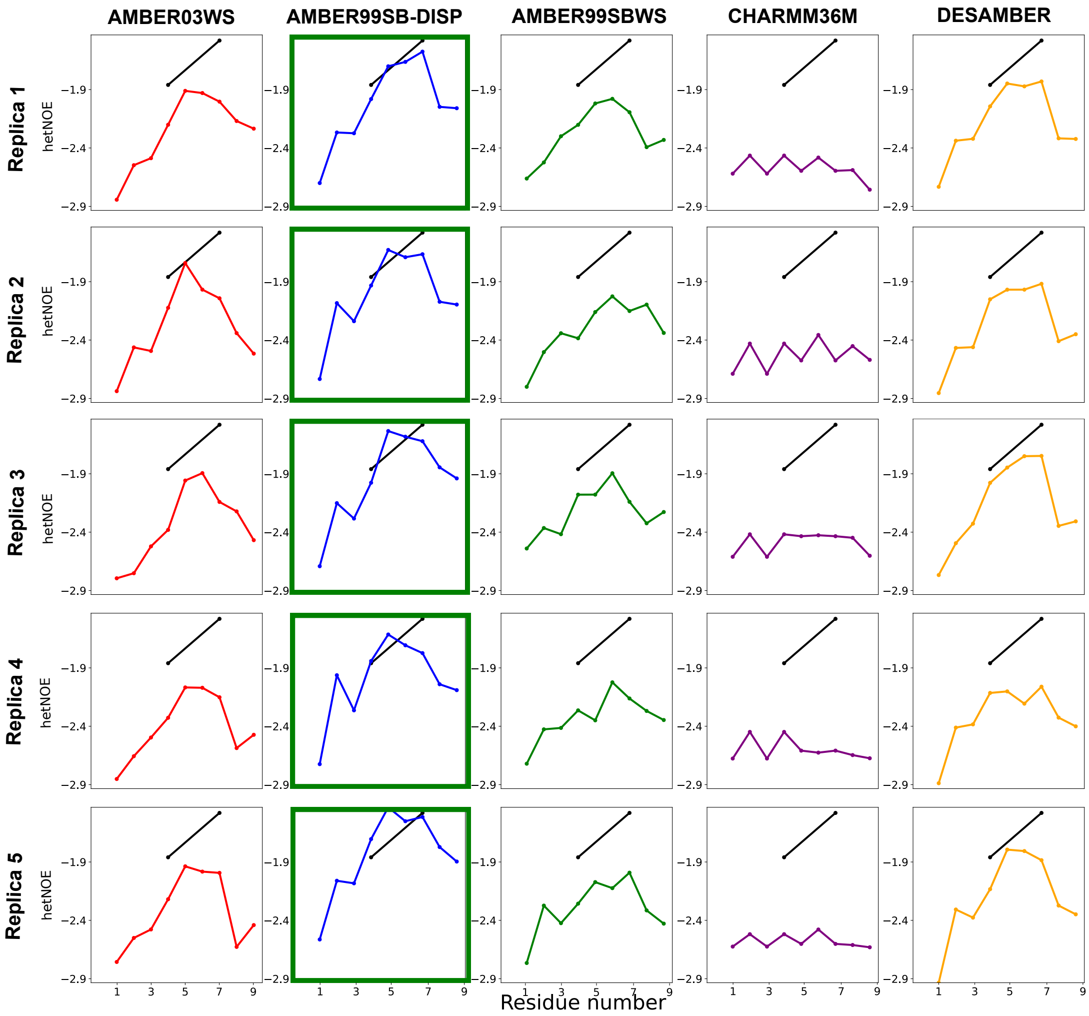

Figure S12: HetNoe relaxation time plots for 25 simulations of different models and force fields of $(GPS)_3$ peptide.

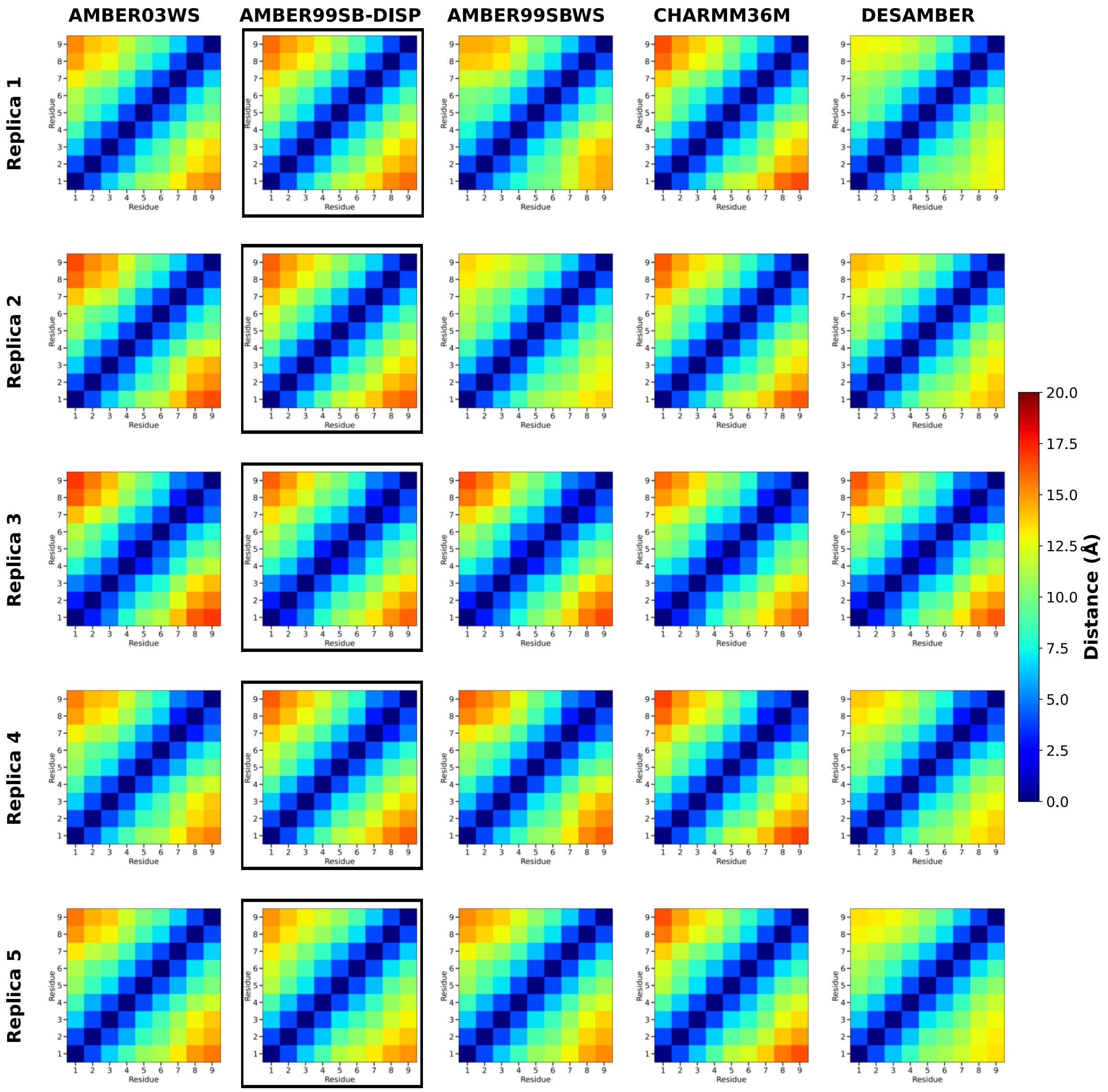

Figure S13: Distance maps for the $(GPS)_3$ peptide.

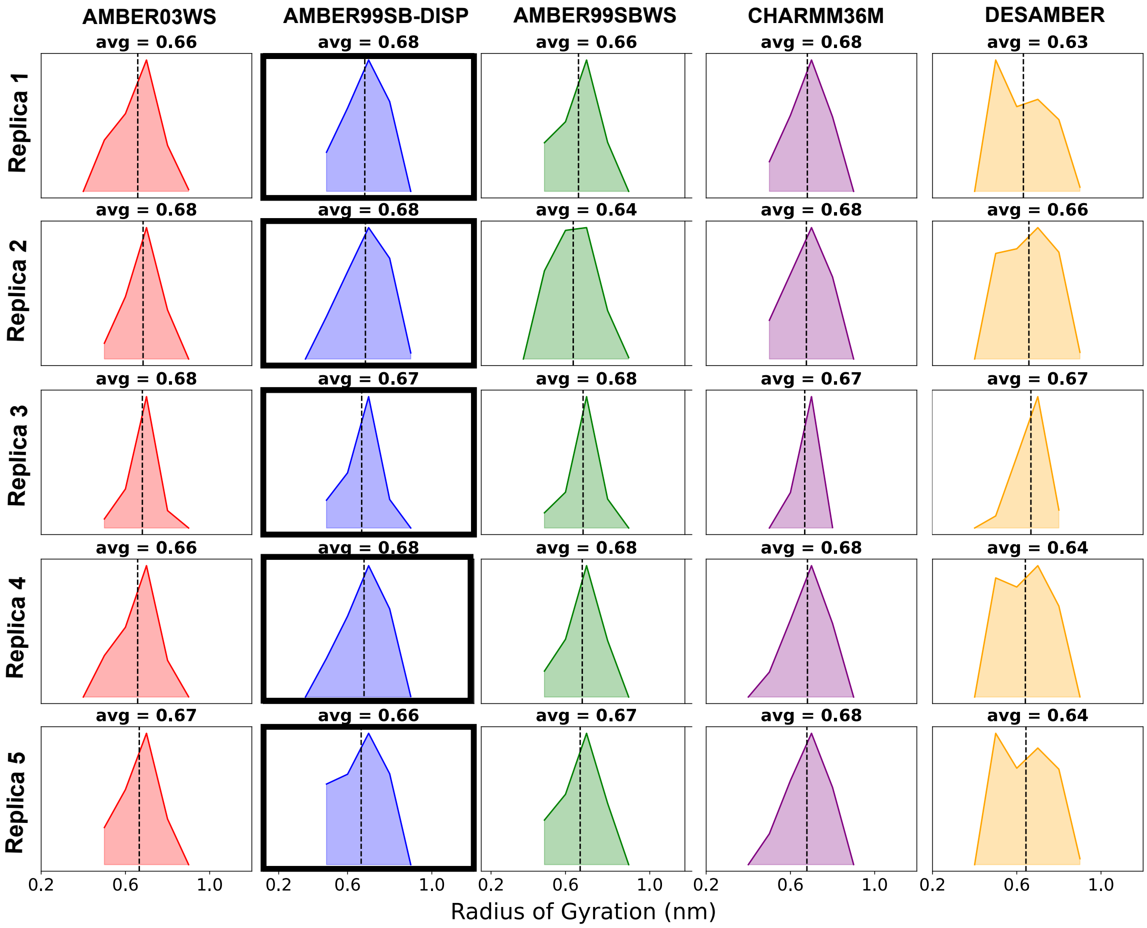

Figure S14: Radius of gyration distribution generated from five different initial structures and force fields for the $(GPS)_3$ peptide.

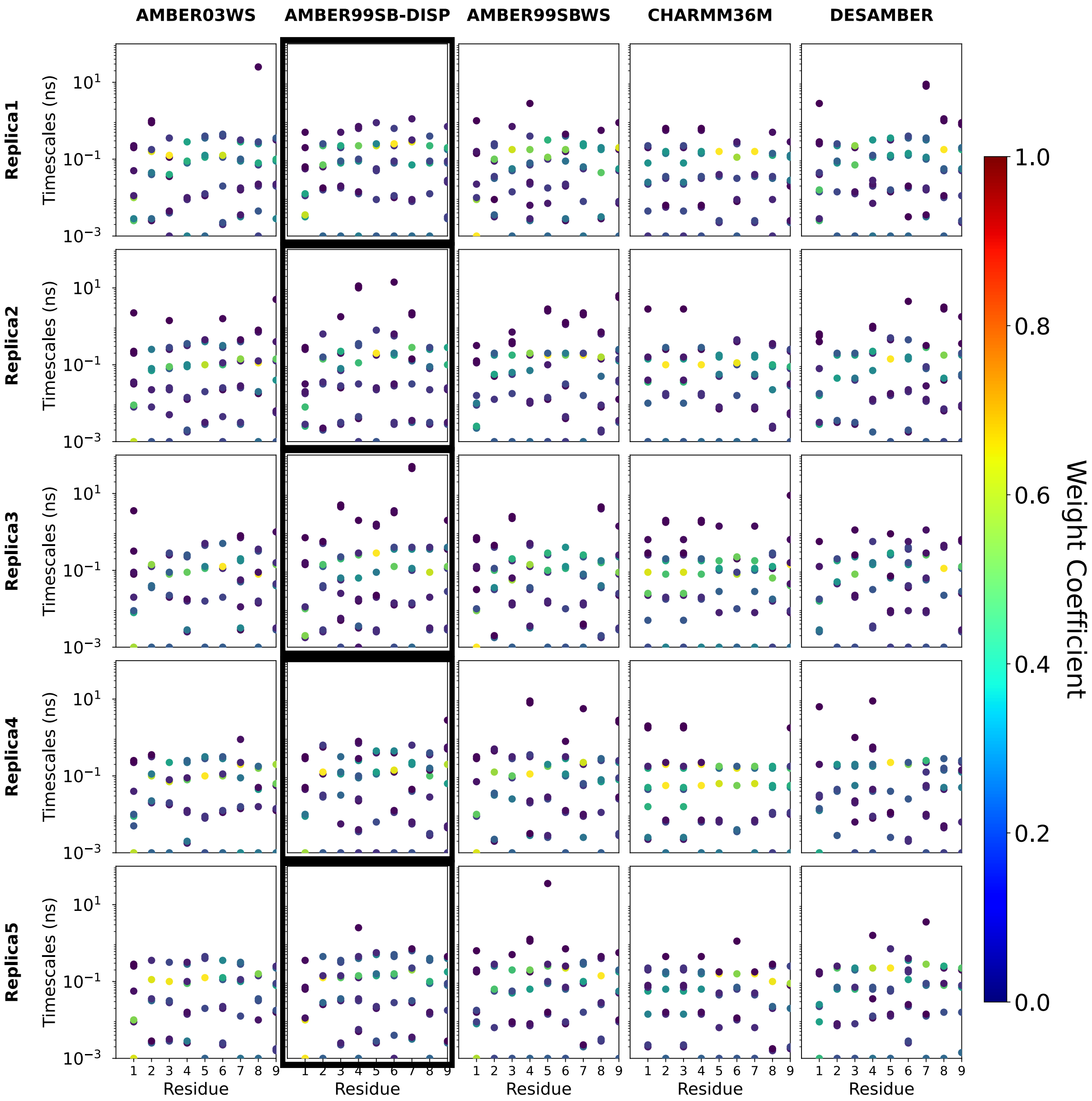

Figure S15: Timescales of backbone N-H bonds in $(GPS)_3$.

$K(AP)_5K$

Force field	Replica	R1 RMSD	R1 (%)	R2 RMSD	R2 (%)	hetNOE RMSD	hetNOE (%)	Sum (%)
DESAMBER	replica02	0.076	120	0.046	100	0.41	120	340
DESAMBER	replica04	0.1	160	0.08	170	0.49	140	470
DESAMBER	replica01	0.098	150	0.099	210	0.4	120	480
AMBER03WS	replica05	0.14	230	0.065	140	0.8	230	600
AMBER99SB-DISP	replica05	0.1	160	0.2	440	0.39	110	700
AMBER99SBWS	replica01	0.12	190	0.16	360	0.64	180	730
AMBER99SB-DISP	replica03	0.1	160	0.21	450	0.45	130	740
AMBER03WS	replica01	0.12	180	0.17	380	0.71	210	760
AMBER99SB-DISP	replica04	0.13	210	0.2	440	0.48	140	790
AMBER03WS	replica02	0.14	220	0.16	350	0.84	240	820
AMBER99SBWS	replica04	0.17	260	0.15	330	0.83	240	830
AMBER99SBWS	replica05	0.16	250	0.16	340	0.81	230	830
AMBER03WS	replica03	0.16	250	0.19	410	0.74	210	880
AMBER99SBWS	replica02	0.16	250	0.19	400	0.79	230	890
AMBER99SBWS	replica03	0.16	250	0.19	420	0.87	250	920
DESAMBER	replica05	0.17	260	0.37	790	0.43	120	1200
AMBER03WS	replica04	0.18	280	0.34	730	0.92	270	1300
DESAMBER	replica03	0.064	100	0.64	1400	0.48	140	1600
CHARMM36M	replica03	0.38	600	0.37	800	1.1	320	1700
CHARMM36M	replica05	0.34	540	0.46	1000	1.1	320	1900
CHARMM36M	replica02	0.36	560	0.5	1100	1.1	310	2000
CHARMM36M	replica04	0.37	570	0.53	1100	1.1	320	2000
CHARMM36M	replica01	0.37	580	0.51	1100	1.4	400	2100
AMBER99SB-DISP	replica01	0.17	260	0.94	2000	0.35	100	2400
AMBER99SB-DISP	replica02	0.12	190	0.99	2200	0.46	130	2500

Figure S16: $K(AP)_5K$: RMSD and percentage difference to experiment values for relaxation rates R1, R2 and hetNOE. Ranking numbers are determined by their fraction of the lowest RMSD value for each spin relaxation rate. Simulations deviating less than 50 from the lowest RMSD for all spin relaxation rates are selected as best and highlighted in bold.

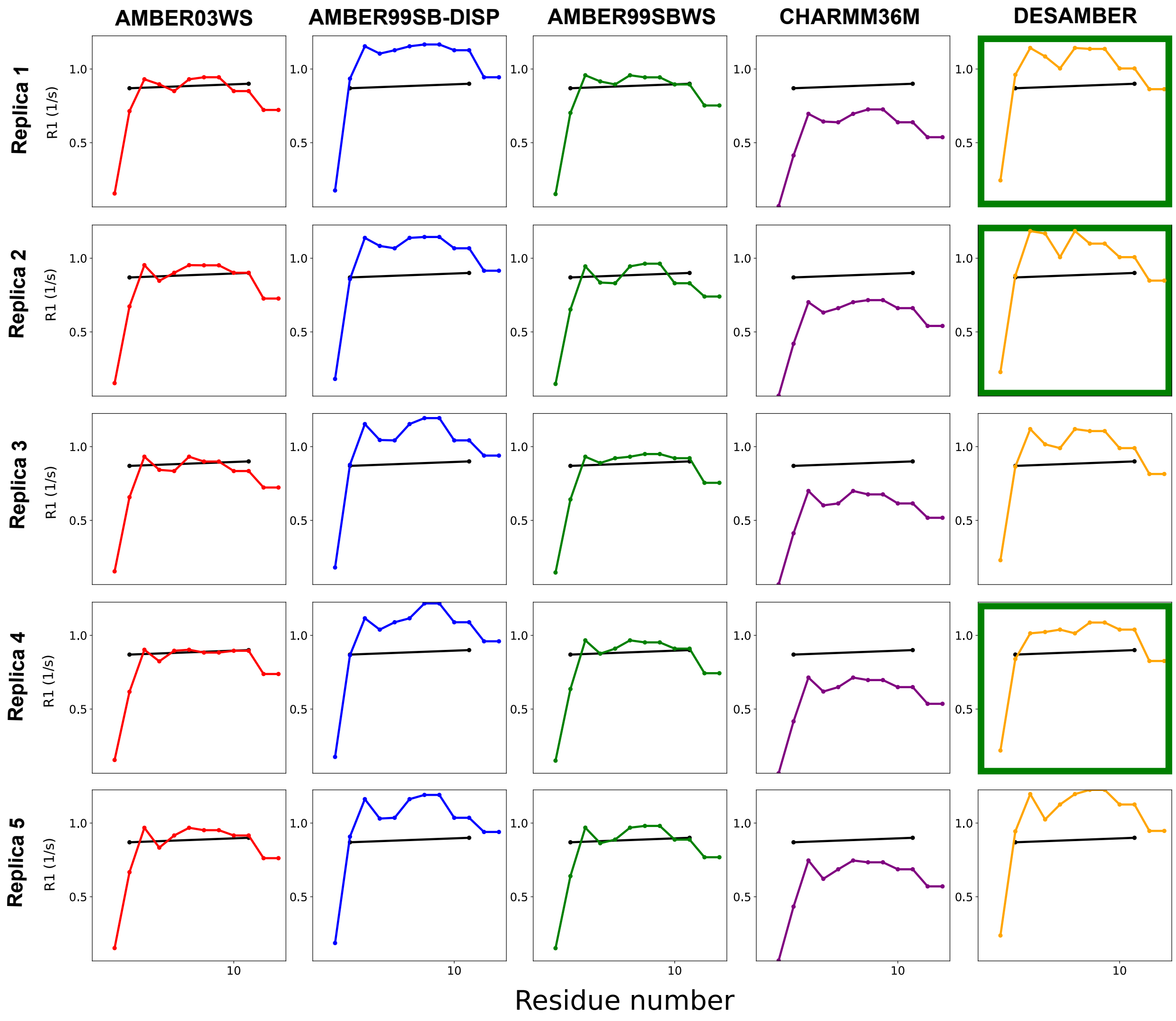

Figure S17: R1 relaxation rate plots for 25 simulations of different models and force fields of K(AP)$_5$K peptide.

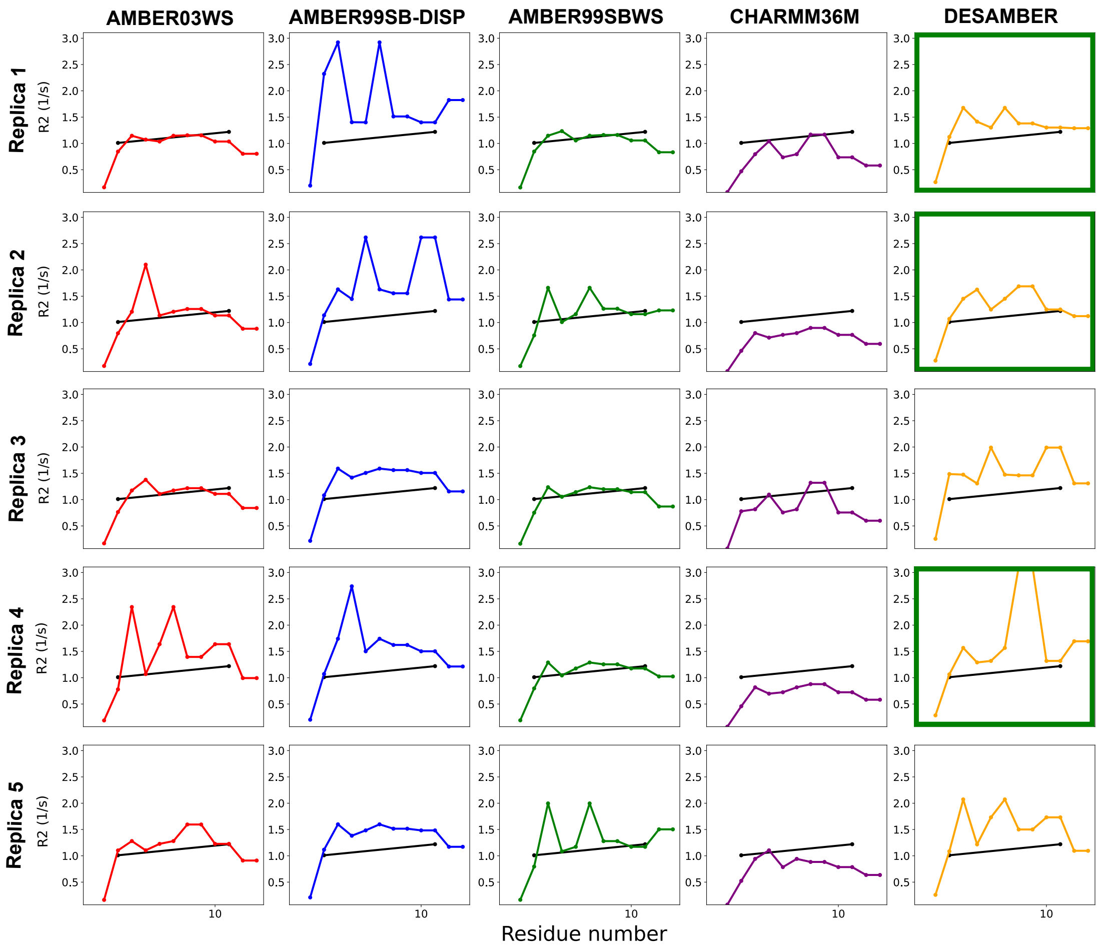

Figure S18: R2 relaxation rate plots for 25 simulations of different models and force fields of K(AP)$_5$K peptide.

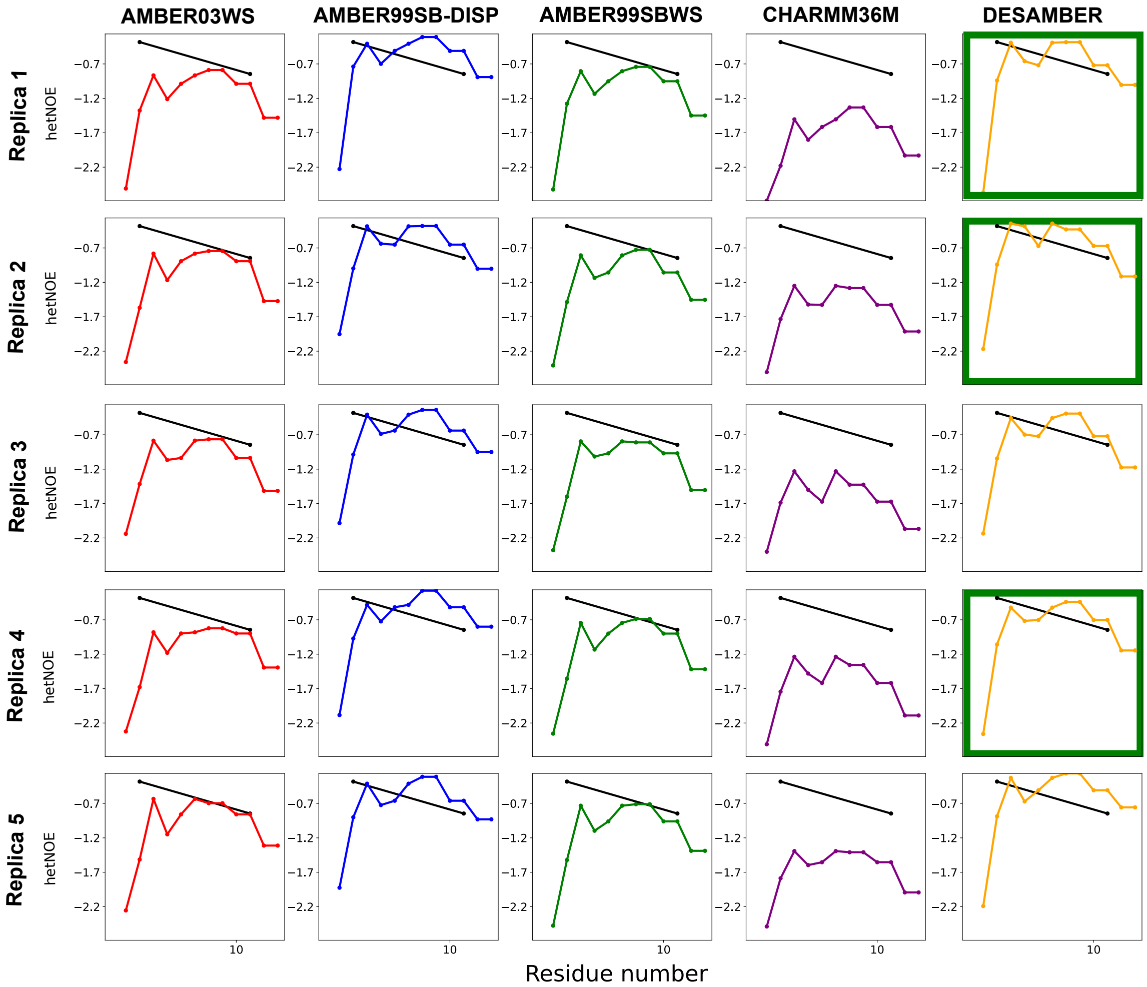

Figure S19: HetNoe relaxation time plots for 25 simulations of different models and force fields of $K(AP)_5K$ peptide.

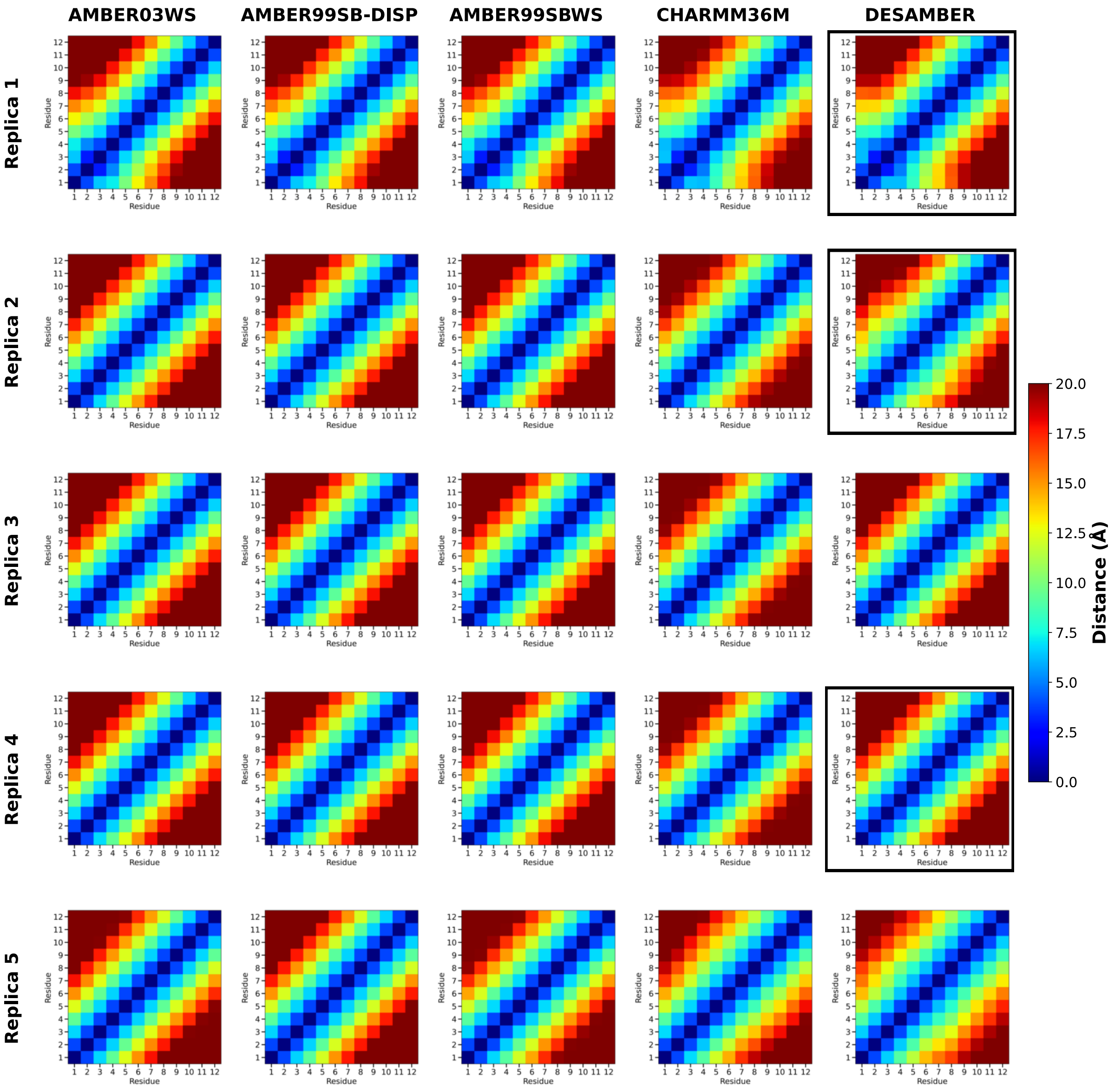

Figure S21: Distance maps for the $K(AP)_5K$ peptide.

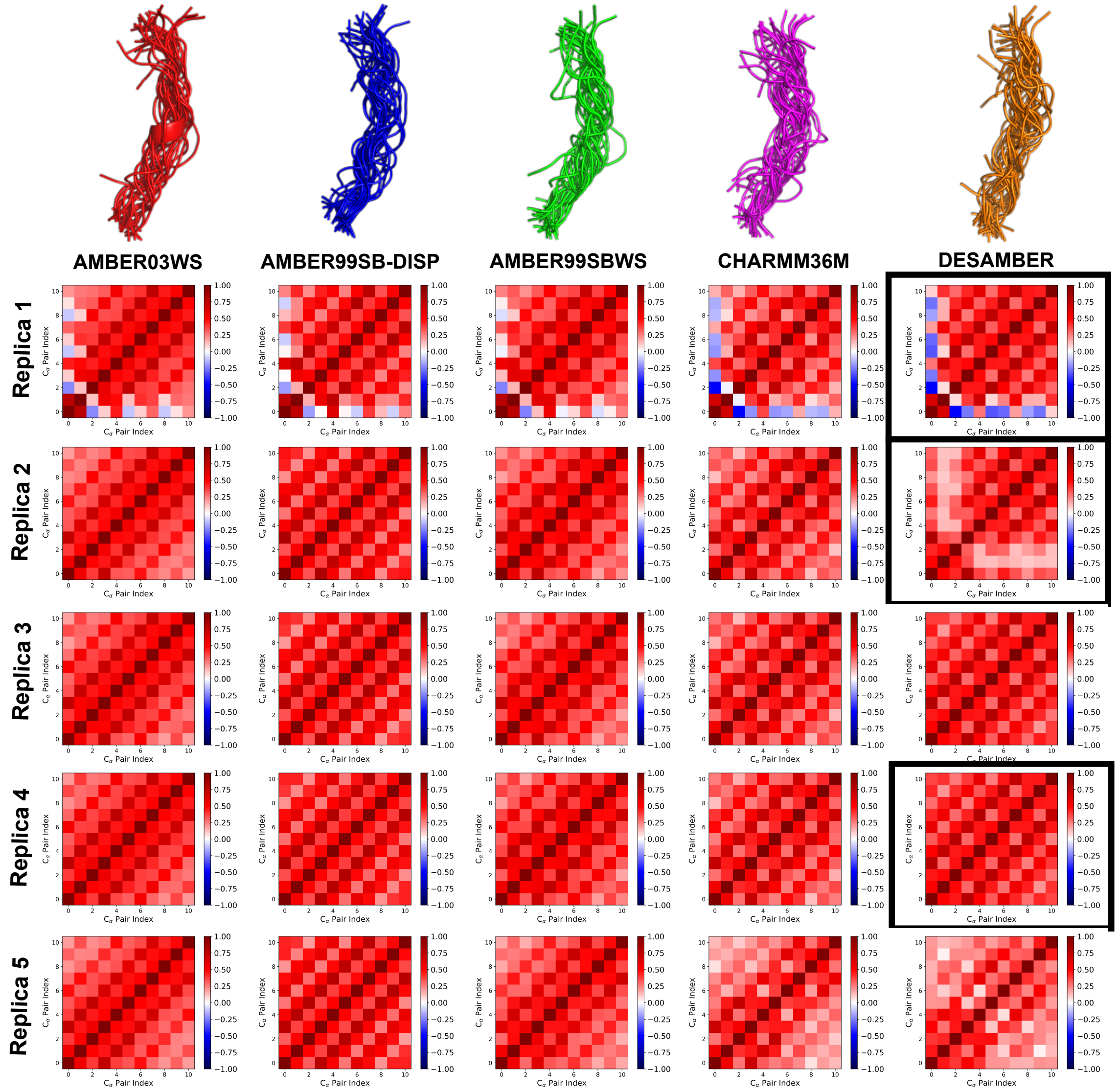

Figure S20: Consecutive Ca vector correlation maps generated from five different initial structures and force fields, along with representative snapshots for the K(AP)$_5$K peptide. Positive and negative values indicate positive and negative correlation respectively. The snapshots represent 5 overlayed frames from each force field for the same initial structure model of the peptide. The selected simulations from the quality evaluation are highlighted with the square.

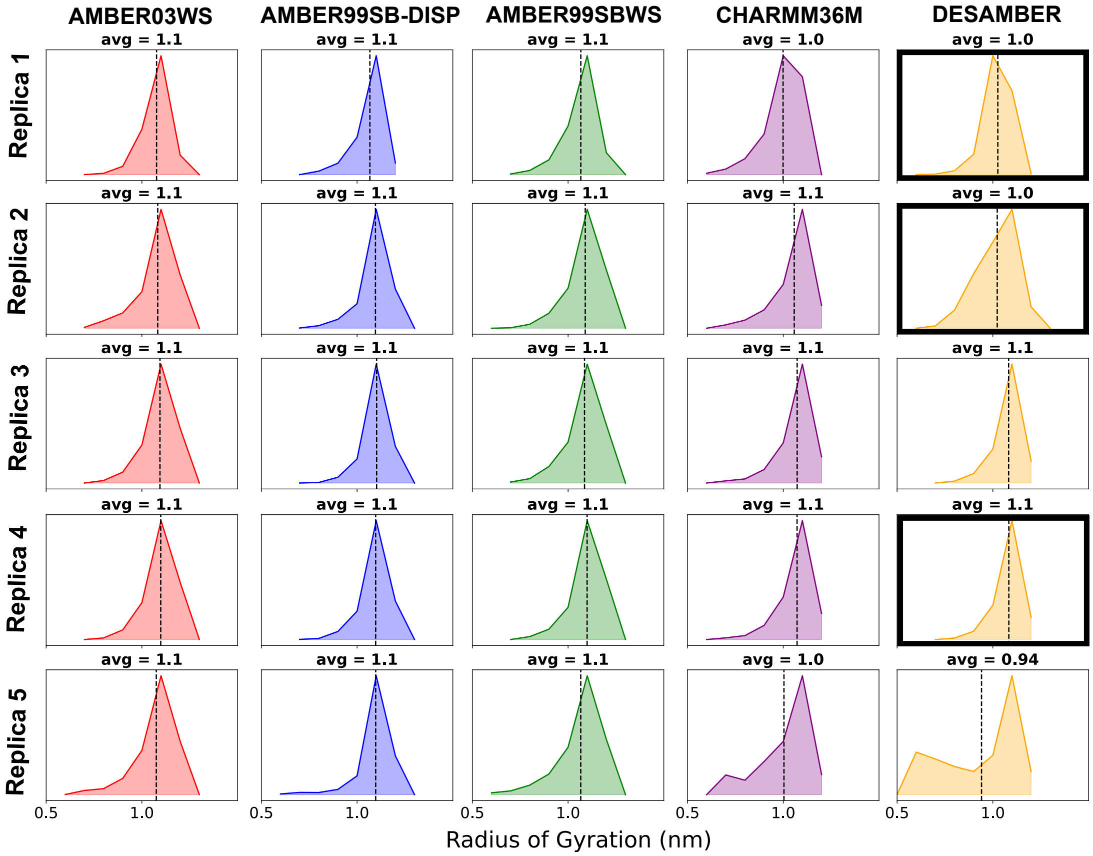

Figure S22: Radius of gyration distribution generated from five different initial structures and force fields for the $K(AP)_5K$ peptide.

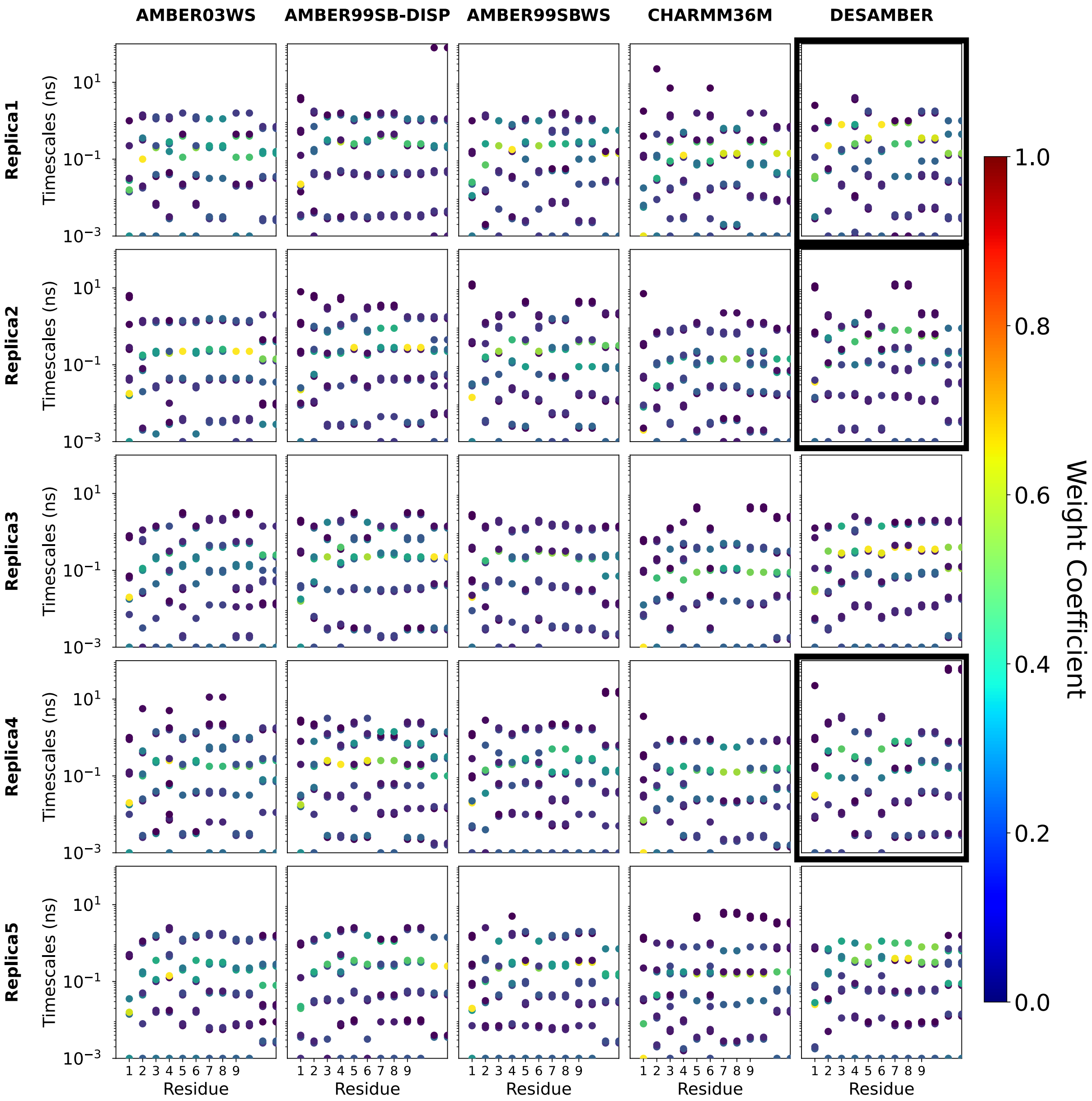

Figure S23: Timescales of of backbone N-H bonds in $K(AP)_5K$.

Charged sequence: KKEEVKKEEV$(PK)_7$KEEVKKEEVKK

Force field	Replica	R1 RMSD	R1 (%)	R2 RMSD	R2 (%)	hetNOE RMSD	hetNOE (%)	Sum (%)
CHARMM36M	replica01	0.064	140	0.37	170	0.23	120	430
AMBER03WS	replica04	0.08	170	0.28	130	0.25	130	430
AMBER03WS	replica01	0.079	170	0.31	140	0.25	130	440
AMBER03WS	replica02	0.094	200	0.22	100	0.26	140	440
CHARMM36M	replica04	0.091	200	0.42	190	0.37	190	580
DESAMBER	replica04	0.12	250	0.48	220	0.25	130	600
AMBER99SBWS	replica05	0.17	370	0.35	160	0.25	130	660
AMBER99SB-DISP	replica04	0.19	420	0.39	180	0.28	140	740
CHARMM36M	replica02	0.11	250	0.59	270	0.46	240	760
AMBER99SBWS	replica01	0.16	340	0.79	360	0.19	100	800
CHARMM36M	replica05	0.08	170	1.0	470	0.38	200	840
AMBER03WS	replica05	0.046	100	1.4	660	0.28	150	900
DESAMBER	replica02	0.15	320	1.0	480	0.26	130	940
AMBER99SBWS	replica03	0.26	550	1.0	460	0.21	110	1100
AMBER99SB-DISP	replica05	0.18	380	1.2	550	0.24	130	1100
AMBER99SBWS	replica04	0.26	570	1.0	460	0.24	130	1200
AMBER99SBWS	replica02	0.21	460	1.7	790	0.22	110	1400
CHARMM36M	replica03	0.074	160	2.7	1200	0.32	170	1500
DESAMBER	replica05	0.19	420	2.1	960	0.28	150	1500
AMBER03WS	replica03	0.097	210	2.7	1200	0.23	120	1600
DESAMBER	replica03	0.19	400	2.8	1300	0.27	140	1800
AMBER99SB-DISP	replica01	0.2	430	3.3	1500	0.25	130	2100
DESAMBER	replica01	0.15	330	3.6	1600	0.24	130	2100
AMBER99SB-DISP	replica02	0.17	360	3.8	1700	0.21	110	2200
AMBER99SB-DISP	replica03	0.21	460	3.8	1700	0.28	150	2300

Figure S24: charged peptide: RMSD and percentage difference to experiment values for relaxation rates R1, R2 and hetNOE. Ranking numbers are determined by their fraction of the lowest RMSD value for each spin relaxation rate. Simulations deviating less than 50 from the lowest RMSD for all spin relaxation rates are selected as best and highlighted in bold

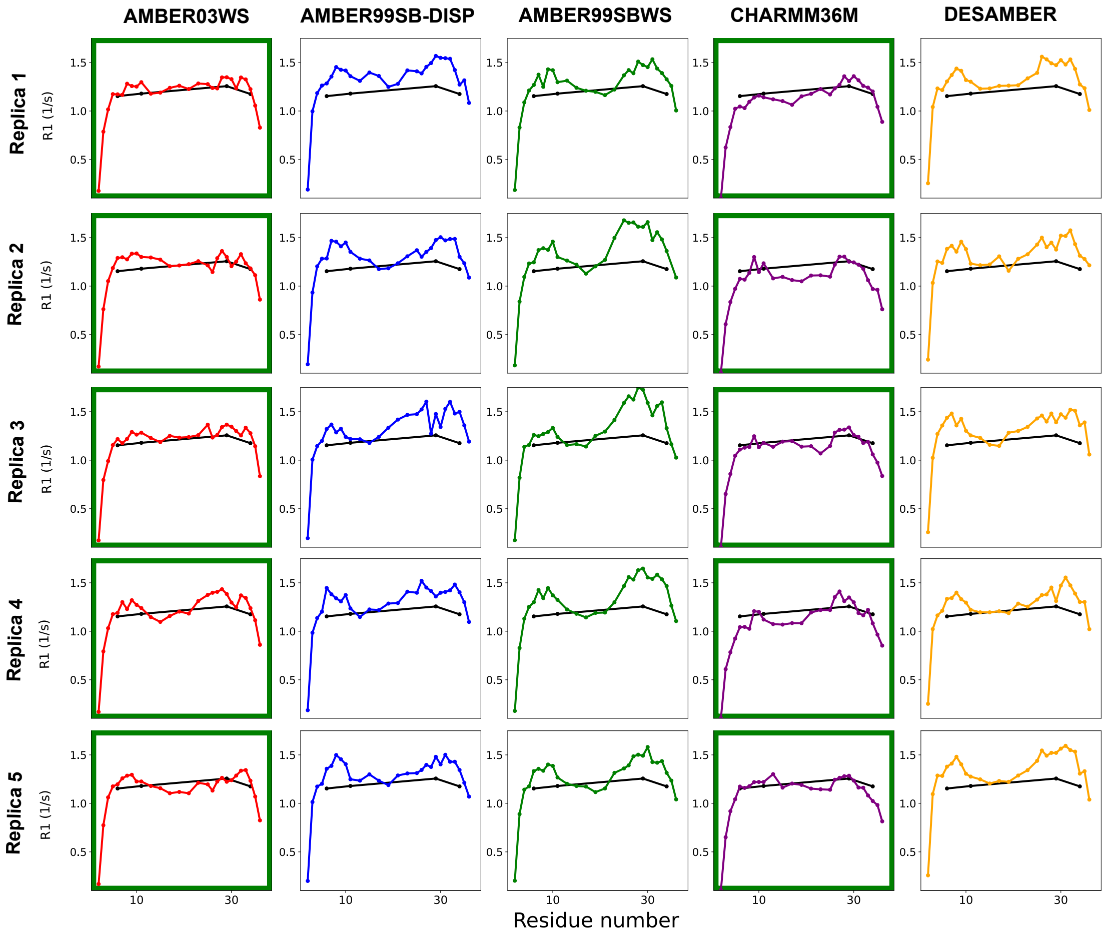

Figure S25: R1 relaxation rate plots for 25 simulations of different models and force fields of charged peptide.

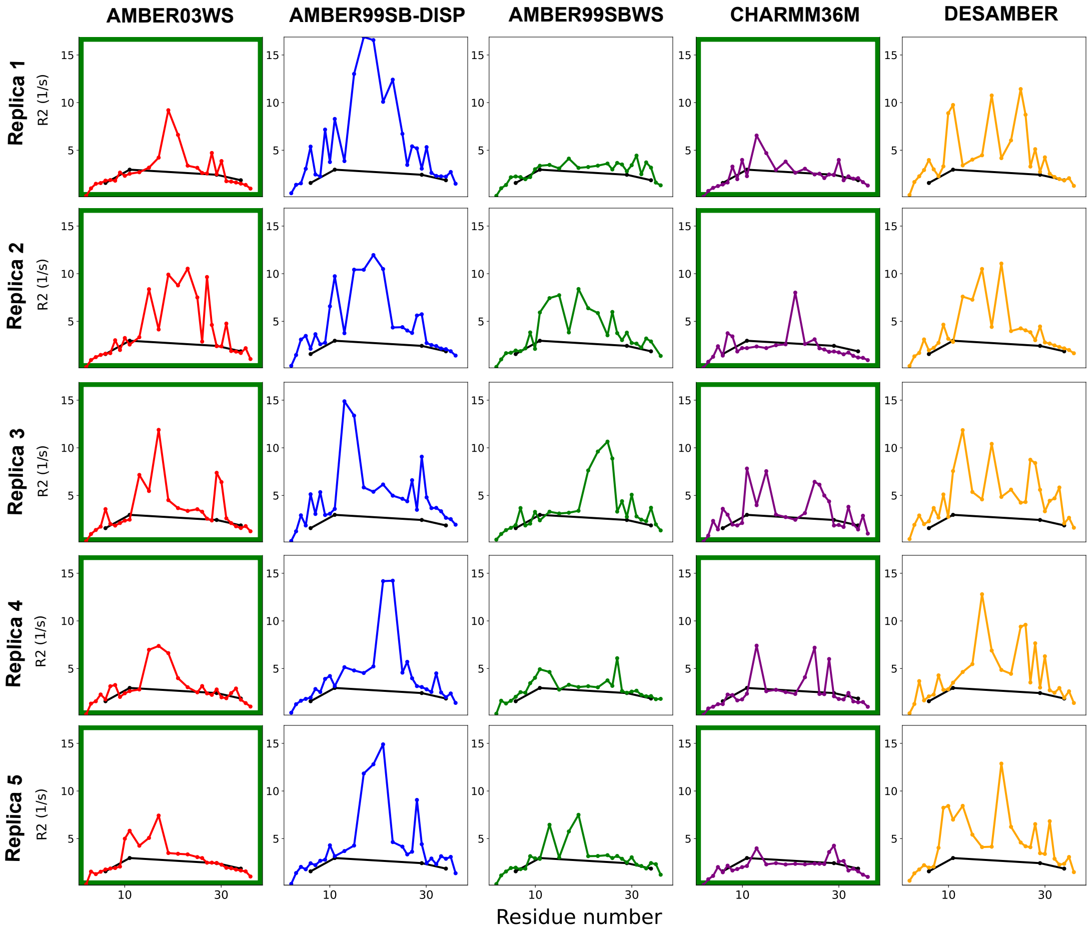

Figure S26: R2 relaxation rate plots for 25 simulations of different models and force fields of charged peptide.

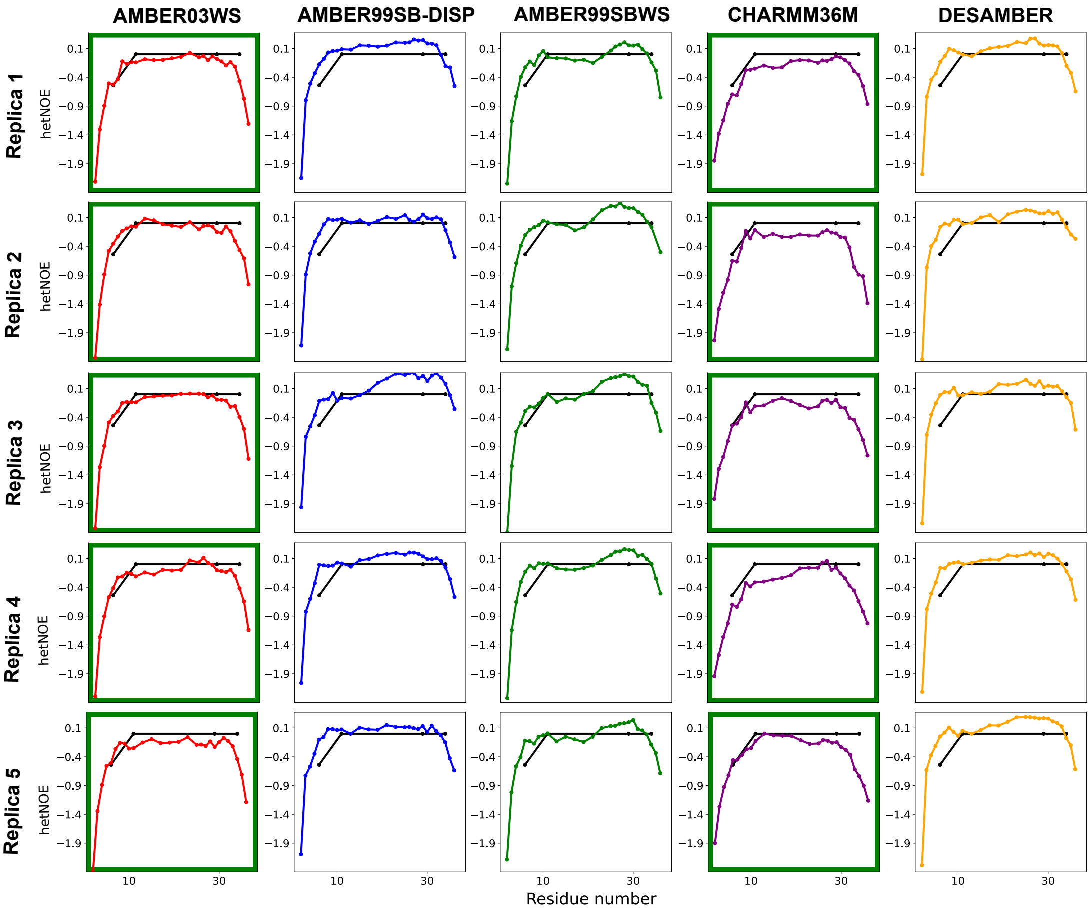

Figure S27: HetNoe relaxation time plots for 25 simulations of different models and force fields of charged peptide.

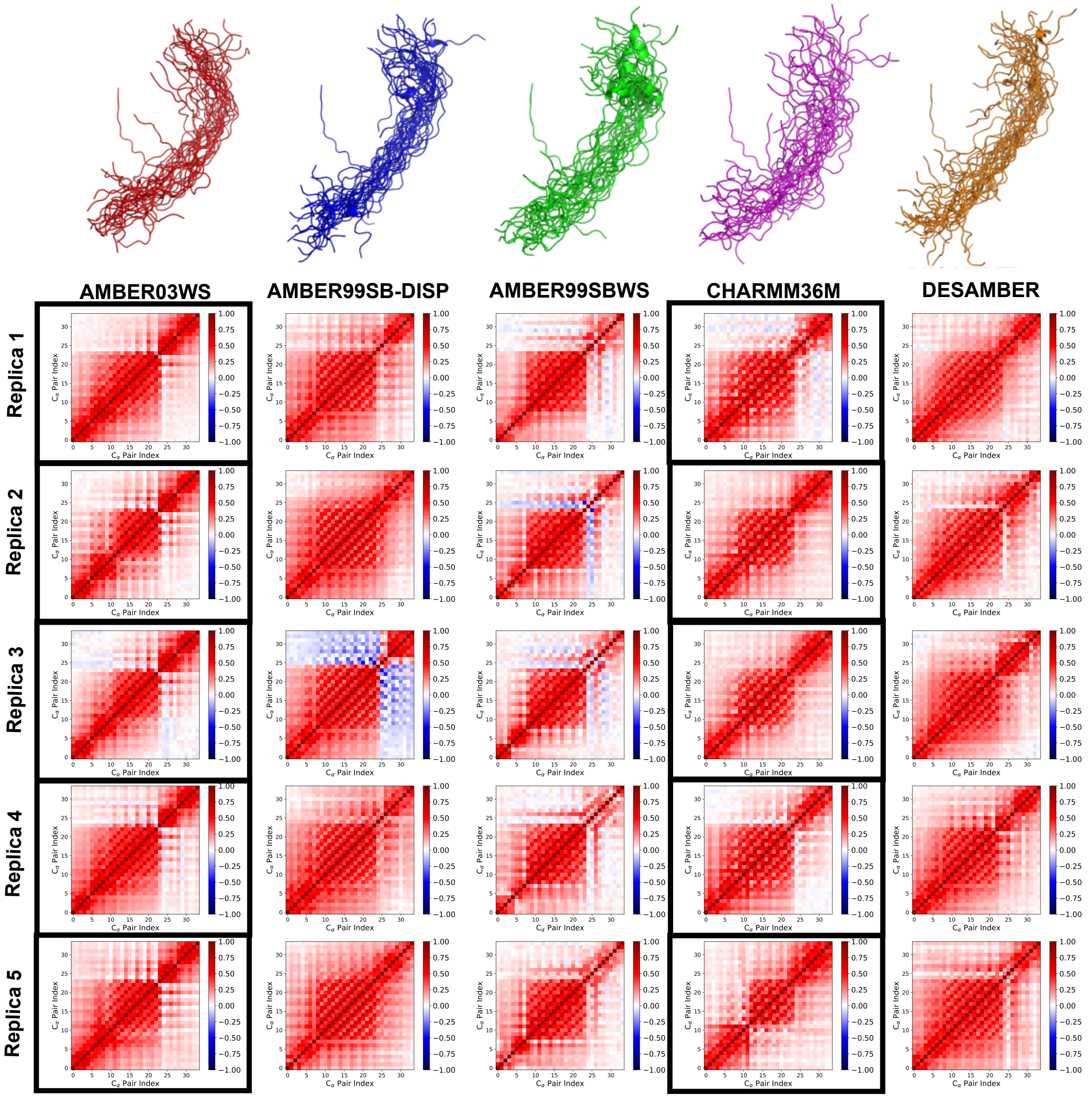

Figure S28: Consecutive Ca vector correlation maps generated from five different initial structures and force fields, along with representative snapshots for the charged peptide. Positive and negative values indicate positive and negative correlation respectively. The snapshots represent 5 overlayed frames from each force field for the same initial structure model of the peptide. The selected simulations from the quality evaluation are highlighted with the square.

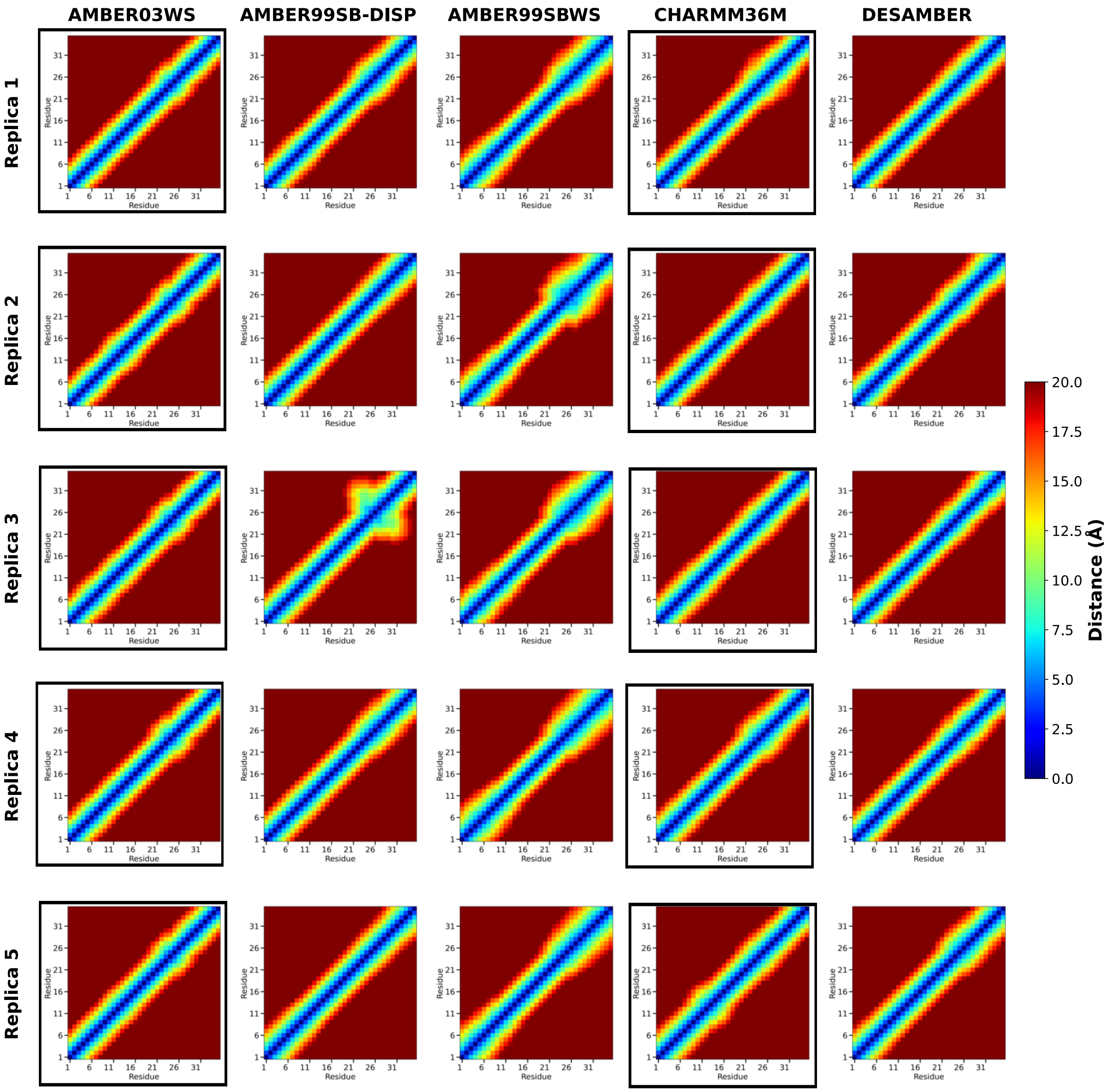

Figure S29: Distance maps for the charged peptide.

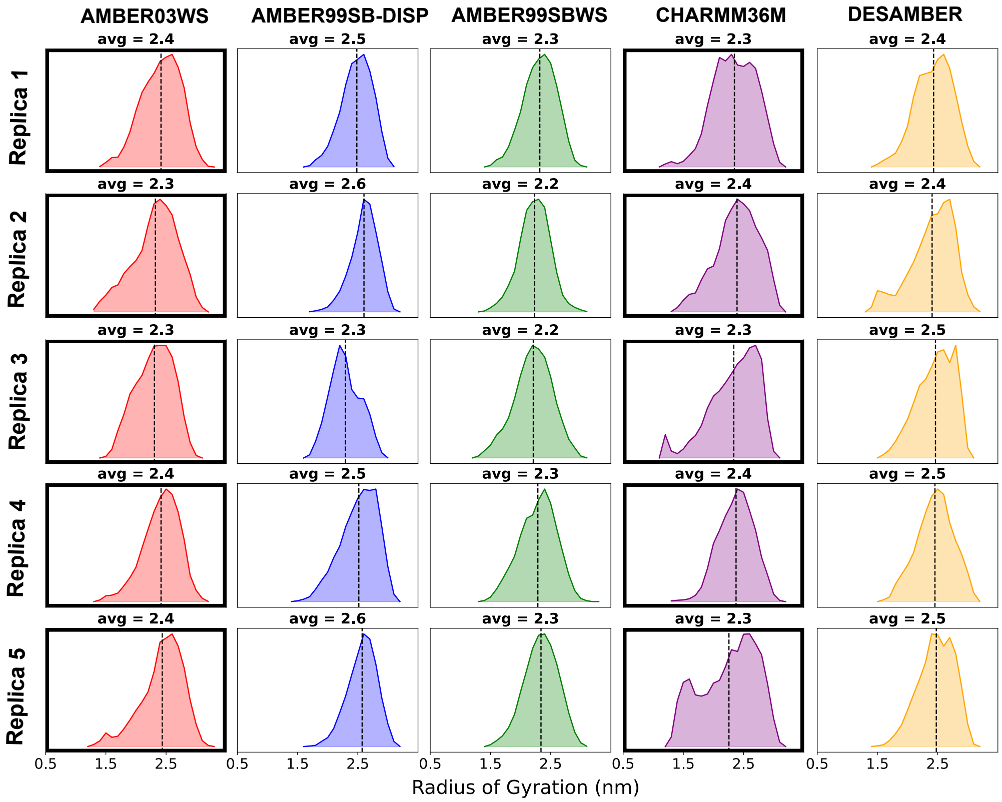

Figure S30: Radius of gyration distribution generated from five different initial structures and force fields for the charged peptide.

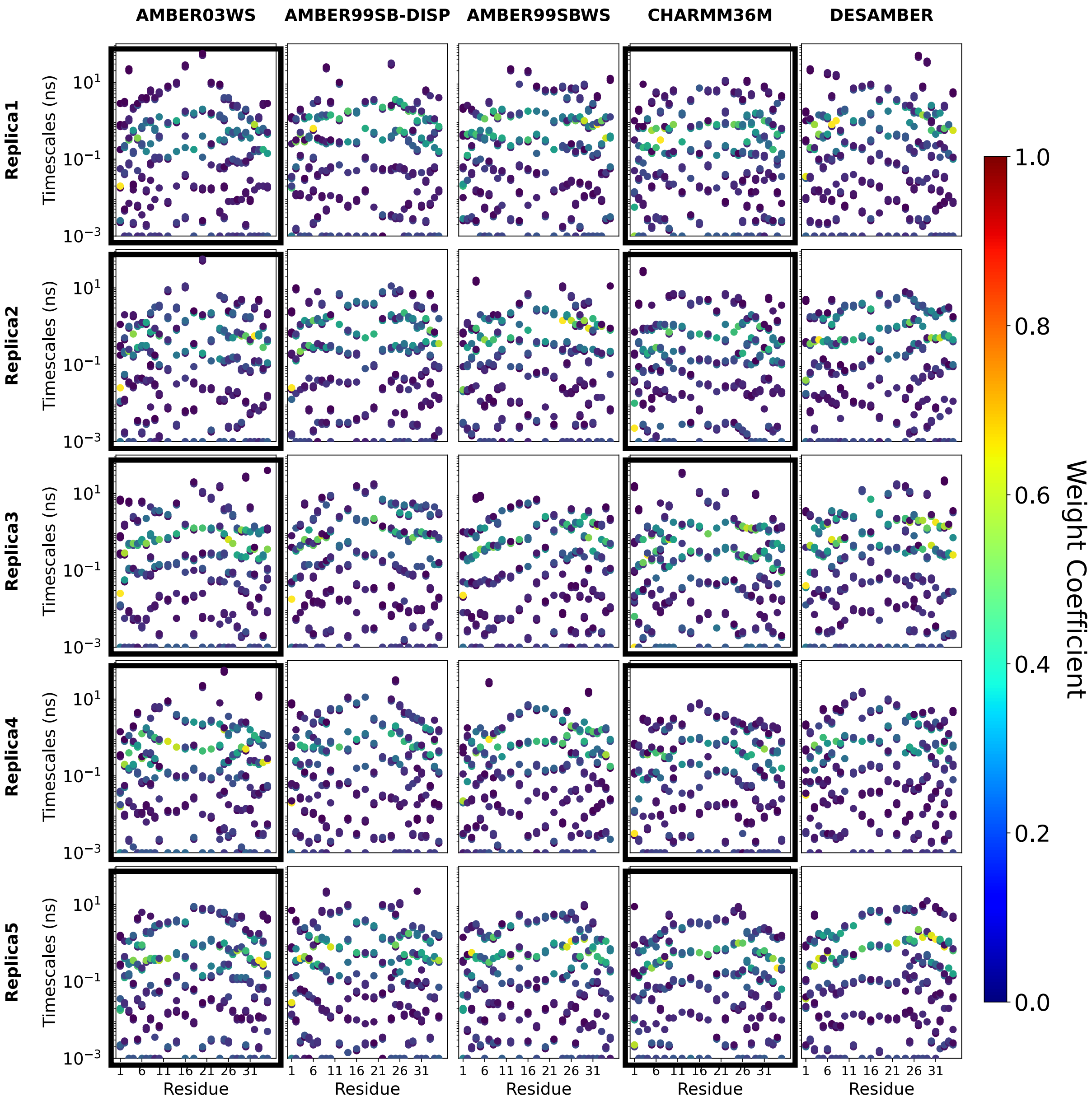

Figure S31: Timescales of backbone N-H bonds in the charged peptide.

Charged sequence with NaCl

Force field	Replica	R1 RMSD	R1 (%)	R2 RMSD	R2 (%)	hetNOE RMSD	hetNOE (%)	Sum (%)
AMBER03WS	replica02	0.073	110	0.14	100	0.11	100	310
AMBER03WS	replica01	0.094	150	0.17	120	0.12	110	380
CHARMM36M	replica02	0.085	130	0.29	200	0.19	180	510
CHARMM36M	replica01	0.078	120	0.33	230	0.18	170	520
CHARMM36M	replica03	0.094	140	0.24	170	0.21	200	520
AMBER99SB-DISP	replica01	0.14	220	0.46	320	0.28	260	810
AMBER99SB-DISP	replica02	0.13	200	0.55	390	0.26	240	830
AMBER03WS	replica04	0.15	230	0.71	500	0.11	100	830
CHARMM36M	replica05	0.093	140	0.75	530	0.21	200	870
AMBER99SBWS	replica02	0.19	290	0.63	440	0.35	320	1100
AMBER99SBWS	replica04	0.18	280	0.68	480	0.32	300	1100
AMBER99SBWS	replica05	0.16	250	0.78	550	0.31	290	1100
AMBER03WS	replica03	0.065	100	1.6	1100	0.11	100	1300
DESAMBER	replica04	0.18	280	1.2	840	0.34	320	1400
AMBER99SBWS	replica01	0.17	260	1.4	970	0.33	310	1500
AMBER99SB-DISP	replica04	0.2	300	1.2	850	0.35	330	1500
AMBER99SB-DISP	replica03	0.16	250	1.5	1100	0.31	290	1600
AMBER03WS	replica05	0.13	200	2.0	1400	0.14	130	1700
CHARMM36M	replica04	0.11	170	2.1	1400	0.2	190	1800
AMBER99SBWS	replica03	0.18	280	1.9	1400	0.29	270	1900
DESAMBER	replica05	0.14	210	3.3	2300	0.25	230	2800
DESAMBER	replica02	0.17	260	4.7	3300	0.33	310	3900
DESAMBER	replica03	0.17	270	4.7	3300	0.34	320	3900
AMBER99SB-DISP	replica05	0.17	270	4.8	3400	0.29	270	3900
DESAMBER	replica01	0.24	370	5.1	3600	0.39	360	4300

Figure S32: charged peptide in NaCl: RMSD and percentage difference to experiment values for relaxation rates R1, R2, and hetNOE. Ranking numbers are determined by their fraction of the lowest RMSD value for each spin relaxation rate. Simulations deviating less than 50 from the lowest RMSD for all spin relaxation rates are selected as best and highlighted in bold.

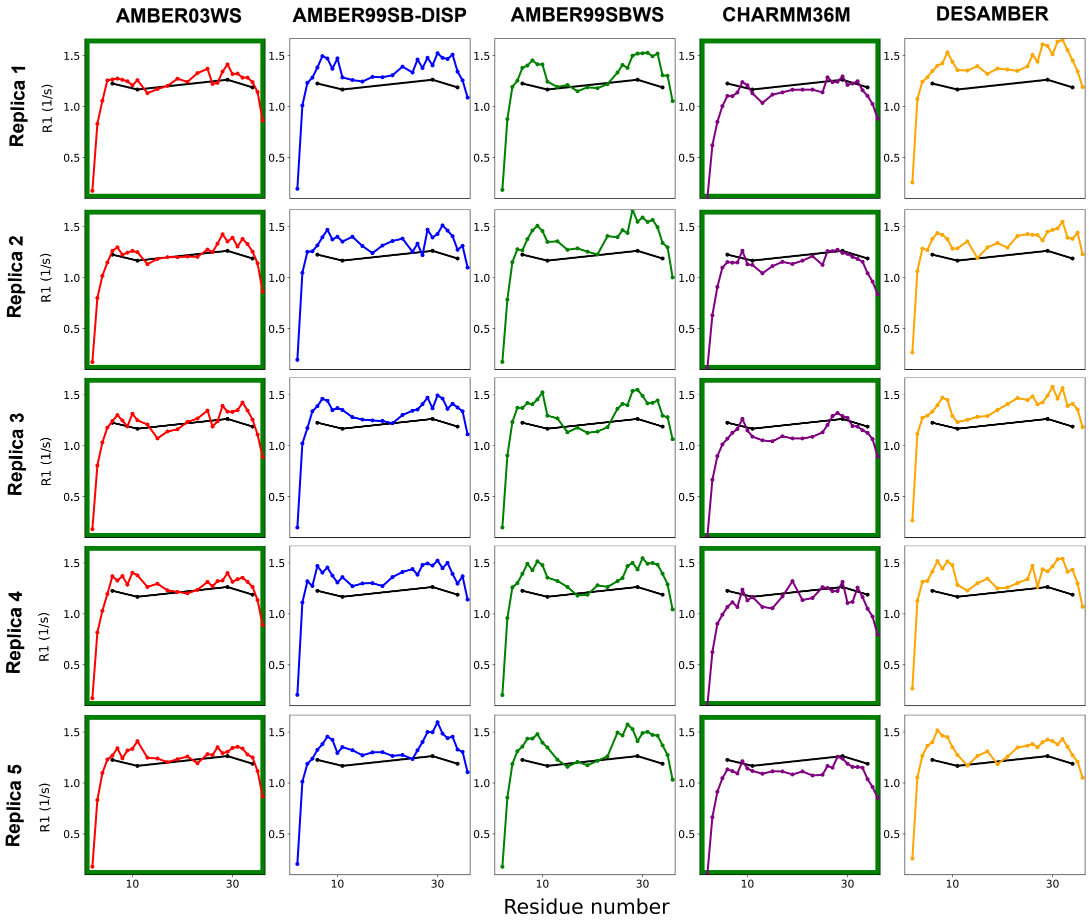

Figure S33: R1 relaxation rate plots for 25 simulations of different models and force fields of charged peptide in the presence of 150mM NaCl.

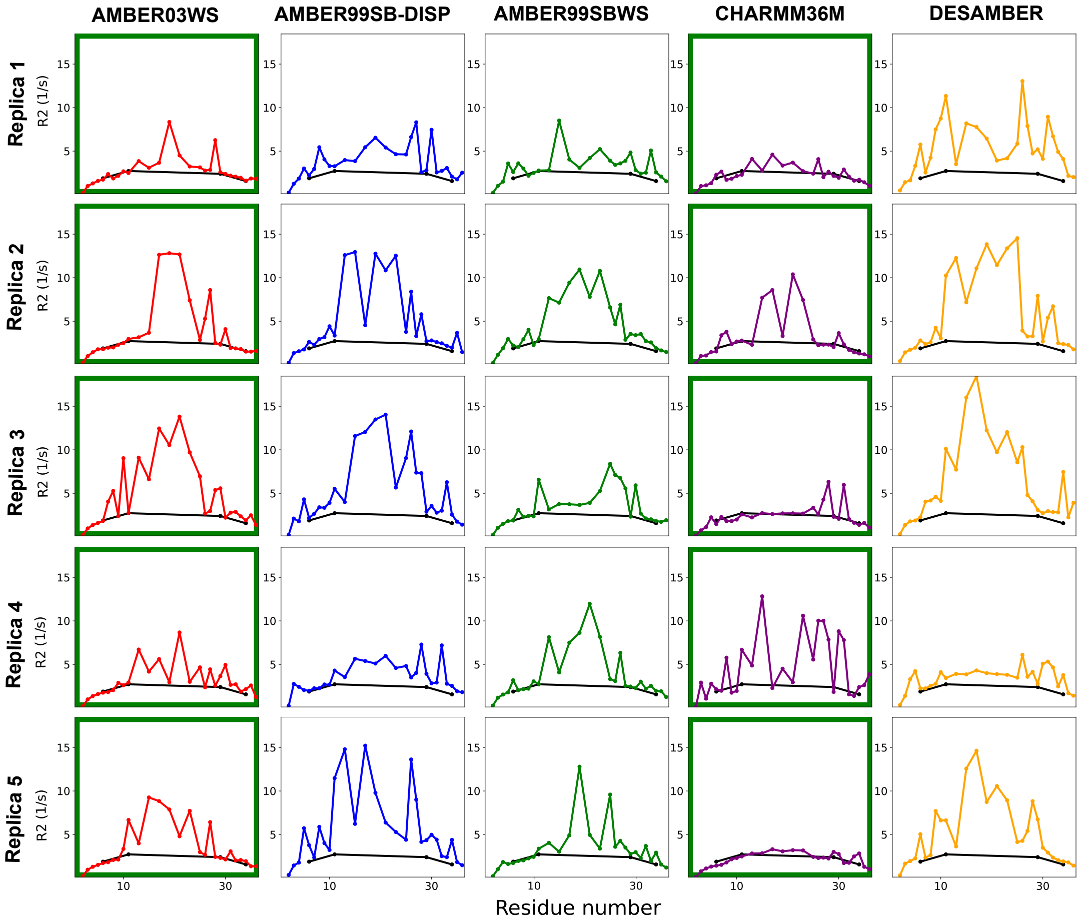

Figure S34: R2 relaxation rate plots for 25 simulations of different models and force fields of charged peptide in the presence of 150mM NaCl.

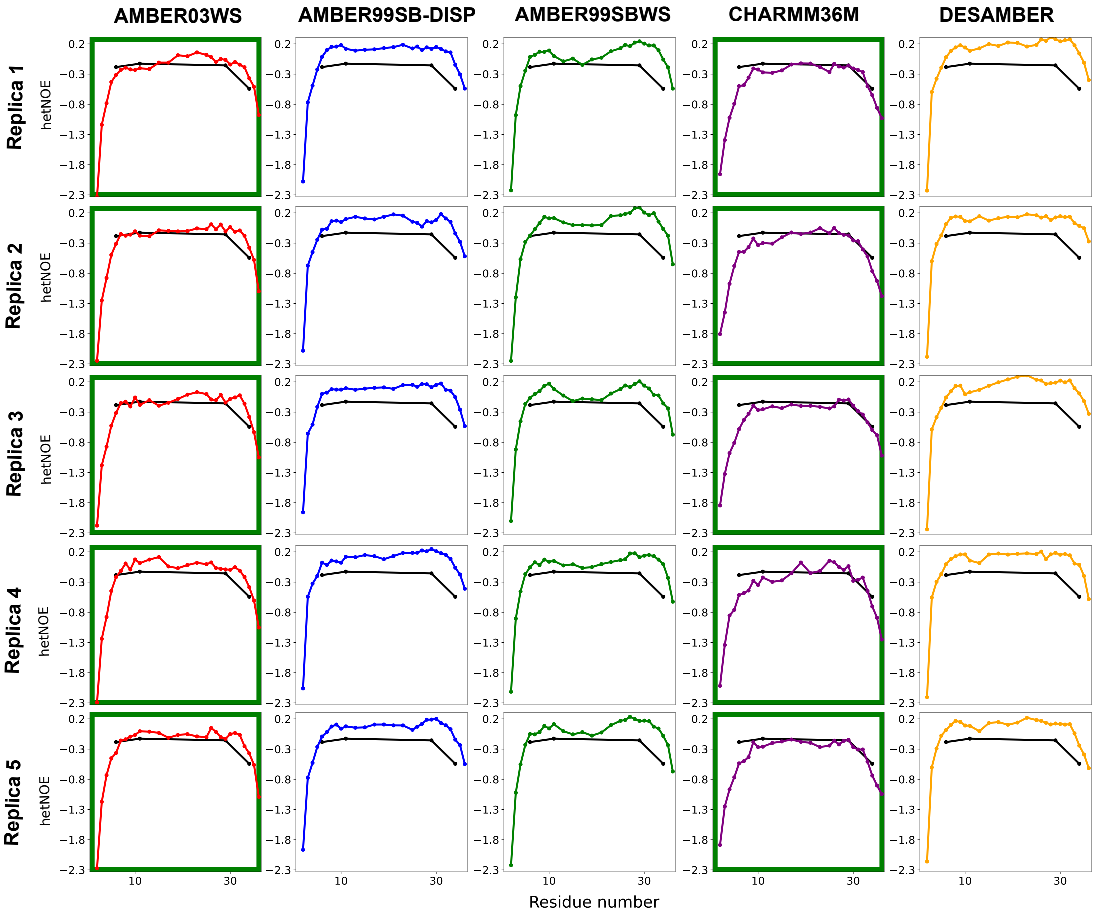

Figure S35: HetNoe relaxation time plots for 25 simulations of different models and force fields of Ton-derived peptide in the presence of 150mM NaCl.

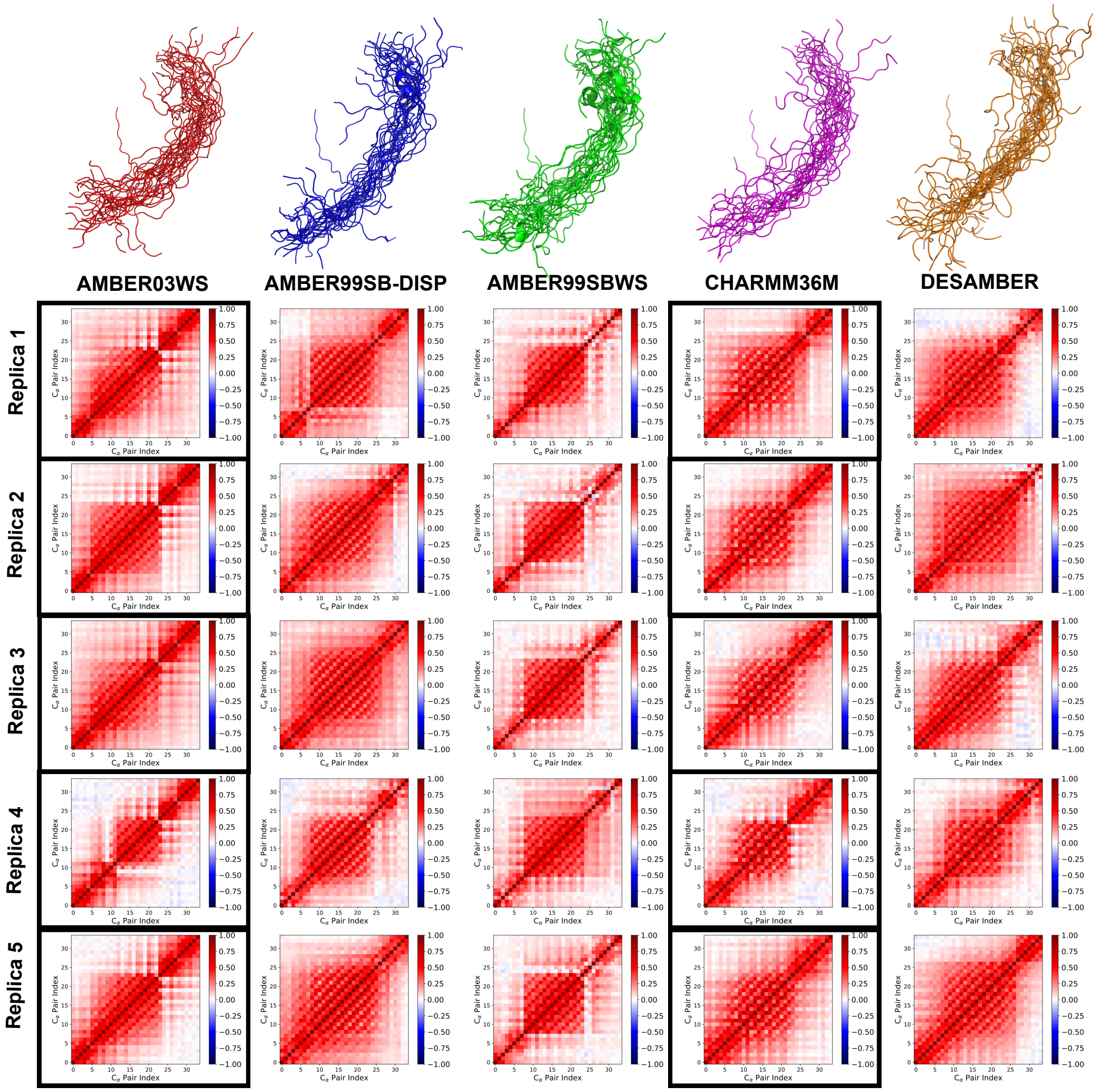

Figure S36: Consecutive Ca vector correlation maps generated from five different initial structures and force fields, along with representative snapshots for the charged peptide in 150mM NaCl. Positive and negative values indicate positive and negative correlation respectively. The snapshots represent 5 overlayed frames from each force field for the same initial structure model of the peptide. The selected simulations from the quality evaluation are highlighted with the square.

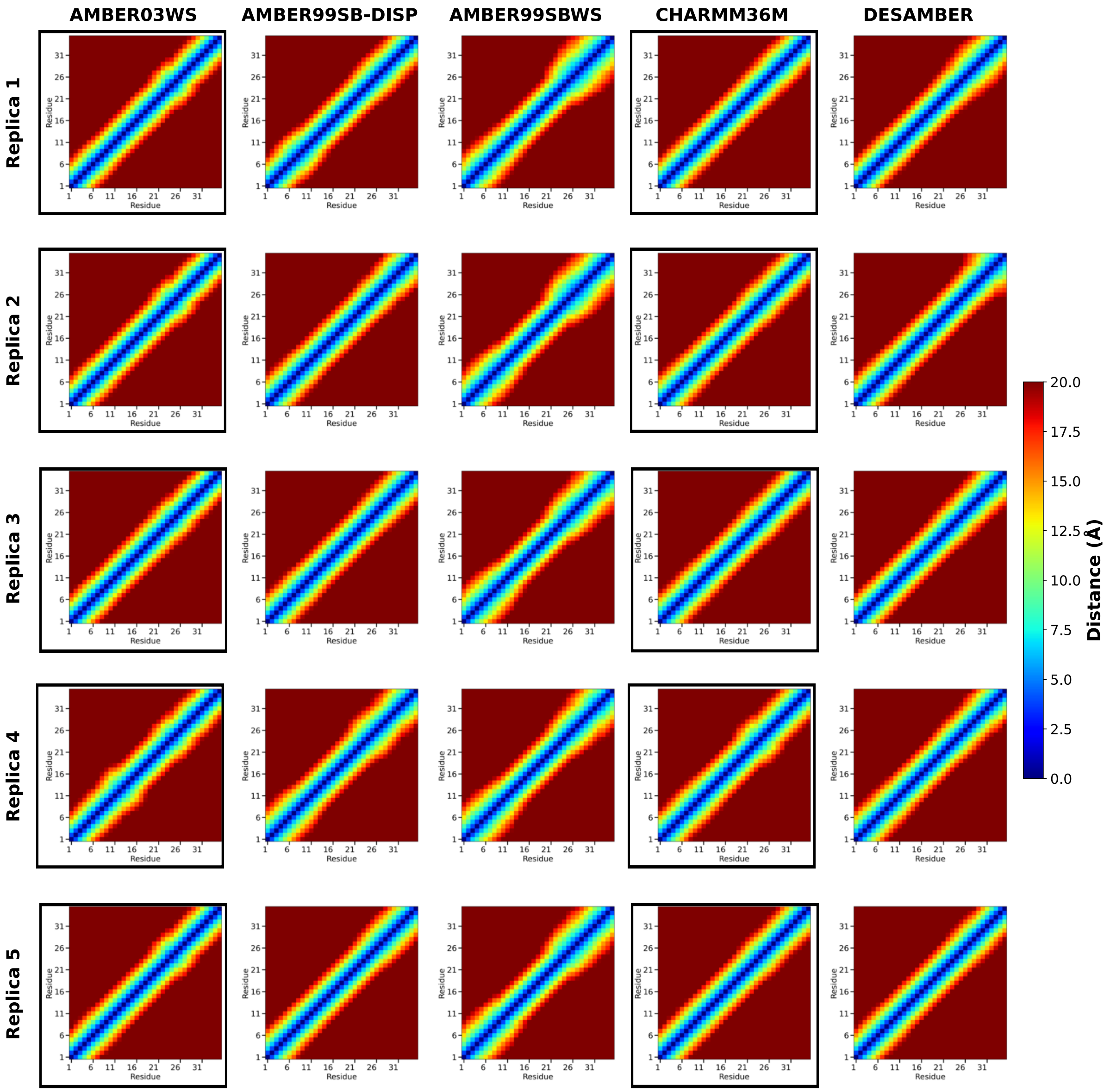

Figure S37: Distance maps for the charged peptide in 150mM NaCl.

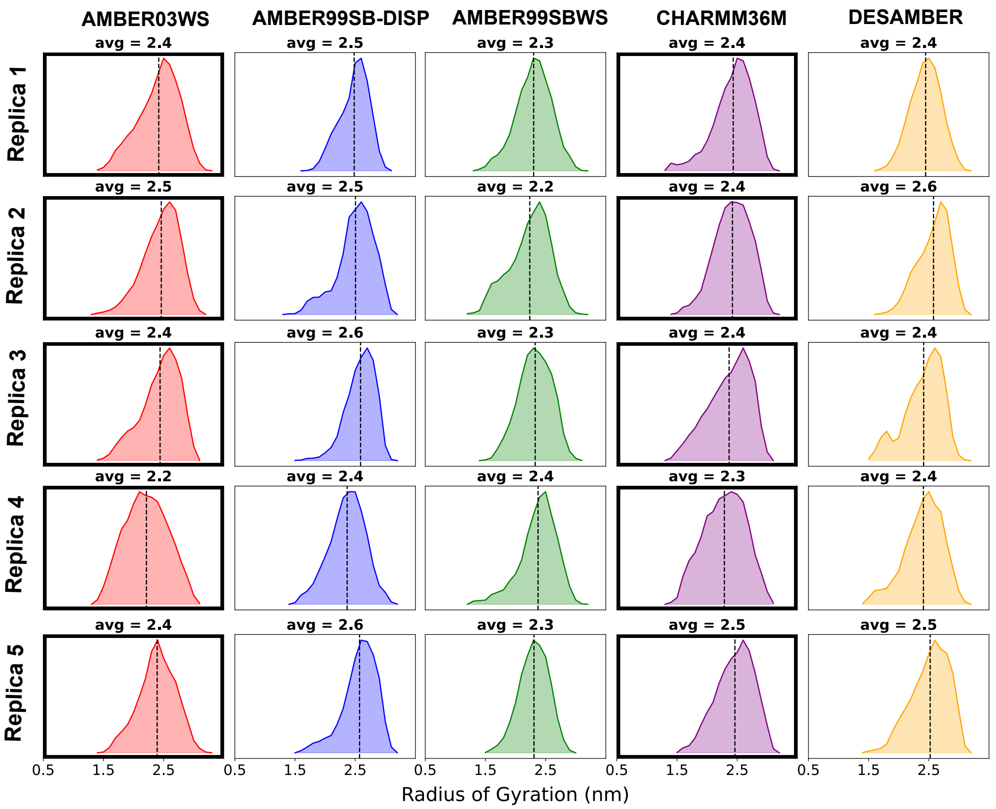

Figure S38: Radius of gyration distribution generated from five different initial structures and force fields for the charged peptide in the presence of 150mM NaCl.

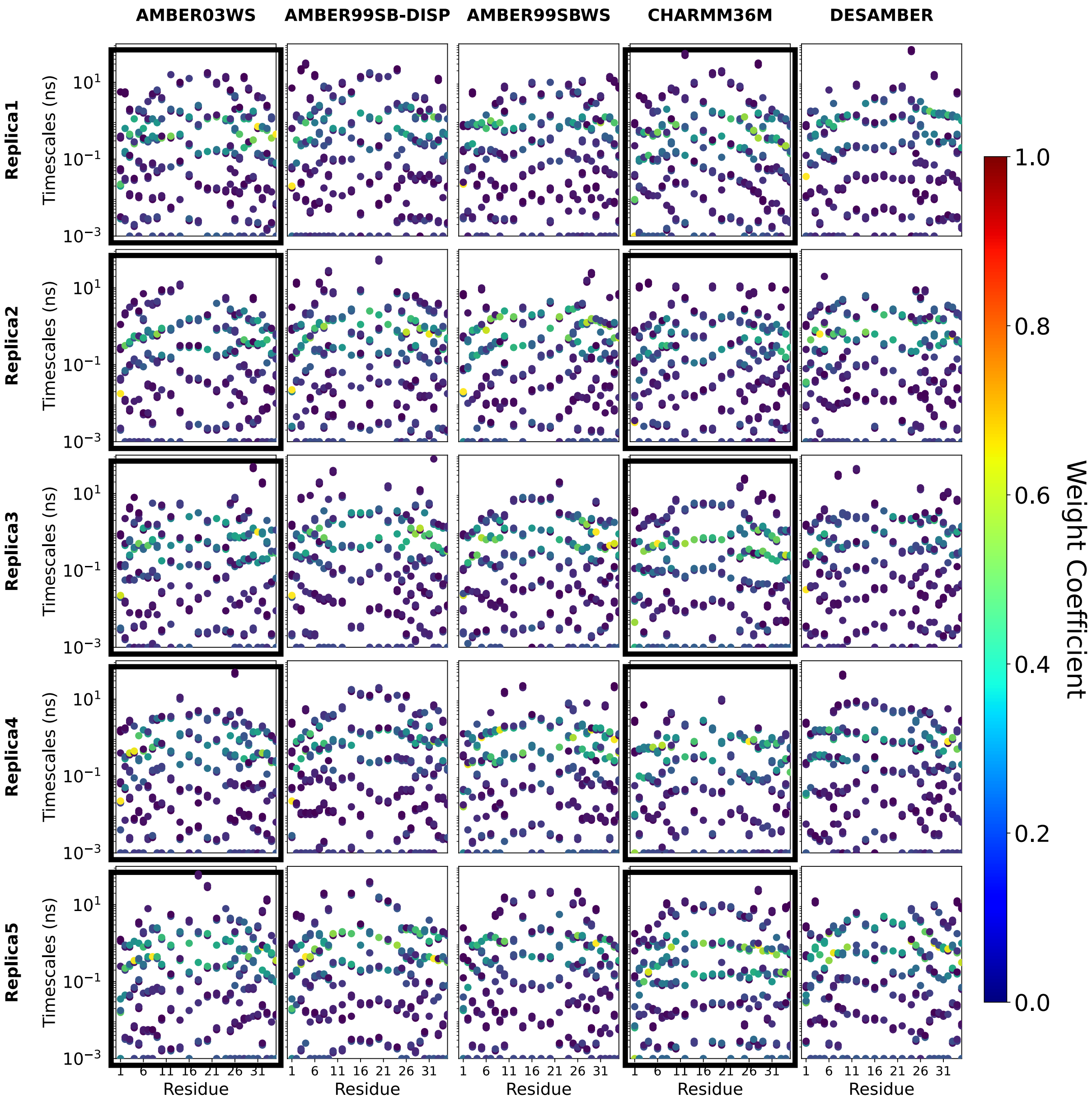

Figure S39: Timescales of of backbone N-H bonds in the charged peptide in 150mM NaCl.

Charged sequence with $CaCl_2$

Force field	Replica	R1 RMSD	R1 (%)	R2 RMSD	R2 (%)	hetNOE RMSD	hetNOE (%)	Sum (%)
CHARMM36M	replica04	0.054	120	0.17	100	0.1	210	430
AMBER99SB-DISP	replica04	0.066	150	0.5	290	0.071	140	580
AMBER99SB-DISP	replica01	0.096	220	0.31	180	0.11	210	610
DESAMBER	replica02	0.06	130	0.68	400	0.049	100	630
DESAMBER	replica03	0.087	190	0.59	350	0.082	170	710
CHARMM36M	replica03	0.13	290	0.2	120	0.21	430	830
CHARMM36M	replica05	0.09	200	0.59	340	0.23	460	1000
CHARMM36M	replica02	0.045	100	0.89	520	0.22	450	1100
DESAMBER	replica05	0.09	200	1.2	700	0.1	210	1100
CHARMM36M	replica01	0.065	150	1.2	690	0.24	480	1300
AMBER99SB-DISP	replica02	0.094	210	1.5	850	0.11	220	1300
AMBER03WS	replica01	0.23	520	0.8	470	0.27	550	1500
AMBER03WS	replica04	0.28	630	1.0	580	0.28	580	1800
AMBER99SBWS	replica04	0.28	640	1.1	660	0.32	640	1900
AMBER99SB-DISP	replica05	0.24	530	1.6	940	0.28	560	2000
AMBER99SB-DISP	replica03	0.12	270	2.6	1500	0.13	270	2100
AMBER99SBWS	replica03	0.27	600	1.3	740	0.37	740	2100
AMBER03WS	replica02	0.25	550	2.2	1300	0.24	490	2300
AMBER99SBWS	replica02	0.17	390	2.5	1400	0.28	570	2400
DESAMBER	replica01	0.11	250	3.5	2100	0.11	230	2500
DESAMBER	replica04	0.065	150	3.8	2200	0.084	170	2600
AMBER03WS	replica05	0.12	260	3.6	2100	0.16	320	2700
AMBER99SBWS	replica05	0.33	750	1.9	1100	0.41	830	2700
AMBER99SBWS	replica01	0.27	600	2.7	1600	0.31	630	2800
AMBER03WS	replica03	0.28	630	2.8	1600	0.33	680	2900

Figure S40: charged peptide in $CaCl_2$: RMSD and percentage difference to experiment values for relaxation rates R1, R2 and hetNOE. Ranking numbers are determined by their fraction of the lowest RMSD value for each spin relaxation rate. Simulations deviating less than 50 from the lowest RMSD for all spin relaxation rates are selected as best and highlighted in bold

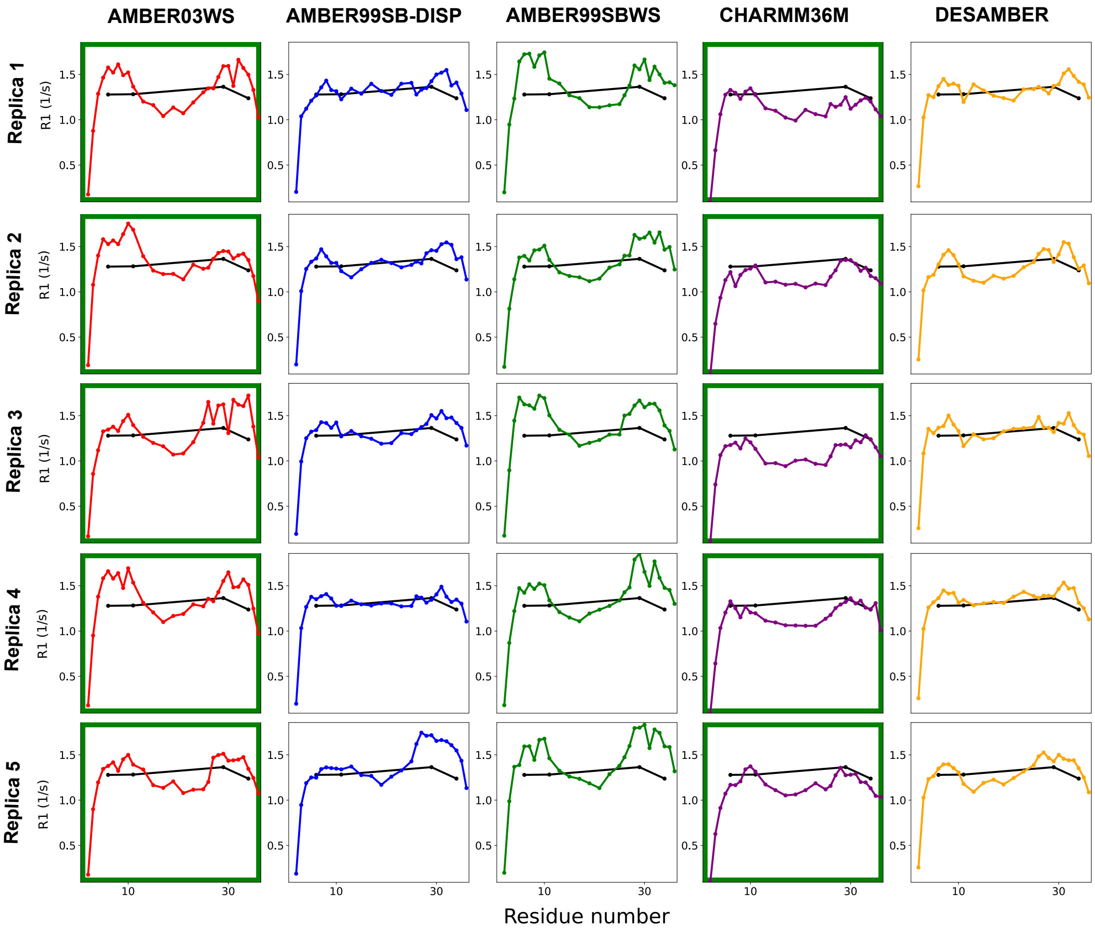

Figure S41: R1 relaxation rate plots for 25 simulations of different models and force fields of charged peptide in the presence of 10mM $CaCl_2$.

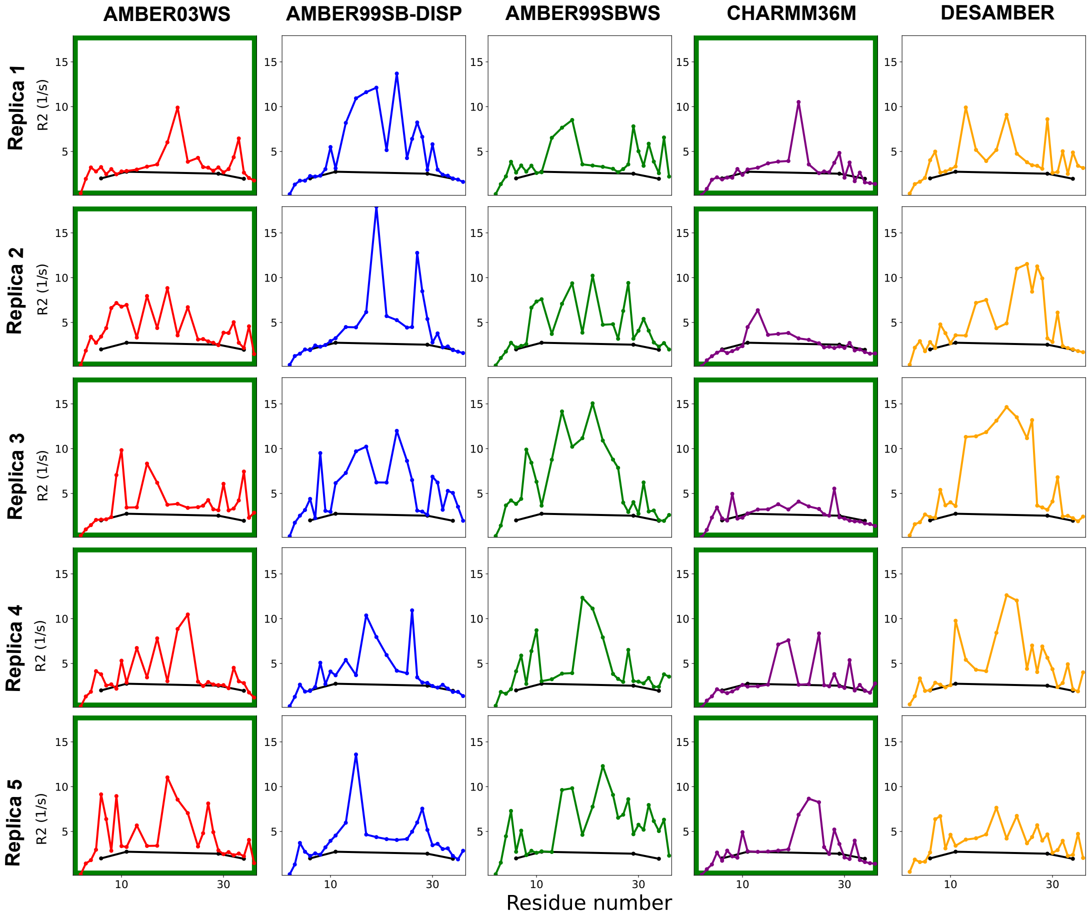

Figure S42: R2 relaxation rate plots for 25 simulations of different models and force fields of charged peptide in the presence of 10mM $CaCl_2$.

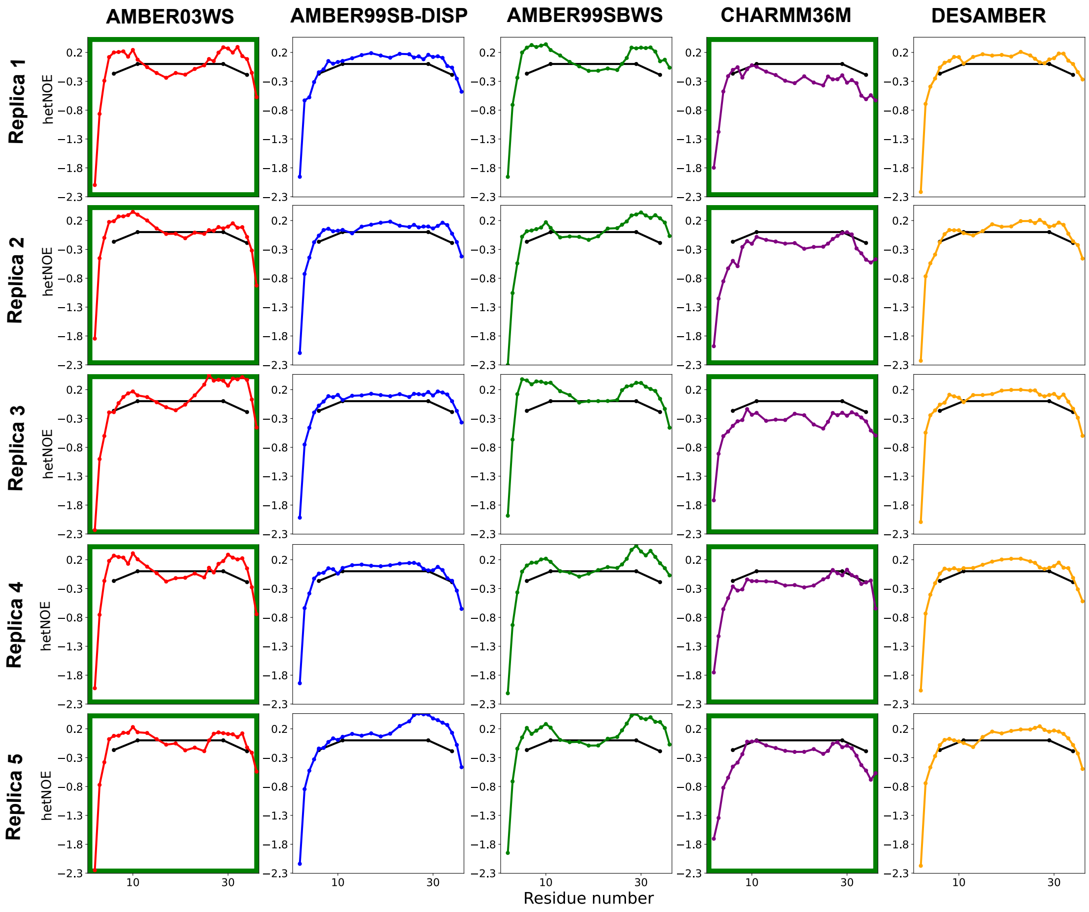

Figure S43: HetNoe relaxation time plots for 25 simulations of different models and force fields of Ton-derived peptide in the presence of 10mM $CaCl_2$.

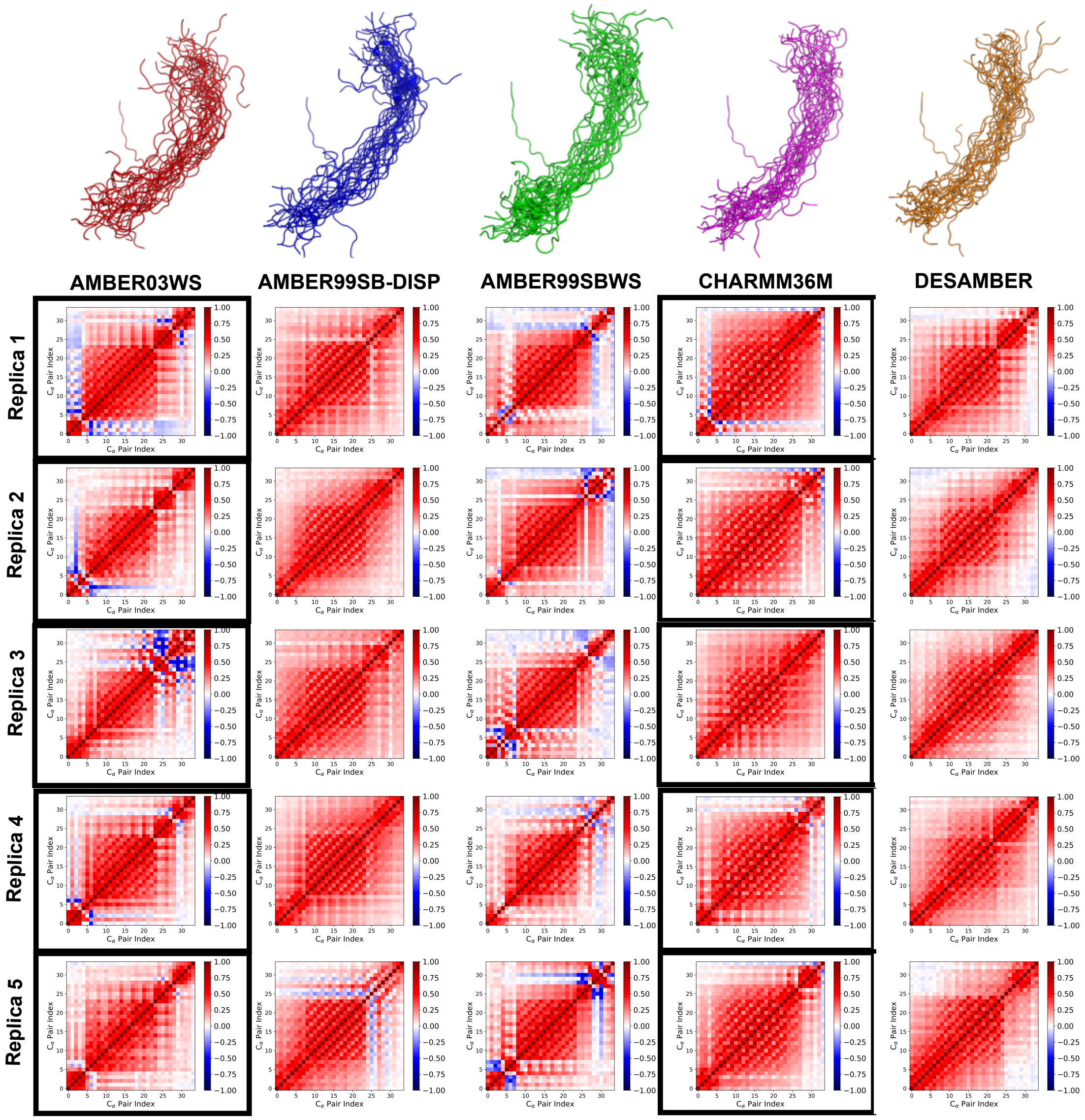

Figure S44: Consecutive Ca vector correlation maps generated from five different initial structures and force fields, along with representative snapshots for the charged peptide in 10mM $CaCl_2$. Positive and negative values indicate positive and negative correlation respectively. The snapshots represent 5 overlayed frames from each force field for the same initial structure model of the peptide. The selected simulations from the quality evaluation are highlighted with the square.

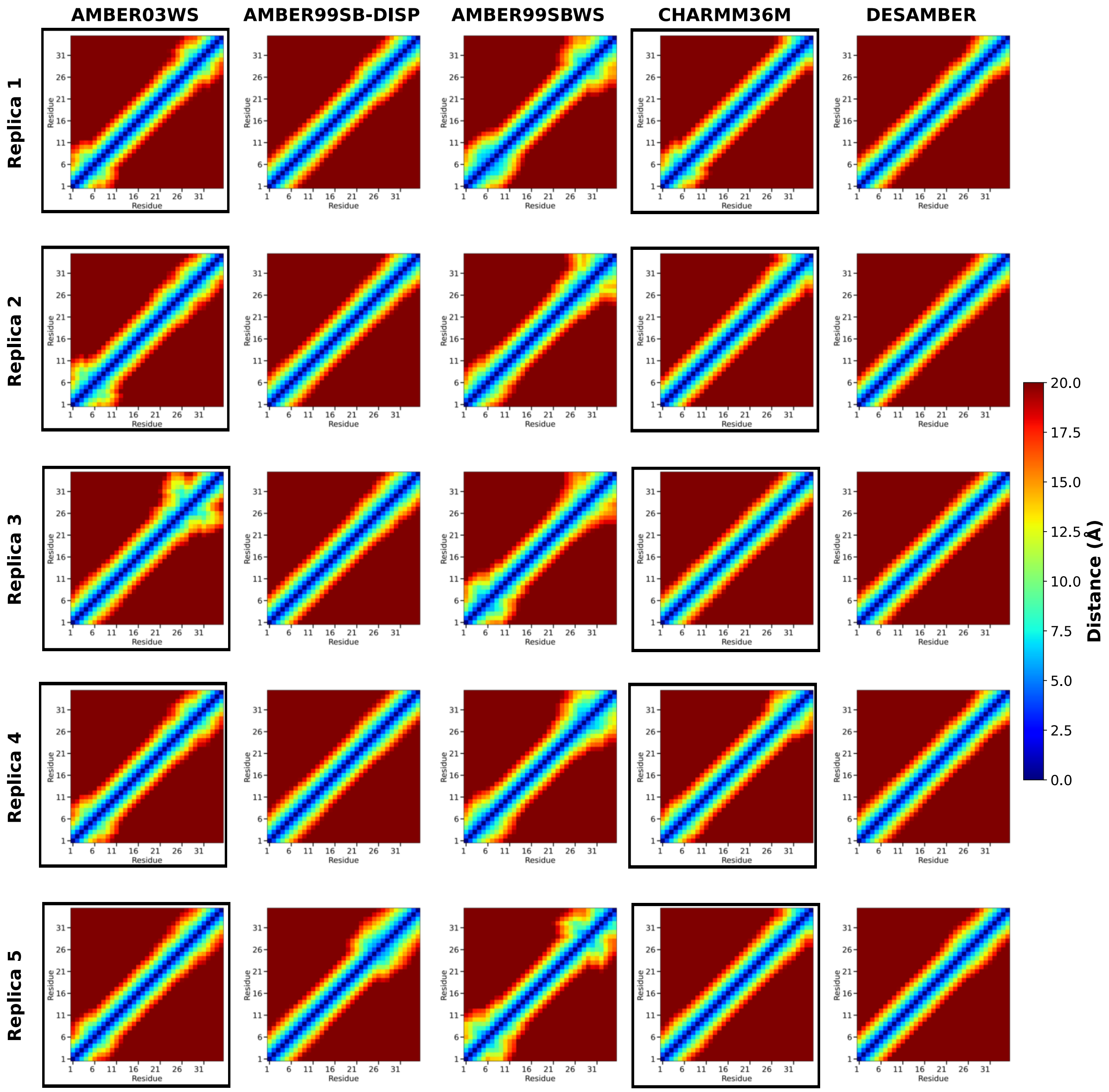

Figure S45: Distance maps for charged peptide in 10mM $CaCl_2$.

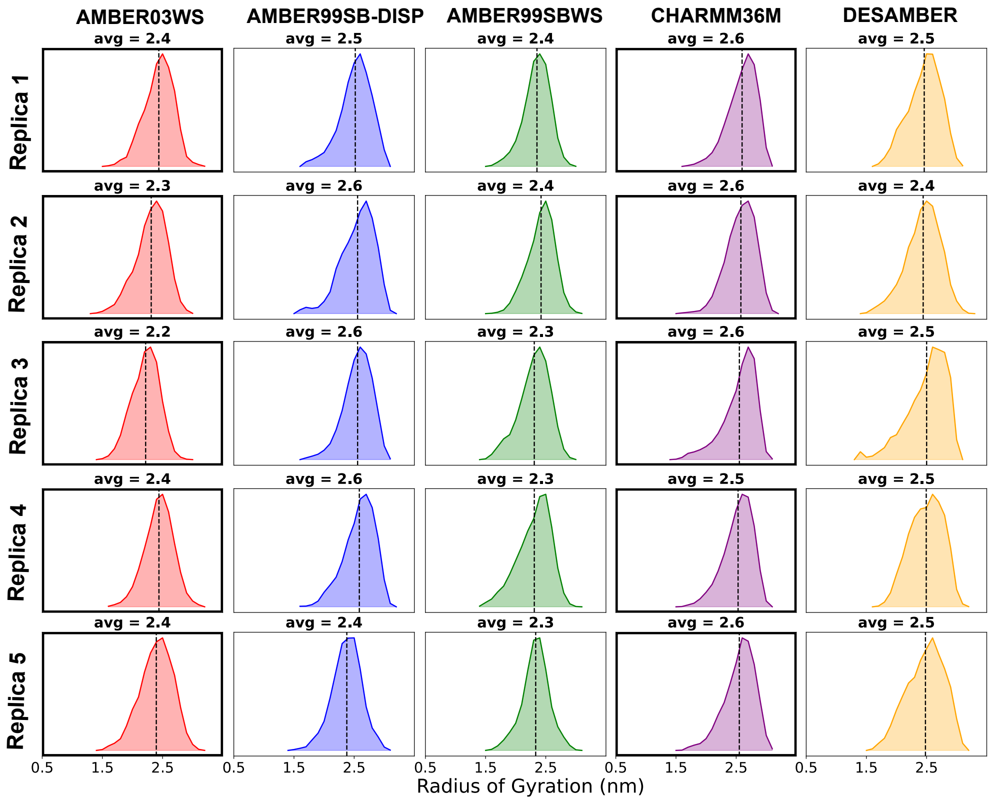

Figure S46: Radius of gyration distribution generated from five different initial structures and force fields for the charged peptide in the presence of 10mM $CaCl_2$.

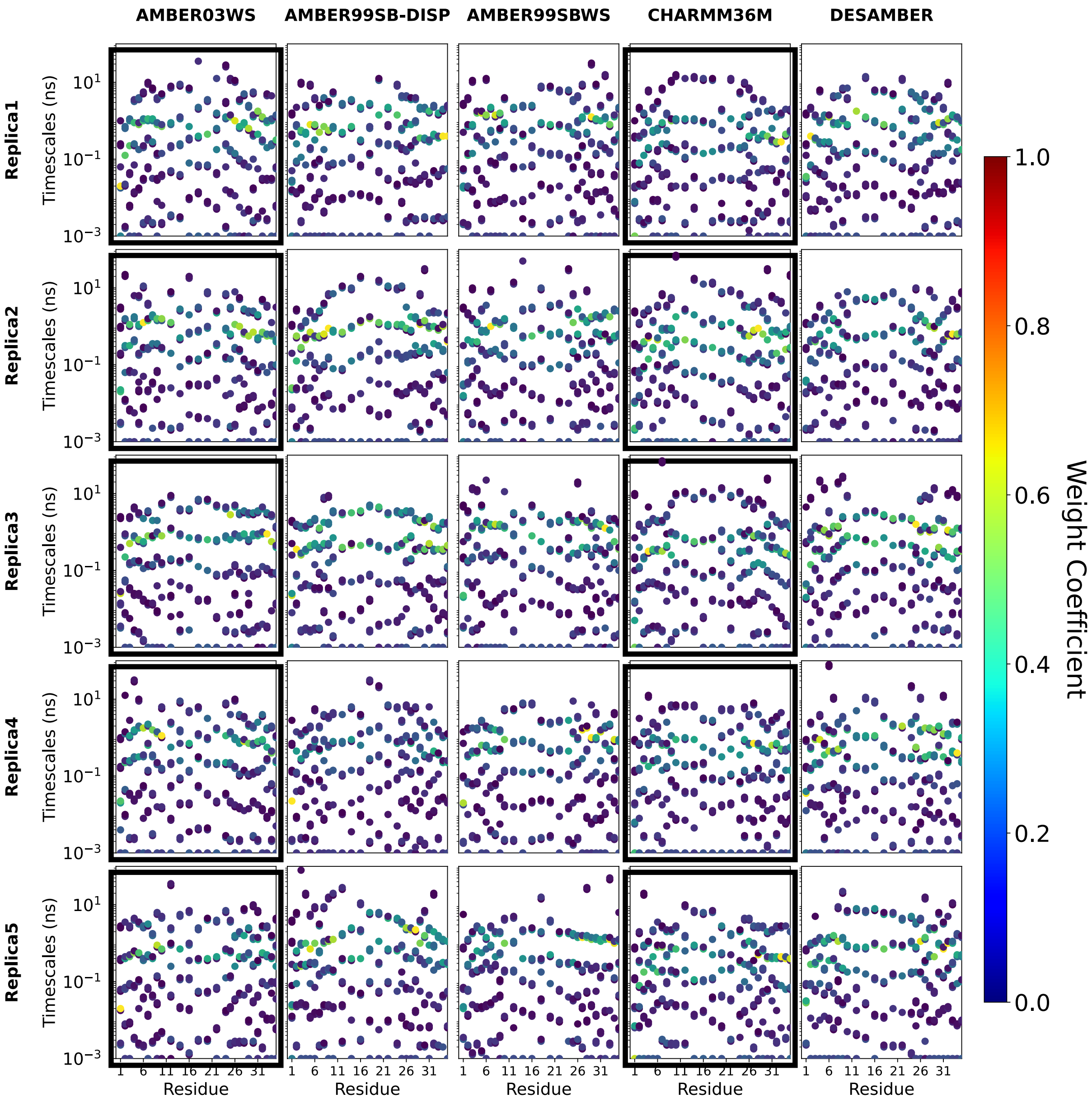

Figure S47: Timescales of of backbone N-H bonds in the charged peptide in 10mM CaCl2.

Effect of sodium and calcium ions on the charged peptide

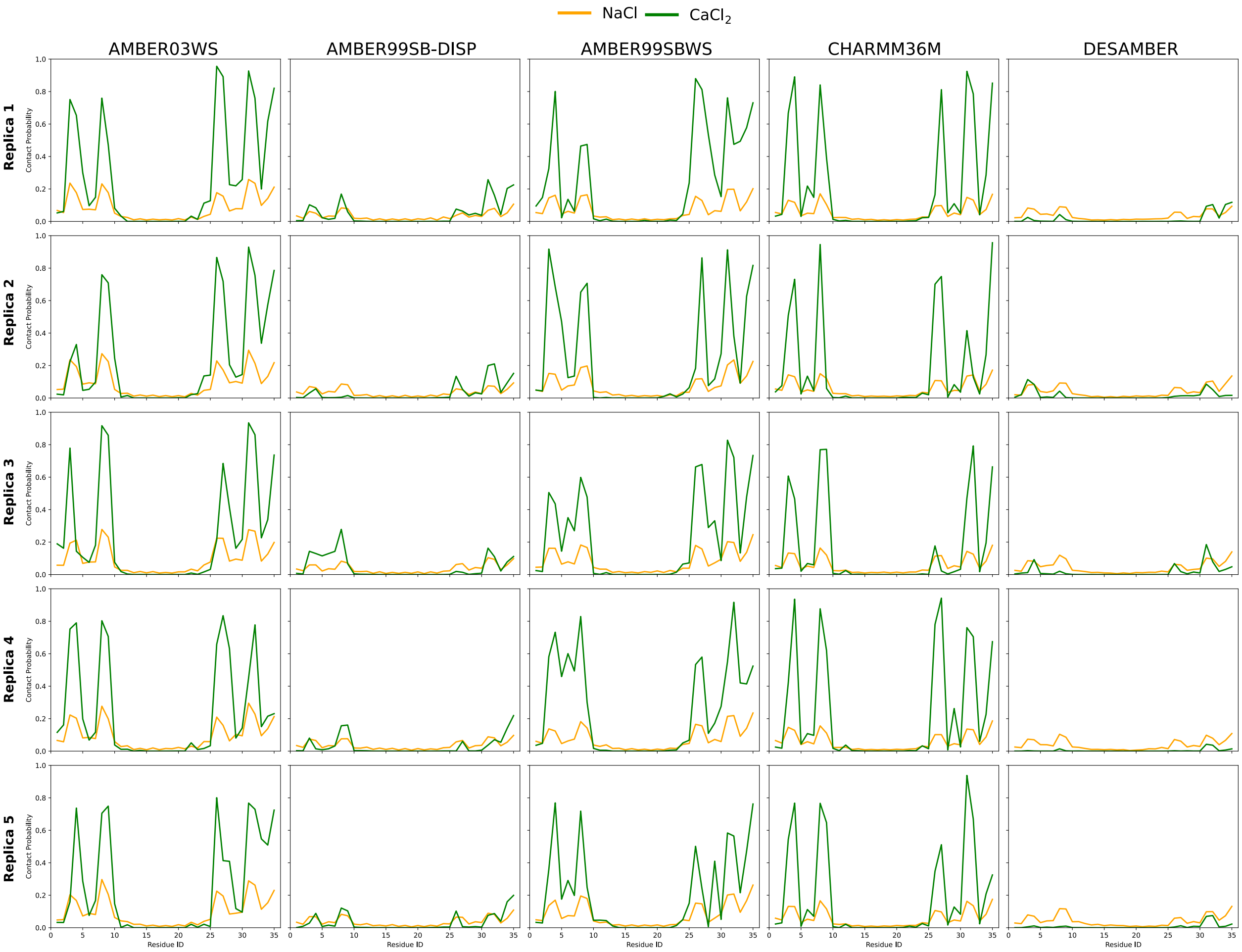

Figure S48: Contact probabilities of calcium and sodium ions with the glutamic acid pairs of the charged peptide over all the simulations.

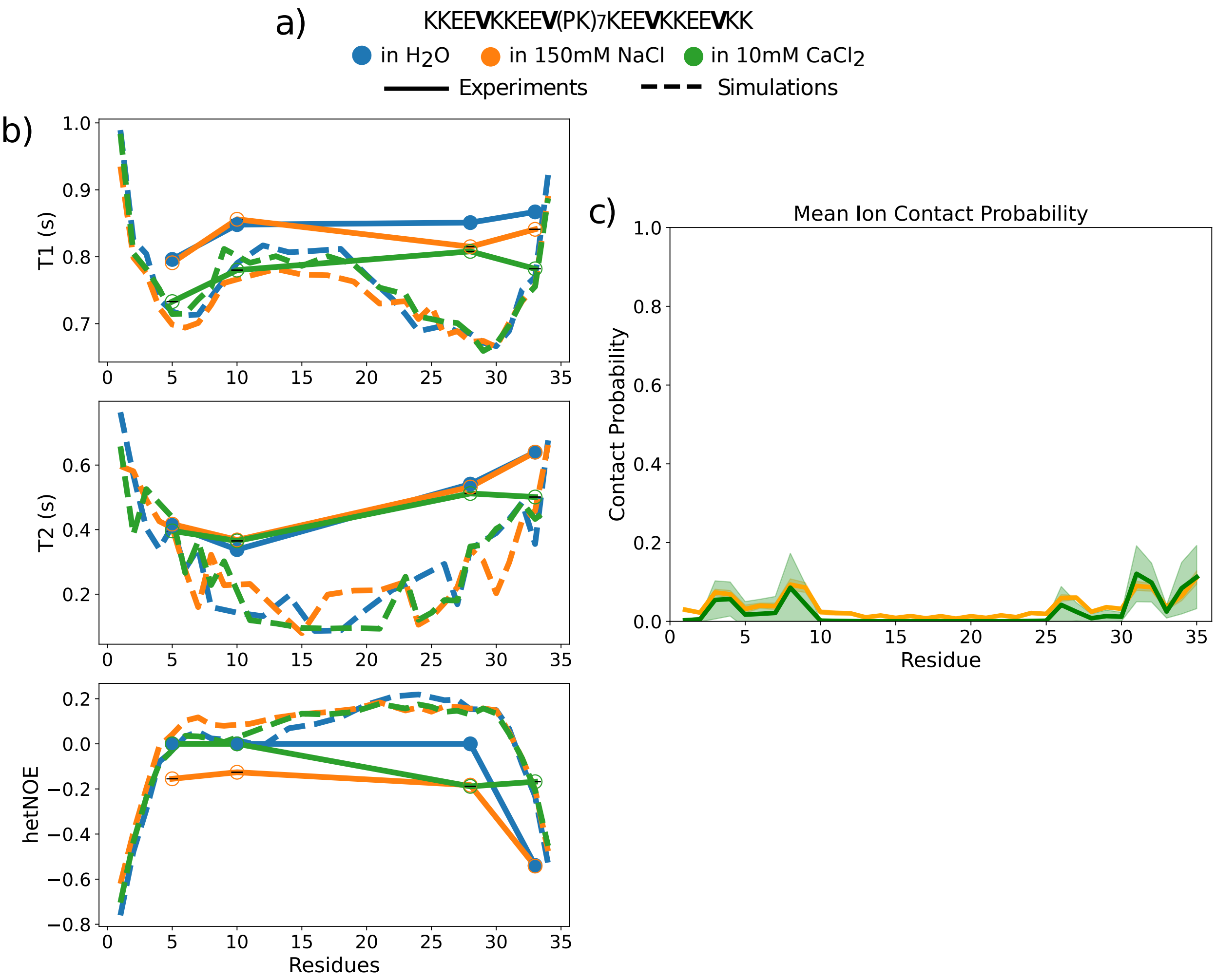

Figure S49: (a) Amino acid sequences of the studied peptide with ^{15}N labeled residues shown in bold. (b) Spin relaxation times from experiments and the AMBER99SB-DISP and DESAMBER simulations. Spin relaxation times from MD simulations of residue 1 was removed due to scaling reasons; Residue one exhibits very fast dynamics leading to long times. (c) Average contact probabilities of calcium and sodium ions with the glutamic acid pairs of the charged peptide over the AMBER99SB-DISP and DESAMBER simulations. The errors were calculated as standard mean errors.

Simulation data availability

Table S1: All simulation data is available in Zenodo repositories.

Protein	Force Field	DOI
$(GGS)_3$	AMBER03WS	10.5281/zenodo.16925399
$(GGS)_3$	AMBER99SB-DISP	10.5281/zenodo.16926107
$(GGS)_3$	AMBER99SBWS	10.5281/zenodo.16926198
$(GGS)_3$	CHARMM36M	10.5281/zenodo.16926256
$(GGS)_3$	DESAMBER	10.5281/zenodo.16926284
$(GPS)_3$	AMBER03WS	10.5281/zenodo.16926303
$(GPS)_3$	AMBER99SB-DISP	10.5281/zenodo.16926459
$(GPS)_3$	AMBER99SBWS	10.5281/zenodo.16926477
$(GPS)_3$	CHARMM36M	10.5281/zenodo.16926503
$(GPS)_3$	DESAMBER	10.5281/zenodo.16926510
$K(AP)_5K$	AMBER03WS	10.5281/zenodo.16926564
$K(AP)_5K$	AMBER99SB-DISP	10.5281/zenodo.16928503
$K(AP)_5K$	AMBER99SBWS	10.5281/zenodo.16928511
$K(AP)_5K$	CHARMM36M	10.5281/zenodo.16928545
$K(AP)_5K$	DESAMBER	10.5281/zenodo.16928586
Charged sequence	AMBER03WS	10.5281/zenodo.16928640
Charged sequence	AMBER99SB-DISP	10.5281/zenodo.16928790
Charged sequence	AMBER99SBWS	10.5281/zenodo.16929062
Charged sequence	CHARMM36M	10.5281/zenodo.16929122
Charged sequence	DESAMBER	10.5281/zenodo.16929115
Charged sequence with NaCl	AMBER03WS	10.5281/zenodo.16929574
Charged sequence with NaCl	AMBER99SB-DISP	10.5281/zenodo.16931819
Charged sequence with NaCl	AMBER99SBWS	10.5281/zenodo.16932024
Charged sequence with NaCl	CHARMM36M	10.5281/zenodo.16932035
Charged sequence with NaCl	DESAMBER	10.5281/zenodo.16932041
Charged sequence with $CaCl_2$	AMBER03WS	10.5281/zenodo.16932078
Charged sequence with $CaCl_2$	AMBER99SB-DISP	10.5281/zenodo.16932100
Charged sequence with $CaCl_2$	AMBER99SBWS	10.5281/zenodo.16932128
Charged sequence with $CaCl_2$	CHARMM36M	10.5281/zenodo.16932180
Charged sequence with $CaCl_2$	DESAMBER	10.5281/zenodo.16932203
